# The Adverse Reactions of Lianhua Qingwen Capsule/Granule Compared With Conventional Drug in Clinical Application: A Meta-Analysis

**DOI:** 10.3389/fphar.2022.764774

**Published:** 2022-01-27

**Authors:** Caiyun Hu, Bin He, Fengfeng Gong, Mingming Liang, Dongdong Zhao, Guoliang Zhang

**Affiliations:** ^1^ Department of Scientific Research, The First Affiliated Hospital of Anhui University of Traditional Chinese Medicine, Anhui, China; ^2^ Fuyang Hospital of Anhui Medical University, Anhui, China; ^3^ Epidemiology and Health Statistics, School of Public Health, Anhui Medical University, Anhui, China; ^4^ Medical Department, The First Affiliated Hospital of Anhui Medical University, Anhui, China; ^5^ Infectious Disease Department, The First Affiliated Hospital of Anhui University of Traditional Chinese Medicine, Anhui, China

**Keywords:** Lianhua Qingwen, adverse reaction, COVID-19, influenza, traditional Chinese medicine, meta-analysis

## Abstract

**Objectives:** Lianhua Qingwen capsule/granule (LHQW) is an innovative patented traditional Chinese medicine with potential curative effects on respiratory diseases. However, no consensus has been reached on the security of LHQW to date. The current meta-analysis was performed to evaluate the safety profile of LHQW in relation to conventional drugs (PROSPERO CRD-42020224180).

**Methods:** Comprehensive document retrieval was performed from both English and Chinese databases. Results were reported as risk ratio (RR) with 95% confidence interval (CI). Subgroup, sensitivity and meta-regression analyses were conducted to explore the possible sources of heterogeneity across eligible studies.

**Results:** In total, 217 experimental studies were included. For pooled studies, the incidence of adverse reactions was lower in the LHQW group than the conventional drug group (RR = 0.63, 95% CI = 0.58–0.69, *p* < 0.001). In the evaluation of treating disease, significant reduced incidence of adverse reactions during treatment of influenza A (H1N1) and influenza were detected in the LHQW group. In the evaluation of security indexes, LHQW group has a reduced incidence of respiratory system damage, skin and its appendages injury, nervous system damage and gastrointestinal system damage, along with other adverse reactions. Subgroup analysis additionally revealed a reduced incidence of some adverse reactions in the LHQW group compared to the conventional drug group (Rash of skin and its appendage damage, dizziness or headache owing to nervous system damage, nausea or vomiting from gastrointestinal system damage and resurgence of disease from other adverse reactions).

**Conclusion:** The current study provides potential a reference for the security of LHQW. Further long-term high-quality studies are essential to validate our conclusions.

**Systematic Review Registration:** https://clinicaltrials.gov/, CRD-42020224180

## Introduction

Lianhua Qingwen capsule/granule (LHQW) is obtained by combining Ma Xing Shigan Decoction with Yinqiao Powder and widely used in the clinical treatment of respiratory disorders (Liu et al., 2010). LHQW was initially approved by the China Food and Drug Administration (CFDA) in 2004 and represents the first traditional Chinese medicine that passed the rapid drug approval channel of CFDA for treatment of severe acute respiratory syndrome (SARS). As Chinese Pharmacopoeia records, LHQW consists of 13 ingredients: *Forsythia suspensa* (Thunb.) Vahl [Oleaceae; *Forsythiae Fructus*] (255 g), *Lonicera japonica* Thunb. [Caprifoliaceae; *Lonicerae Japonicae Flos*] (255 g), *Ephedra sinica* Stapf [Ephedraceae; *Ephedrae Botanical drug*] (85 g), *Prunus armeniaca* L. [Rosaceae; *Armeniacae Semen Amarum*] (85 g), *Gypsum Fibrosum* (255 g), *Isatis indigotica* Fort. [Cruciferae; *Isatudus Radix*] (255 g), *Dryopteris crassirhizom*a Nakai [Polypodiaceae; *Dryopteris Crassirhizomatis Rhizoma*] (255 g), *Houttuynia cordata* Thunb. [Saururaceae; *Houttuyniae Botanical drug*] (255 g), *Pogostemon cablin* (Blanco) Benth. [Lamiaceae; *Pogostemonis Botanical drug*] (85 g), *Rheum palmatum* L. [Polygonaceae; *Rhei Radix et Rhizoma*] (51 g), *Rhodiola crenulata* (Hook. f. *& Thoms.*) *H. Ohba* [Crassulaceae; *Rhodiolae Crenulatae Radix et Rhizoma*] (85 g), *Mentha haplocalyx* Briq. [Lamiaceae; *1-Menthol*] (7.5 g), *Glycyrrhiza uralensis* Fisch. ex DC. [Fabaceae; *Glycyrrhizae Radix et Rhizoma*] (85 g) (Rivera et al., 2014; Royal Botanic Gardens, Kew, 2021; Royal Botanic Gardens, Kew science, 2021; The Plant List, 2021; Yiling Pharmaceutical, 2021). These above ingredients are decocted, distilled, filtered, refrigerated, mixed well with powdered sugar, dextrin or starch, dried and finally made into LHQW (Chinese Pharmacopoeia, 2020). Overall, 61 chemical compounds of LHQW (including iridoids, flavonoids, anthraquinones, phenylpropanoids, triterpenoids, and other types) have been unambiguously or tentatively identified via rapid ultra-performance liquid chromatography coupled with diode-array detector and quadrupole time-of-flight mass spectrometry (Jia et al., 2015). Twelve representative compounds were further quantified as chemical markers, including amygdalin, forsythoside E, salidroside, glycyrrhizic acid, chlorogenic acid, hyperin, rutin, forsythoside A, cryptochlorogenic acid, sweroside, phillyrin, and rhein (Jia et al., 2015). In recent years, LHQW has played a significant role in the prevention and control of viral public health events (Yao et al., 2020), and is widely accepted as a representative antiviral Chinese medicine in China.

The COVID-19 pneumonia epidemic has spread across the globe poses a significant challenge to public health worldwide. In the COVID-19 pneumonia epidemic of China, traditional Chinese medicine plays a critical role in preventing mild or ordinary COVID-19 patients from developing into serious or critical patients (China Central Television News, 2020). No proven effective antiviral treatments for COVID-19 pneumonia are available at present (Ahsan et al., 2020; Cao et al., 2020; Chan et al., 2020; Khan et al., 2020; Kupferschmidt and Cohen, 2020). LHQW is recommended by the National Health Commission of the People’s Republic of China as a traditional Chinese medicine appropriate for COVID-19 pneumonia (trial version from Fourth to the Eighth Edition). The China National Medical Products Administration agency has officially approved LHQW for the treatment of mild or ordinary COVID-19 pneumonia (Ahsan et al., 2020; Cao et al., 2020; Chan et al., 2020; Khan et al., 2020; Kupferschmidt and Cohen, 2020). However, while the potential clinical efficacy of LHQW has been demonstrated in several studies (Hu et al., 2020; Wu et al., 2020; Xiao et al., 2020; Wang et al., 2021), no consensus has been reached regarding the adverse reactions associated with LHQW therapy to date. The current meta-analysis was conducted to systematically evaluate the security of LHQW compared with conventional drug in clinical application.

## Materials and Methods

Our meta-analysis was conducted in accordance with the guidelines of the Preferred Reporting Items for Systematic Reviews and Meta-analyses (PRISMA) statement (Moher et al., 2009), and registered with PROSPERO (CRD42020224180).

### Selection Criteria

Electronic retrieval was performed using following databases, which were searched from outset to February 18, 2021: Embase, PubMed, the Cochrane Library, CNKI (a Chinese database), VIP (a Chinese database), and Wanfang (a Chinese database). The following terms were searched using the title, abstract or keywords in the English databases: “Lianhuaqinwen granule,” “Lianhuaqinwen capsule,” “Lianhua Qingwen capsule/granule.” For the Chinese databases, the search terms used were: “连花清瘟颗粒,” “连花清瘟胶囊,” “连花清瘟颗粒/胶囊.”

### Inclusion Criteria

The “participants, interventions, comparison, outcome and study design” (PICOS) criteria were used to identify articles: 1) participants: patients treated with LHQW, 2) interventions: LHQW or LHQW combined with conventional drug (LHQW capsule: Shijiazhuang Yiling Pharmaceutical Co., Ltd., National medicine approval Z20040063; LHQW granule: Shijiazhuang Yiling Pharmaceutical Co., Ltd., National medicine approval Z20100040), 3) comparison: conventional drugs, including antiviral drugs, conventional antibiotics or symptomatic treatment, 4) outcome: relevant data on adverse reactions, and 5) study design: experimental study.

### Exclusion Criteria

Case reports, unrelated studies, those with duplicate data, reviews, commentaries and animal-based studies were excluded from the current meta-analysis.

### Data Extraction and Quality Evaluation

The following information was collected from each qualifying study: first author’s name, year, treating disease, sample size, number of male and female, age, adverse reactions, dispose or outcome of adverse reactions. The term ‘adverse reaction’ represents any undesired and unintended response that occurs with normal drug use (Gallelli et al., 2002). Adverse reaction may include symptoms, abnormal laboratory tests or sign or a cluster of atypical signs (Hallas et al., 1990), it was classified refer to the World Health Organization Adverse Drug Reaction Terminology (2009), for those that could not be identified as “other adverse reactions.” Disagreements were resolved by discussion among all reviewers. The Cochrane Collaboration risk of bias tool comprising seven items was applied to assess the risk of bias of each eligible study (Higgins et al., 2011). The following aspects were assessed for each study: random sequence generation, allocation concealment, blinding, incomplete outcome data, selective outcome reporting, and other sources of bias. Studies were classified as “low risk of bias,” “high risk of bias” or “unclear risk of bias” based on individual items.

### Statistical Analysis

Statistical analysis was performed with Stata 14 software (Stata Corporation, College Station, TX, USA). The risk ratio (RR) and 95% confidence interval (CI) were calculated for categorical variables. Heterogeneity was evaluated using I^2^ statistic. In cases of I^2^ > 50%, obvious heterogeneity was defined and the random-effects model selected. Otherwise, the fixed-effects model was used (DerSimonian and Laird, 1986; Higgins et al., 2003). Subgroup, sensitivity and meta-regression analyses were conducted to determine the potential sources of heterogeneity. Subgroup analysis was performed based on adverse reactions and sensitivity analysis implemented to investigate the influence of each study on the overall evaluation by excluding studies in turn. Potential publication bias was assessed using Funnel plot, Begg’s and Egger’s test. The “trim and fill” method was employed in cases where publication bias existed (Duval and Tweedie, 2000). Data were considered statistically significant at *p* values <0.05.

## Results

### Search Results

**FIGURE 1:**
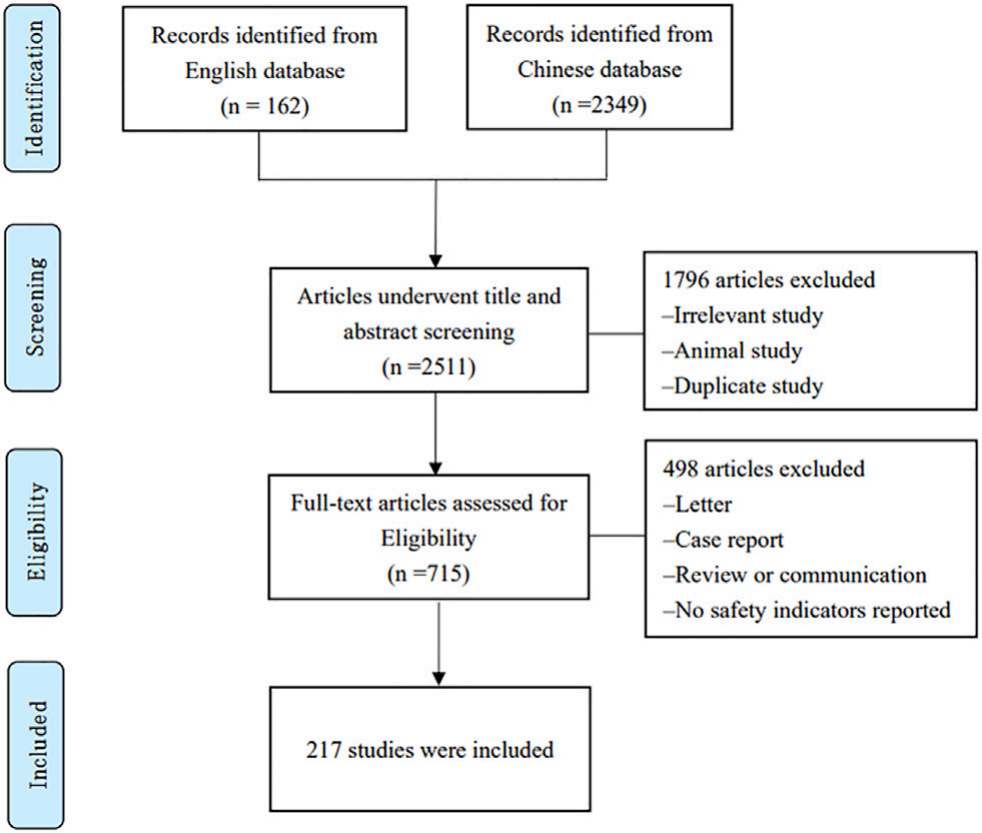
Flow chart of the search and selection process.

A total of 2,511 citations were identified, among which 1,796 irrelevant, animal studies or duplicate studies were excluded, for screen of titles and abstracts. Upon full text filtering of the remaining 715 citations, 498 were excluded due to a number of reasons (shown in Figure 1). Finally, 217 experimental studies on the Chinese population were included for study featuring 1759 cases of adverse reactions following clinical treatment (publication years ranging from 2005 to 2021). Figure 1 provides a detailed flowchart of the screening process. The main characteristics of the eligible studies are summarized in Supplementary Table S1, while the composition of LHQW and how LHQW were reported in the original studies presented in Supplementary Table S2.

### Quality Evaluation

**FIGURE 2:**
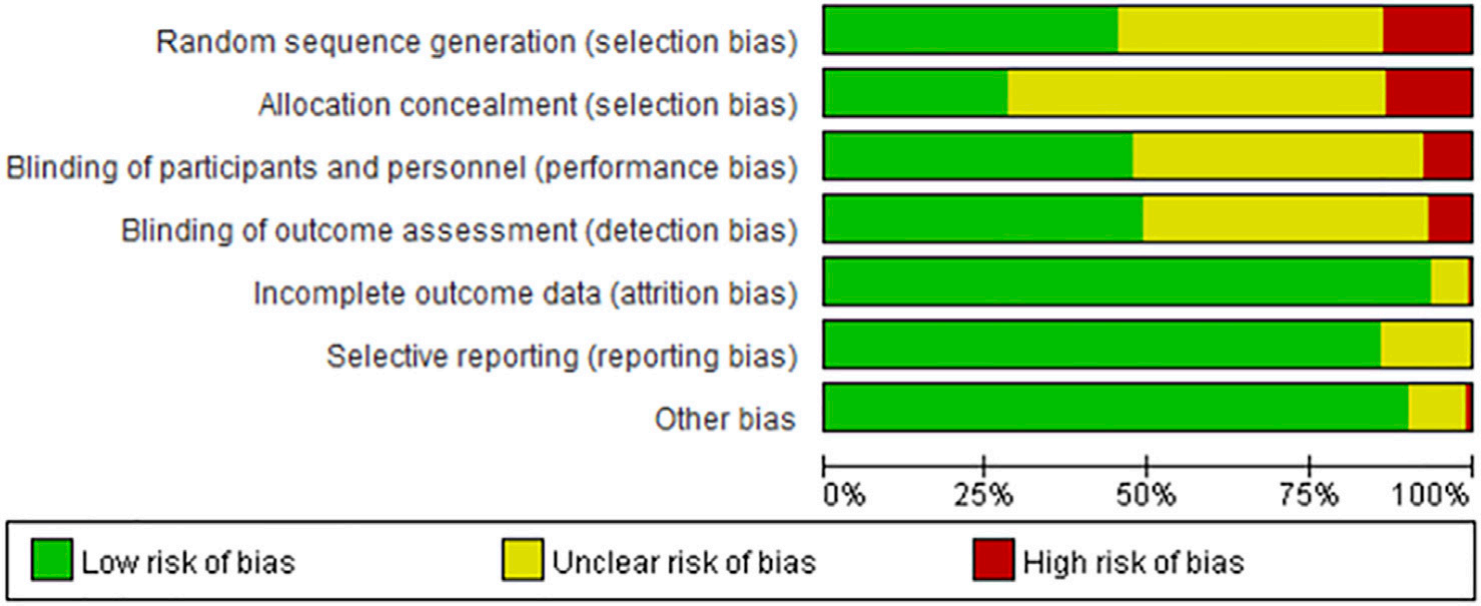
Percentage of risk of bias for all qualified studies.

In total, 95 studies reported randomization data while 93 did not provide information on randomization, and the remaining 29 were grouped according to order of intervention or hospitalization. The majority of studies lacked sufficient description of allocation concealment and therefore, the project was predominantly evaluated as “unclear risk of bias.” Although the blinding method was not reported, since some symptom indicators needed measurement with instruments, assessment of symptom indicators was unlikely to be affected by the lack of blinding. Therefore, studies with blinded participants and personnel and blinded outcome assessments were classified as “low risk of bias.” In terms of incomplete outcome data and selective reporting, the majority of studies reported complete data on all symptom indicators with a low risk of attrition and reporting bias. Homogeneity of baseline data was reported in most studies. Accordingly, other biases were defined as “low risk of bias.” The risk of bias data is presented as a percentage, as shown in Figure 2.

### Quantitative Analyses

For pooled studies, there was a significantly reduced risk of adverse reactions with LHQW group compared to the conventional drug group (RR = 0.63, 95% CI = 0.58–0.69, *p* < 0.001).

### Evaluation of Treating Diseases

#### Influenza A (H1N1)

17 studies focused on the treatment of Influenza A (H1N1), 12 of which reported adverse reactions. The incidence of adverse reactions during treatment of influenza A (H1N1) was lower in the LHQW group compared to the conventional drug group (RR = 0.21, 95% CI = 0.13–0.36, *p* < 0.001).

#### COVID-19 Pneumonia

Five studies reported treatment of COVID-19 pneumonia, with two documenting adverse reactions. There was no statistically significant difference in the incidence of adverse reactions during treatment of COVID-19 pneumonia between the LHQW group and the conventional drug group (RR = 0.51, 95% CI = 0.14–1.82, *p* = 0.297).

#### Chronic Obstructive Pulmonary Disease

Seven studies reporting treatment of chronic obstructive pulmonary disease were included, among which two described adverse reactions. There was no statistically significant difference in the incidence of adverse reactions during treatment of chronic obstructive pulmonary disease between the LHQW group and the conventional drug group (RR = 1.27, 95% CI = 0.51–3.17, *p* = 0.608).

#### Respiratory Tract Infection

43 studies on treatment of respiratory tract infection were examined, with 29 reporting adverse reactions. There was no statistically significant difference in the incidence of adverse reactions during treatment of respiratory tract infection between the LHQW group and the conventional drug group (RR = 0.78, 95% CI = 0.58–1.03, *p* = 0.083).

#### Hand-Foot- Mouth Disease

Among the 13 included studies on treatment of hand-foot-mouth disease, eight reported adverse reactions. There was no statistically significant difference in the incidence of adverse reactions during treatment of hand-foot-mouth disease between the LHQW group and the conventional drug group (RR = 0.53, 95% CI = 0.22–1.30, *p* = 0.165).

#### Common Pneumonia

Twenty-six studies focused on treatment of common pneumonia, of which 23 reported adverse reactions. There was no statistically significant difference in the incidence of adverse reactions during treatment of common pneumonia between the LHQW group and the conventional drug group (RR = 0.99, 95% CI = 0.79–1.24, *p* = 0.927).

#### Influenza

We examined 54 studies on treatment of influenza, 42 of which reported adverse reactions. In this analysis, the incidence of adverse reactions during treatment of influenza was lower in the LHQW group compared to the conventional drug group (RR = 0.74, 95% CI = 0.63–0.87, *p* < 0.001).

#### Viral Influenza

Eleven studies reported the treatment of viral influenza and adverse reactions. There was no statistically significant difference in the incidence of adverse reactions during treatment of viral influenza between the LHQW group and the conventional drug group (RR = 0.75, 95% CI = 0.47–1.21, *p* = 0.244).

#### Rheum

Among the eight included studies on rheum therapy, six reported adverse reactions. There was no statistically significant difference in the incidence of adverse reactions during treatment of rheum between the LHQW group and the conventional drug group (RR = 0.46, 95% CI = 0.12–1.83, *p* = 0.270).

#### Herpes Zoster

Six studies were included on treatment of herpes zoster and reported the adverse reactions. There was no statistically significant difference in the incidence of adverse reactions during treatment of herpes zoster between the LHQW group and the conventional drug group (RR = 0.80, 95% CI = 0.52–1.23, *p* = 0.302).

#### Other Diseases

Twenty-three studies on other diseases were included, with 12 reporting adverse reactions. There was no statistically significant difference in the incidence of adverse reactions during treatment of other diseases between the LHQW group and the conventional drug group (RR = 0.95, 95% CI = 0.65–1.39, *p* = 0.780).

### Evaluation of Security Index

#### Abnormal White Blood Cells and Reticuloendothelial System

Six studies reported the adverse reactions of abnormal white blood cells and reticuloendothelial system. There was no statistically significant difference in the incidence of abnormal white blood cells and reticuloendothelial system between the LHQW group and the conventional drug group (RR = 1.00, 95% CI = 0.38–2.62, *p* = 1.000).

#### Hepatobiliary System Damage

Seven studies reported the adverse reactions of hepatobiliary system damage. There was no statistically significant difference in the incidence of hepatobiliary system damage between the LHQW group and the conventional drug group (RR = 0.86, 95% CI = 0.58–1.28, *p* = 0.461). Similar results were obtained in subgroup analysis of hepatic function abnormal and transaminase increased.

#### Respiratory System Damage

Five studies reported respiratory system damage as an adverse event. LHQW group has a reduced incidence of respiratory system damage compared to the conventional drug group (RR = 0.46, 95% CI = 0.29–0.74, *p* = 0.001).

#### Skin and Its Appendage Damage

37 studies reported adverse reactions of skin and its appendage damage. There was a reduced risk of skin and its appendage damage in the LHQW group compared to the conventional drug group (RR = 0.63, 95% CI = 0.44–0.92, *p* = 0.015). Subgroup analysis revealed that LHQW group has a reduced incidence of rash than the conventional drug group (RR = 0.58, 95% CI = 0.39–0.86, *p* = 0.007), no statistical difference was detected for itchy skin.

#### Nervous System Damage

Nervous system damage was described as an adverse event in 34 studies. The incidence of nervous system damage was lower in the LHQW group compared to the conventional drug group (RR = 0.24, 95% CI = 0.18–0.32, *p* < 0.001). Subgroup analysis showed the incidence of dizziness or headache was significantly reduced with LHQW group (RR = 0.54, 95% CI = 0.34–0.84, *p* = 0.006), no statistical difference was detected for drowsiness and legacy neuralgia.

#### Psychiatric Disorders

Two studies reported psychiatric disorders as the adverse reaction. There was no statistically significant difference in the incidence of psychiatric disorders between the LHQW group and the conventional drug group (RR = 1.00, 95% CI = 0.25–3.97, *p* = 1.000).

#### Gastrointestinal System Damage

132 studies documented gastrointestinal system damage. The LHQW group has a reduced incidence of gastrointestinal system damage than the conventional drug group (RR = 0.83, 95% CI = 0.74–0.93, *p* = 0.002). Subgroup analysis showed a lower level of nausea or vomiting in LHQW group (RR = 0.60, 95% CI = 0.48–0.74, *p* < 0.001), no statistical difference was detected for other symptoms of gastrointestinal system damage.

#### Heart Rate and Arrhythmia

Five studies reported heart rate and arrhythmia as adverse events. There was no statistically significant difference in the incidence of heart rate and arrhythmia between the LHQW group and the conventional drug group (RR = 0.67, 95% CI = 0.23–1.93, *p* = 0.454).

#### Body as a Whole-General Disorders

Four studies reported body as a whole-general disorders as an adverse event. There was no statistically significant difference in the incidence of body as a whole-general disorders between the LHQW group and the conventional drug group (RR = 0.77, 95% CI = 0.19–3.01, *p* = 0.708). No statistical difference was detected for sleepy.

#### Other Adverse Reactions

20 studies documented other adverse reactions. LHQW has a reduced incidence of other adverse reactions compared to the conventional drug group (RR = 0.60, 95% CI = 0.43–0.84, *p* = 0.003). Subgroup analysis further showed the incidence of disease recurrence was lower in the LHQW group than the conventional drug group (RR = 0.33, 95% CI = 0.17–0.65, *p* = 0.001), no statistical difference was detected for secondary infection.

### Dispose or Outcome of Adverse Reactions

38 studies reported dispose or outcomes of adverse reactions. Seven of the studies showed that adverse reactions improved or healed spontaneously without any treatment, five reported that adverse reactions healed spontaneously after discontinuation of medication, eight showed that adverse reactions improved or healed with medication after meals and ten showed that adverse reactions improved or recovered after symptomatic treatment, while in the remaining eight studies, adverse reactions were not disposed or treated.

The results of quantitative analyses are presented in Table 1 and the statistical significance of Forest plots shown in Supplementary Figure S1.

**TABLE 1:** The quantitative analysis of all eligible studies.

Subject	RR	95% CI	*P*_*RR*_	Heterogeneity		M
				*I* ^*2*^	*p*	
Pooled studies	0.63	0.58–0.69	<0.001	43.7	<0.001	F
Evaluation of treating disease						
Influenza A (H1N1)	0.21	0.13–0.36	<0.001	0.0	0.998	F
COVID-19 pneumonia	0.51	0.14–1.82	0.297	76.0	0.041	R
Chronic obstructive pulmonary disease	1.27	0.51–3.17	0.608	0.0	0.748	F
Respiratory tract infection	0.78	0.58–1.03	0.083	0.0	0.804	F
Hand-foot- mouth disease	0.53	0.22–1.30	0.165	62.1	0.010	R
Common pneumonia	0.99	0.79–1.24	0.927	48.1	0.006	F
Influenza	0.74	0.63–0.87	<0.001	26.3	0.063	F
Viral influenza	0.75	0.47–1.21	0.244	0.0	0.512	F
Rheum	0.46	0.12–1.83	0.270	88.2	<0.001	R
Herpes zoster	0.80	0.52–1.23	0.302	0.0	0.410	F
Other diseases	0.95	0.65–1.39	0.780	0.0	0.517	F
Evaluation of security index						
Abnormal white blood cells and reticuloendothelial system	1.00	0.38–2.62	1.000	0.0	0.903	F
Hepatobiliary system damage	0.86	0.58–1.28	0.461	0.0	0.893	F
*Hepatic function abnormal*	0.97	0.63–1.45	0.889	0.0	0.501	F
*Transaminase increased*	0.33	0.07–1.63	0.175	0.0	1.000	F
Respiratory system damage	0.46	0.29–0.74	0.001	0.0	0.568	F
Skin and its appendage damage	0.63	0.44–0.92	0.015	0.0	0.999	F
*Rash*	0.58	0.39–0.86	0.007	0.0	1.000	F
*Itchy skin*	1.16	0.51–4.74	0.435	7.3	0.356	F
Nervous system damage	0.24	0.18–0.32	<0.001	49.2	0.001	F
*Drowsiness*	0.32	0.01–77.01	0.687	91.8	<0.001	R
*Legacy neuralgia*	0.73	0.41–1.31	0.288	0.0	0.424	F
*Dizziness or headache*	0.54	0.34–0.84	0.006	0.0	0.770	F
Psychiatric disorders	1.00	0.25–3.97	1.000	0.0	0.400	F
Gastrointestinal system damage	0.83	0.74–0.93	0.002	0.0	0.922	F
*Diarrhea/Abdominal distension/Abdominal pain/Abdominal discomfort*	0.87	0.72–1.06	0.172	0.0	0.729	F
*Nausea or vomiting*	0.60	0.48–0.74	<0.001	0.0	0.984	F
*Gastrointestinal symptoms or dyspepsia*	0.57	0.31–1.07	0.079	30.8	0.205	F
*Gastrointestinal distress*	1.17	0.91–1.50	0.230	0.0	0.722	F
*Dry mouth or loss of appetite*	0.81	0.47–1.41	0.456	0.0	0.707	F
Heart rate and arrhythmia	0.67	0.23–1.93	0.454	0.0	0.600	F
Body as a whole-general disorders	0.77	0.19–3.01	0.708	0.0	0.948	F
*Sleepy*	0.98	0.20–4.78	0.983	0.0	1.000	F
Other adverse reactions	0.60	0.43–0.84	0.003	0.0	0.952	F
*Resurgence of disease*	0.33	0.17–0.65	0.001	0.0	0.750	F
*Secondary infection*	0.74	0.39–1.42	0.369	0.0	0.798	F

RR, rate ratio, M, model, R, random-effects model; F, fixed-effects model.

### Publication Bias

Begg’s test and Egger’s test detected no visible publication bias for data from pooled studies and disease evaluation. However, in evaluation of security index, Egger’s test and funnel plot analyses disclosed the existence of publication bias with regard to hepatobiliary system damage, psychiatric disorders, gastrointestinal system damage and body as a whole-general disorders. The “Trim and Fill” method was further applied to assess the impact of publication bias. The results remained statistically robust after different number supplement of potential studies (for hepatobiliary system damage: RR = 0.90, 95% CI = 0.60 to 1.34; for psychiatric disorders: RR = 1.00, 95% CI = 0.20 to 4.91; for gastrointestinal system damage: RR = 0.86, 95% CI = 0.76 to 0.98; for body as a whole-general disorders: RR = 0.73, 95% CI = 0.21–2.56).

**FIGURE 3:**
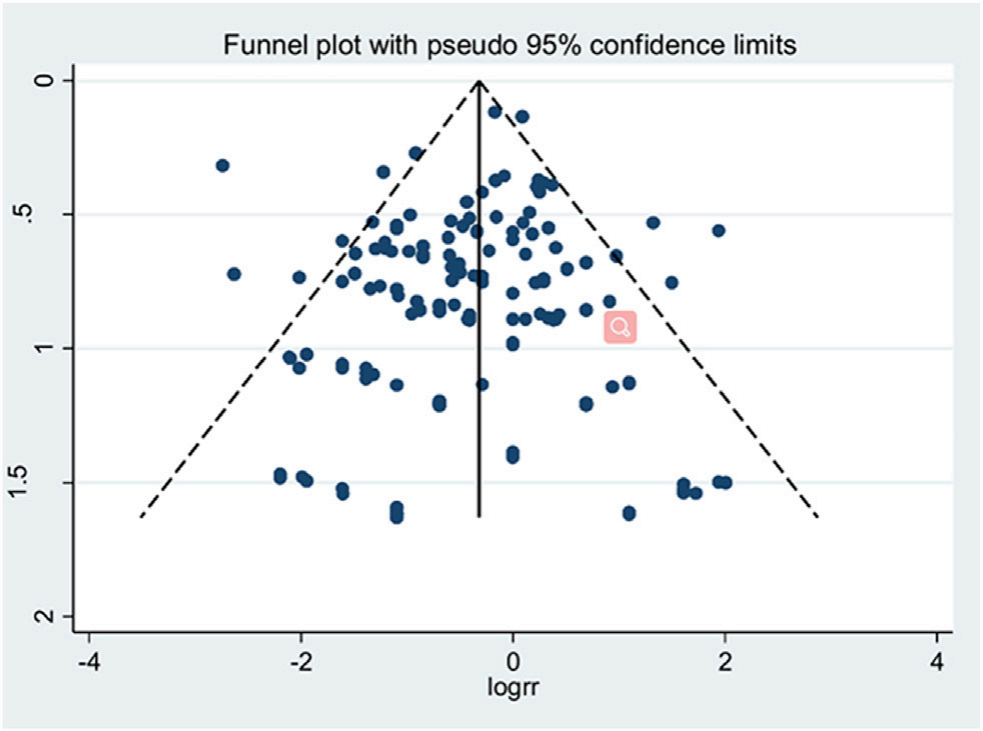
Funnel plot of pooled studies.

Publication bias data are shown in Table 2 and the Funnel plot of pooled studies presented in Figure 3.

**TABLE 2:** Results of publication bias and sensitivity analysis.

Subject	Publication bias		Sensitivity analysis	
	*P*_*begg*_	*P*_*egger*_	RR	95% CI
Pooled studies	0.738	0.297	0.63	0.54–0.80
Evaluation of treating diseases				
Influenza A (H1N1)	0.645	0.528	0.21	0.11–0.39
COVID-19 pneumonia	—	—	—	—
Chronic obstructive pulmonary disease	—	—	—	—
Respiratory tract infection	0.613	0.362	0.72	0.51–1.01
Hand-foot- mouth disease	0.386	0.323	0.53	0.17–1.70
Common pneumonia	0.958	0.615	0.99	0.67–1.34
Influenza	0.363	0.280	0.74	0.50–0.94
Viral influenza	1.000	0.898	0.75	0.39–1.55
Rheum	1.000	0.339	0.46	0.08–2.83
Herpes zoster	0.624	0.669	0.80	0.38–1.41
Other diseases	0.193	0.492	0.95	0.55–1.62
Evaluation of security index				
Abnormal white blood cells and reticuloendothelial system	1.000	0.995	1.00	0.29–3.41
Hepatobiliary system damage	0.548	0.010	0.86	0.18–1.35
Respiratory system damage	1.000	0.999	0.46	0.24–0.98
Skin and its appendage damage	0.708	0.355	0.63	0.41–0.96
Nervous system damage	0.744	0.390	0.24	0.16–0.83
Psychiatric disorders	1.000	0.010	1.00	0.04–7.04
Gastrointestinal system damage	0.321	0.026	0.83	0.70–0.95
Heart rate and arrhythmia	0.806	0.741	0.67	0.14–3.91
Body as a whole-general disorders	0.734	<0.001	0.77	0.14–3.06
Other adverse reactions	0.657	0.954	0.60	0.40–0.92

### Sensitivity Analysis and Meta-regression

Sensitivity analysis was performed by assessing the stability of the results after removal of individual studies. As adverse reactions in COVID-19 pneumonia and chronic obstructive pulmonary disease treatment were only reported for a limited number of studies, no sensitivity analysis for these diseases was conducted. We observed no significant alteration in the security evaluation indexes of treatment among different diseases. The sensitivity results are shown in Table 2.

Significant heterogeneity was detected for COVID-19 pneumonia (I^2^ = 76.0), hand-foot-mouth disease (I^2^ = 62.1), rheum (I^2^ = 88.2), and drowsiness of nervous system damage (I^2^ = 91.8). However, since subgroup and sensitivity analyses did not explore the source of heterogeneity, we utilized meta-regression to determine the potential underlying factors. No meta-regression was performed for COVID-19 pneumonia due to the limited number of studies. For hand-foot-mouth disease, sample size (*p* = 0.082), year of publication (*p* = 0.795), and treatment of the control group (*p* = 0.701) did not explain the issue of heterogeneity. For rheum, sample size could partially explain heterogeneity (*p* = 0.005), but not year of publication (*p* = 0.658) and treatment of the control group (*p* = 0.712). For drowsiness of nervous system damage, sample size (*p* = 0.500), year of publication (*p* = 0.157), and treatment of the control group (*p* = 0.599) were not linked with heterogeneity. The inconsistent use of conventional drugs in individual studies, along with individual differences in drug absorption, may be factors underlying high heterogeneity.

## Discussion

Traditional Chinese medicine has accumulated substantial clinical experience over thousands of years. Traditional Chinese medicine treatment maintains homeostasis through interactions between the body and pathogens or establishing a balance between stimulating antiviral responses and suppressing overactive immune responses that leads to tissue damage (Shi et al., 2021). LHQW, an innovative proprietary traditional Chinese medicine used to treat influenza, was approved by the Food and Drug Administration of the United States for Phase II of clinical trial in 2015 (Ye et al., 2020). In recent years, LHQW has been registered to obtain marketing authorization in Canada, Brazil, Singapore, Thailand, the Philippines, Kuwait and other countries under the designation “Chinese patent medicine,” “medicine,” “physical natural health products,” “food supplement,” “modern botanical medicine” or “natural medicine” (Yiling Pharmaceutical, 2020). Previous studies reported that Lianhua Qingwen might have positive effects, including broad-spectrum antiviral, cough and expectorant, antipyretic and anti-inflammatory, immune-regulating, effective antibacterial and other systemic intervention functions (Liu et al., 2010; Liu 2015; Wang, et al., 2015). Experiments on animals using a combination of LHQW and oseltamivir to treat influenza B virus infections showed a potential inhibitory effect on overexpression of TNF-α, MIP-1β, RANTES, IFN-λ, IL-6, IL-8, IP-10, and MCP-1 at the mRNA level, and consequent prevented of the development of severe inflammatory reactions (Yang et al., 2020). Considerable research to date has focused on the clinical efficacy of LHQW, the safety of LHQW is additionally an important consideration for clinical application that should not be neglected.

Here, we performed a meta-analysis of 217 Chinese experimental studies to evaluate the safety of LHQW. In the evaluation of treating disease, the incidence of adverse reactions during treatment of influenza A (H1N1) and influenza were lower in the LHQW group compared to the conventional drug group. A recent meta-analysis came from Zhang and Liu, (2014), focused on treatment for influenza A (H1N1) with LHQW in conjunction with oseltamivir. No adverse treatment-related effects were reported in all the included publications (from 2009 to 2011). While LHQW has recently been approved for marketing by CFDA, its safety is yet to be conclusively established. With gradual improvement of the adverse drug reaction monitoring system of China, reports of adverse reactions or events have increased. In our meta-analysis, information on adverse reactions was clearly reported in 12 of the 17 studies while the remaining five studies provided no specific evidence of adverse reaction specifically, and the incidence of adverse reactions was lower in LHQW group than that in the conventional drug group. Wu et al. (2020) identified 38 randomized controlled trials (RCTs) comparing LHQW with antivirals or other Chinese medicines for treating influenza and reported a lower risk of adverse reactions in the LHQW group compared to oseltamivir (RR = 0.29, 95%CI = 0.11–0.80), and ribavirin groups (RR = 0.29, 95%CI = 0.11–0.80). Although the results obtained for influenza were consistent with our findings, the control group in this earlier study was treated with a single drug while our control groups were treated with antiviral drugs, conventional antibiotics or symptomatic therapy. Comprehensive analysis of influenza may provide a significant advantage in generalization of the conclusions. A study by Lei (2020) on childhood influenza treatment revealed no statistical difference of adverse effects between LHQW group and conventional drug group (*p* = 0.751), inconsistent with our meta-analysis. The different incidence of these adverse reactions may be related to the metabolic level, which varies greatly among the different age groups. Therefore, further attention should be paid to the occurrence of adverse reaction with LHQW among different age groups in the future. LHQW has been recommended by the ‘China International Exchange and Promotive Association for Medical and Health Care’ and the “Chinese Research Hospital Association” as comprehensive treatment for moderate or chronic COVID-19 pneumonia in conjunction with routine therapy (Jin et al., 2020). The latest meta-analysis by Fan et al. (2021) investigated the efficacy and security of LHQW in the treatment of mild or moderate COVID-2019 pneumonia, reported no statistical difference in the incidence of adverse reactions between LHQW and conventional treatment groups from two studies (RR = 0.43, 95%CI = 0.12–1.54, *p* = 0.19). Few reports of adverse reactions in the course of treating COVID-19 pneumonia with LHQW are available. Data from our meta-analysis also failed to compare the security of LHQW with conventional drugs for treatment of COVID-19 pneumonia, which requires further investigation.

In the evaluation of security indexes, our meta-analysis revealed that LHQW group having a lower level of adverse reactions compared to the conventional drug group, such as respiratory system damage, skin and its appendage damage, nervous system damage, gastrointestinal system damage and other adverse reactions. Subgroup analysis additionally demonstrated LHQW group has a reduced incidence of rash, nausea or vomiting, and resurgence of disease. Compared with the conventional drug group, the incidence of disease recurrence was lower in the LHQW group, implied that LHQW might could regulate and enhance the immune function of the body. Based on analysis of 40 cases of RCT, the clinical safety of LHQW was comprehensively examined, 163 adverse reactions were reported from 24 studies (Wang et al., 2013). The group reported significantly lower incidence of adverse reactions (RR = 0.62, 95% CI = 0.46–0.82) and lower incidence of gastrointestinal system damage in the LHQW group (RR = 0.65, 95% CI = 0.46–0.92) relative to the control group. However, no statistical difference was detected with regard to rash (RR = 0.60, 95% CI = 0.21–1.67) and dizziness (RR = 1.79, 95% CI = 0.33–9.74). These results were different from our findings, which may be attributed to the limited number of adverse reactions reported in the previous study.

Some composition of LHQW can cause damage to the stomach and intestines, supporting the possibility of injurious effects on the gastrointestinal system: *for thia suspensa* (Thunb.) Vahl [Oleaceae; *Forsythiae Fructus*], *Lonicera japonica* Thunb. [Caprifoliaceae; *Lonicerae Japonicae Flos*], *Gypsum Fibrosum*, *Isatis indigotica* Fort. [Cruciferae; *Isatudus Radix*], *Dryopteris crassirhizoma* Nakai [Polypodiaceae; *Dryopteris Crassirhizomatis Rhizoma*], *Houttuynia cordata* Thunb. [Saururaceae; *Houttuyniae Botanical drug*], *Rheum palmatum* L. [Polygonaceae; *Rhei Radix et Rhizoma*] (Peng et al., 2015). Skin rashes, allergic dermatitis and other skin allergic reactions are also reported to be associated with LHQW treatment, and may be related to *Houttuynia cordata* Thunb. [Saururaceae; *Houttuyniae Botanical drug*], a composition of LHQW (Huang et al., 2021), that contains allergenic ingredients, such as chlorogenic acid, as well as the unstable nature of houttuyfonate, which can cause adverse reactions (Cao, 2016). On March 27, 2019, Yiling Pharmaceutical Co. Ltd. amended the LHQW drug manual to include adverse reactions related to its clinical application. The post-marketing monitoring data incorporated information on the following gastrointestinal adverse reactions: nausea, diarrhea, vomiting, abdominal pain, abdominal distension, dry mouth; as well as skin rash, itching, dizziness (Yiling Pharmaceutical, 2021). Our findings support the adverse reactions recorded by Yiling Pharmaceutical, and compare the incidence risk of gastrointestinal system damage and rash with the control group. Furthermore, we also found that LHQW group has a reduced incidence of respiratory system damage compared to the conventional drug group. The clinical application experience of LHQW has improved over the years, the clinical considers the patient’s condition, physical quality, and age, controls the amount of LHQW prescribed accordingly and thereby, reduced the likelihood of adverse reactions.

Management of adverse reactions was clearly documented in 38 studies, which reported improvement or resolution through discontinuation of medication, changes to post-meal administration, symptomatic or no treatment. The most common adverse reactions in our analysis were gastrointestinal system damage, skin and its appendage damage. Since the adverse reactions of LHQW are relatively minor, these functional changes can be recovered. Notably, the stomach is full after a meal, and LHQW does not directly contact the gastric mucosa, which reduces the risk of irritation of stomach mucous membrane and thus the occurrence of side-effects.

To protect the interests of patients, the risk-benefit ratio of the drug needs to be considered in clinical applications. While the results from our study showed that LHQW has a potentially reduced incidence of adverse reactions in certain diseases and symptoms to a greater extent than conventional treatment, efficacy is an important consideration that should not be overlooked.

Several limitations of our meta-analysis should be taken into consideration. First, the quality of qualified studies was generally medium, randomization procedures and blinding information were lacking in most studies. Second, one purposes of this study was to retrieve information on adverse reactions from different countries, due to language barriers, only English and Chinese articles were retrieved, and adverse reactions in the Chinese population were specifically analyzed, the effects on populations from other countries therefore require further investigation. Finally, all adverse reactions were described solely based on grouping of patients into treatment and control categories and no data were obtained according to gender stratification, which limited further analysis.

A number of advantages of our study should additionally be mentioned. Firstly, to our knowledge, this meta-analysis provides the most comprehensive evaluation of the clinical safety of LHQW. Secondly, separate evaluations were further performed for different diseases and types of adverse reactions. Finally, our data showed that LHQW reduces the incidence of a number of adverse reactions compared with conventional drugs.

## Conclusion

This study provides a basis for establishing the clinical safety profile of LHQW. High-quality randomized controlled trials conducted over the long-term from multiple countries are warranted to further validate the efficacy and safety of LHQW.

## Data Availability

The original contributions presented in the study are included in the article/Supplementary Material, further inquiries can be directed to the corresponding author.

## References

[B1] Ahsan W. Javed S. Bratty M. A. Alhazmi H. A. Najmi A. (2020). Treatment of SARS-Cov-2: How Far Have We Reached? Drug Discov. Ther. 14 (2), 67–72. 10.5582/ddt.2020.03008 32336723

[B2] Cao B. Zhang D. Wang C. (2020). A Trial of Lopinavir-Ritonavir in Covid-19. Reply. N. Engl. J. Med. 382 (21), e68. 10.1056/NEJMc2008043 32369286

[B3] Cao L. J. (2016). Analysis of Clinical Application and Adverse Reactions of Houttuynia Cordata Injection. Asia-Pacific Traditional Med. 12 (08), 140–141. 10.11954/ytctyy.201608076

[B4] Chan K. W. Wong V. T. Tang S. C. W. (2020). Covid-19: an Update on the Epidemiological, Clinical, Preventive and Therapeutic Evidence and Guidelines of Integrative Chinese-Western Medicine for the Management of 2019 Novel Coronavirus Disease. Am. J. Chin. Med. 48 (3), 737–762. 10.1142/S0192415X20500378 32164424

[B5] China Central Television News (2020). National Administration of Traditional Chinese Medicine: Traditional Chinese Medicine Is Involved in a Wide Range of Treatment and Early Intervention Has Good Effects. Available at: http://m.news.cctv.com/2020/03/17/ARTIEYQ2aws8fPYWQxjnynzo200317.shtml?from=singlemessage (Accessed Apirl 29, 2021).

[B6] Chinese Pharmacopoeia (2020). Pharmacopoeia of People’s Republic of China. Beijing: China Medical Science Press.

[B7] DerSimonian R. Laird N. (1986). Meta-analysis in Clinical Trials. Control. Clin. Trials. 7 (3), 177–188. 10.1016/0197-2456(86)90046-2 3802833

[B8] Duval S. Tweedie R. (2000). Trim and Fill: a Simple Funnel-Plot-Based Method of Testing and Adjusting for Publication Bias in Meta-Analysis. Biometrics. 56 (2), 455–463. 10.1111/j.0006-341x.2000.00455.x 10877304

[B9] Fan Z. Guo G. Che X. Yang Y. Liu Y. Li L. (2021). Efficacy and Safety of Lianhuaqingwen for Mild or Moderate Coronavirus Disease 2019. Medicine (Baltimore). 100 (21), e26059. 10.1097/MD.0000000000026059 34032734 PMC8154466

[B10] Gallelli L. Ferreri G. Colosimo M. Pirritano D. Guadagnino L. Pelaia G. (2002). Adverse Drug Reactions to Antibiotics Observed in Two Pulmonology Divisions of Catanzaro, Italy: a Six-Year Retrospective Study. Pharmacol. Res. 46 (5), 395–400. 10.1016/s1043661802002104 12419643

[B11] Hallas J. Harvald B. Gram L. F. Grodum E. Brøsen K. Haghfelt T. (1990). Drug Related Hospital Admissions: the Role of Definitions and Intensity of Data Collection, and the Possibility of Prevention. J. Intern. Med. 228 (2), 83–90. 10.1111/j.1365-2796.1990.tb00199.x 2394974

[B12] Higgins J. P. Altman D. G. Gøtzsche P. C. Jüni P. Moher D. Oxman A. D. (2011). The cochrane Collaboration's Tool for Assessing Risk of Bias in Randomised Trials. BMJ. 343, d5928. 10.1136/bmj.d5928 22008217 PMC3196245

[B13] Higgins J. P. Thompson S. G. Deeks J. J. Altman D. G. (2003). Measuring Inconsistency in Meta-Analyses. BMJ. 327 (7414), 557–560. 10.1136/bmj.327.7414.557 12958120 PMC192859

[B14] Hu C. Liang M. Gong F. He B. Zhao D. Zhang G. (2020). Efficacy of Lianhua Qingwen Compared With Conventional Drugs in the Treatment of Common Pneumonia and Covid-19 Pneumonia: a Meta-Analysis. Evid. Based Complement. Alternat Med. 2020, 5157089. 10.1155/2020/5157089 32963563 PMC7501551

[B15] Huang N. L. Huang H. M. Zhang B. Y. Xie J. (2021). Pharmacological Action, Clinical Application and Adverse Reactions of Houttuynia Cordata. Fujian J. Traditional Chin. Med. 52 (3), 58–60. 10.13260/j.cnki.jfjtcm.012210

[B16] Jia W. Wang C. Wang Y. Pan G. Jiang M. Li Z. (2015). Qualitative and Quantitative Analysis of the Major Constituents in Chinese Medical Preparation Lianhua-Qingwen Capsule by UPLC-DAD-QTOF-MS. ScientificWorldJournal. 2015, 731765. 10.1155/2015/731765 25654135 PMC4308632

[B17] Jin L. Xu Y. Yuan H. (2020). Effects of Four Types of Integrated Chinese and Western Medicines for the Treatment of Covid-19 in china: a Network Meta-Analysis. Rev. Assoc. Med. Bras (1992). 66 (6), 771–777. 10.1590/1806-9282.66.6.771 32696884

[B18] Khan Z. Karataş Y. Rahman H. (2020). Anti Covid-19 Drugs: Need for More Clinical Evidence and Global Action. Adv. Ther. 37 (6), 2575–2579. 10.1007/s12325-020-01351-9 32350686 PMC7189176

[B19] Kupferschmidt K. Cohen J. (2020). Race to Find Covid-19 Treatments Accelerates. Science. 367 (6485), 1412–1413. 10.1126/science.367.6485.1412 32217705

[B20] Lei X. (2020). Efficacy and Safety of Lianhua Qingwen Granule Combined with Oseltamivir in the Treatment of Influenza Virus Infection in Children. Drugs and Clinic. 17 (13), 62–64. 10.3969/j.issn.1672-2809.2020.13.020

[B21] Liu C. Y. Li X. Q. Cai S. Q. (2010). Advances in Pharmacology and Clinical Research of Lianhua Qingwen Capsules. Pharmacol. Clin. Chin. Materia Med. 6 (26), 84–85. 10.13412/j.cnki.zyyl.2010.06.026

[B22] Liu X. Y. (2015). Preliminary Study on the Inhibitory Effect of Lianhua Qingwen Capsule on virusMaster Degree. Kunming: Kunming University of technology.

[B23] Moher D. Liberati A. Tetzlaff J. Altman D. G. PRISMA Group (2009). Preferred Reporting Items for Systematic Reviews and Meta-Analyses: the PRISMA Statement. BMJ. 339 (7), b2535. 10.1371/journal.pmed.100009710.1136/bmj.b2535 19622551 PMC2714657

[B24] Peng L. L. Li L. Shen L. Li X. X. (2015). Literature Analysis of Clinical Application and Adverse Drug Reaction/Event of Lianhua Qingwen Capsule. Chin. J. Pharmacovigilance. 12 (12), 753759–754755. 10.19803/j.1672-8629.2015.12.011

[B25] Rivera D. Allkin R. Obón C. Alcaraz F. Verpoorte R. Heinrich M. (2014). What Is in a Name? the Need for Accurate Scientific Nomenclature for Plants. J. Ethnopharmacol. 152 (3), 393–402. 10.1016/j.jep.2013.12.022 24374235

[B26] Royal Botanic Gardens, Kew (2021). Plants of the World Online. Available at: http://www.plantsoftheworldonline.org (Accessed December 18, 2021).

[B27] Royal Botanic Gardens, Kew science (2021). Medicinal Plant Names Services. Available at: https://mpns.science.kew.org/mpns-portal/ (Accessed December 19, 2021).

[B28] Shi M. Peng B. Li A. Li Z. Song P. Li J. (2021). Broad Anti-viral Capacities of Lian-Hua-Qing-Wen Capsule and Jin-Hua-Qing-gan Granule and Rational Use against Covid-19 Based on Literature Mining. Front. Pharmacol. 12, 640782. 10.3389/fphar.2021.640782 34054522 PMC8160462

[B29] The Plant List (2021). A Working List of All Plant Species. Available at: http://www.theplantlist.org/ (Accessed December 18, 2021).

[B30] Wang Y. Greenhalgh T. Wardle J. (2021). Chinese Herbal Medicine ("3 Medicines and 3 Formulations") for COVID ‐19: Rapid Systematic Review and Meta‐analysisChinese Herbal Medicine ("3 Medicines and 3 Formulations") for COVID-19: Rapid Systematic Review and Meta-Analysis. J. Eval. Clin. Pract. 28 (1), 13–32. 10.1111/jep.13614 34528735 PMC8657519

[B31] Wang Y. X. Zhang K. Y. Huang J. H. Han S. L. Zhao J. H. Xu Z. J. (2013). Clinical Drug Safety of Lianhuaqingwen Preparation: a Systematic Evaluation. Eval. Anal. Drug-Use Hospitals China. 13 (08), 676–681. 10.14009/j.issn.1672-2124.2013.08.001

[B32] Wang Y. Z. Wang H. T. Han X. Liu C. Sun Y. N. Liu B. (2015). Inhibitorv Effect of Lianhua Oingwen Water Extract on Methicillin-Resistant staphylococcus Aureus Biofilm *In Vitro* . Chin. J. Nosocomiology. 25 (4), 727790–728729. 10.11816/cn.ni.2015-134443

[B33] World Health Organization Adverse Drug Reaction Terminology (2009). System-Organ Code Retrieval Involved. Chin. J. Pharmacovigilance. 6 (01), 63–64.

[B34] Wu L. Chen Y. Ma Y. Yang Z. Yang N. Deng W. (2020). Clinical Practice Guideline on Treating Influenza in Adult Patients with Chinese Patent Medicines. Pharmacol. Res. 160, 105101. 10.1016/j.phrs.2020.105101 32739428

[B35] Xiao M. Tian J. Zhou Y. Xu X. Min X. Lv Y. (2020). Efficacy of Huoxiang Zhengqi Dropping Pills and Lianhua Qingwen Granules in Treatment of Covid-19: a Randomized Controlled Trial. Pharmacol. Res. 161, 105126. 10.1016/j.phrs.2020.105126 32781283 PMC7414728

[B36] Yang C. Wang Y. He J. Yan W. Jiang H. Chen Q. (2020). Lianhua-qingwen Displays Antiviral and Anti-inflammatory Activity and Synergistic Effects with Oseltamivir against Influenza B Virus Infection in the Mouse Model. Evid. Based Complement. Alternat Med. 2020, 3196375. 10.1155/2020/3196375 32565852 PMC7293728

[B37] Yao K. T. Liu M. Y. Li X. Huang J. H. Cai H. B. (2020). Retrospective Clinical Analysis on Treatment of Novel Coronavirus-Infected Pneumonia with Traditional Chinese Medicine Lianhua Qingwen. Chin. J. Exp. Traditional Med. Formulae. 26 (11), 8–12. 10.13422/j.cnki.syfjx.20201099

[B38] Ye C. Gao M. Lin W. Yu K. Li P. Chen G. (2020). Theoretical Study of the Anti-ncp Molecular Mechanism of Traditional Chinese Medicine Lianhua-Qingwen Formula (Lqf). ChemRxiv. Available at: https://chemrxiv.org/engage/chemrxiv/article-details/60c74908ee301c485bc799cf (Accessed July 23, 2021). 10.26434/chemrxiv.12016236.v1

[B39] Yiling Pharmaceutical (2020). Lianhua Qingwen Capsules of Yiling Pharmaceutical Approved in mauritius. Business China. (10), 100.

[B40] Yiling Pharmaceutical (2021). Lianhua Qingwen Capsules/granules Product Details. Available at: http://www.yiling.cn/contents/75/132.html (Accessed September 14, 2021).

[B41] Zhang R. X. Liu W. W. (2014). Meta-Analysis of Efficacy of Oseltamivir and Substitution Therapy for Anti- H1n1 Infection. China Med. Herald. 11 (31), 5260–5355.

