# Supplementary material for: The Adverse Reactions of Lianhua Qingwen Capsule/Granule Compared With Conventional Drug in Clinical Application: A Meta-Analysis
Source: Front Pharmacol. 2022 Jan 27;13:764774. doi: 10.3389/fphar.2022.764774 (PMC8830515; doi:10.3389/fphar.2022.764774)
Supplement: Supplementary file 1 [file Table1.DOCX]

# Supplementary Tables

# 1 Supplementary Table 1. Main characteristics of the eligible studies

| Studies | Disease | Lianhua Qingwen group | | |  | Conventional drug group | | | Adverse reactions | Dispose or outcome of adverse reactions |
| --- | --- | --- | --- | --- | --- | --- | --- | --- | --- | --- |
|  |  | S | M/F | Age |  | S | M/F | Age |  |  |
| Liu et al.,2021 | COVID-19 | 68 | 32/36 | 59.5±15.6 |  | 40 | 15/25 | 54.8±19.1 | None. |  |
| Wang et al.,2021 | Hand-foot- mouth disease | 60 | 32/28 | 2-12 |  | 60 | 30/30 | 1-11 | Gastrointestinal system damage: Loss of appetite, Vomiting, Nausea. |  |
| Hu et al.,2021 | COVID-19 | 142 | 79/63 | 50.4±15.2 |  | 142 | 71/71 | 51.8±14.8 | Gastrointestinal system damage: Loss of appetite, Vomiting, Diarrhea, Nausea. |  |
|  |  |  |  |  |  |  |  |  | Nervous system damage: Headache. |  |
|  |  |  |  |  |  |  |  |  | Hepatobiliary system damage: Hepatic function abnormal. |  |
|  |  |  |  |  |  |  |  |  | Other symptoms: Renal insufficiency. |  |
| Lin et al.,2020 | Nasosinusitis | 70 | 36/34 | 51(25-71) |  | 70 | 33/37 | 54(18-75) | Gastrointestinal system damage: Gastrointestinal distress. | Recovered after symptomatic treatment |
| Cai et al.,2020 | Respiratory tract infection | 69 | 36/33 | 3.10±0.35 |  | 69 | 40/29 | 3.11±0.32 | Gastrointestinal system damage: Gastrointestinal distress. |  |
|  |  |  |  |  |  |  |  |  | Skin and its appendage damage: Rash. |  |
|  |  |  |  |  |  |  |  |  | Respiratory system damage: Foreign body sensation of respiratory tract. |  |
| Zeng et al.,2020 | Influenza | 49 | 25/24 | 6 |  | 49 | 24/25 | 6 | Gastrointestinal system damage: Nausea, Diarrhea, Vomiting. |  |
| Chao et al.,2020 | Viral keratitis | 41 | 16/25 | 39.38±6.61 |  | 41 | 18/23 | 39.72±6.45 | None. |  |
| Chen et al.,2020a | Common pneumonia | 52 | 26/26 | 5.45±0.55 |  | 52 | 23/29 | 5.37±0.38 | Gastrointestinal system damage: Gastrointestinal distress. |  |
|  |  |  |  |  |  |  |  |  | Nervous system damage: Headache. |  |

**Supplementary Table 1 continued.**

| Studies | Disease | Lianhua Qingwen group | | |  | Conventional drug group | | | Adverse reactions | Dispose or outcome of adverse reactions |
| --- | --- | --- | --- | --- | --- | --- | --- | --- | --- | --- |
|  |  | S | M/F | Age |  | S | M/F | Age |  |  |
| Chen et al.,2020b | Common pneumonia | 79 | 45/34 | 7.81±1.64 |  | 78 | 42/36 | 7.77±1.53 | Heart rate and arrhythmia: Tachycardia. |  |
|  |  |  |  |  |  |  |  |  | Gastrointestinal system damage: Diarrhea, Abdominal pain. |  |
|  |  |  |  |  |  |  |  |  | Nervous system damage: Headache. |  |
|  |  |  |  |  |  |  |  |  | Skin and its appendage damage: Flushing, Rash. |  |
| Fang et al.,2020 | COVID-19 | 42 | 18/24 | 4.8±3.7 |  | 41 | 16/23 | 3.9±3.2 | None. |  |
| Fu 2020 | Influenza | 124 | 69/55 | 50.17±14.09 |  | 124 | 72/52 | 50.15±14.04 | Gastrointestinal system damage: Abdominal distension, Diarrhea. |  |
|  |  |  |  |  |  |  |  |  | Skin and its appendage damage: Rash. |  |
| Guo et al.,2020 | Influenza | 38 | 21/17 | 40.07±3.67 |  | 38 | 20/18 | 40.18±3.54 | Gastrointestinal system damage: Diarrhea, Nausea. |  |
|  |  |  |  |  |  |  |  |  | Nervous system damage: Dizziness. |  |
|  |  |  |  |  |  |  |  |  | Body as a whole-general disorders: Sleepy. |  |
| Jian 2020 | Influenza | 42 | 24/18 | 42.09±7.58 |  | 45 | 26/19 | 41.23±7.36 | Gastrointestinal system damage: Gastrointestinal distress, Vomiting, Diarrhea. |  |
| Jiang 2020 | Nose bleeding | 60 | 35/25 | 44.1±4.3 |  | 60 | 33/27 | 41.7±4.9 | None. |  |
| Lei 2020 | Influenza | 49 | 32/17 | 7.15±1.08 |  | 48 | 30/18 | 7.19±1.05 | Gastrointestinal system damage: Diarrhea, Nausea. |  |
| Li et al.,2020 | Respiratory tract infection | 56 | 30/26 | 5.67±1.42 |  | 56 | 33/23 | 5.83±1.46 | Gastrointestinal system damage: Gastrointestinal symptoms. | Improved spontaneously without any treatment |

**Supplementary Table 1 continued.**

| Studies | Disease | Lianhua Qingwen group | | |  | Conventional drug group | | | Adverse reactions | Dispose or outcome of adverse reactions |
| --- | --- | --- | --- | --- | --- | --- | --- | --- | --- | --- |
|  |  | S | M/F | Age |  | S | M/F | Age |  |  |
| Li 2020a | Influenza | 30 | 16/14 | 47.93±3.54 |  | 30 | 17/13 | 47.93±3.54 | Gastrointestinal system damage: Abdominal distension, Diarrhea. |  |
| Li 2020b | Respiratory tract infection | 50 | 26/24 | 40.75±8.03 |  | 46 | 24/22 | 38.75±9.05 | Only the number of adverse reactions was reported. |  |
| Liu 2020a | Influenza | 40 | 23/17 | 26.95±6.78 |  | 37 | 21/16 | 26.62±6.48 | Gastrointestinal system damage: Nausea, Vomiting, Diarrhea. |  |
| Liu 2020b | Common pneumonia | 51 | 26/25 | 7.46±1.25 |  | 51 | 24/27 | 7.08±1.40 | Gastrointestinal system damage: Vomiting, Diarrhea, Rash. |  |
| Liu 2020c | Influenza | 60 | 30/30 | 34.12±6.26 |  | 60 | 32/28 | 32.39±5.98 | Gastrointestinal system damage: Abdominal distension, Diarrhea, Gastrointestinal distress. |  |
| Liu et al.,2020 | Influenza | 30 | 15/15 | 4.32±1.41 |  | 30 | 16/14 | 4.78±1.35 | Respiratory system damage: Cough. |  |
|  |  |  |  |  |  |  |  |  | Other symptoms: Relapse of fever. |  |
| Lu et al.,2020 | Common pneumonia | 140 | 76/64 | 52.71±8.63 |  | 140 | 78/52 | 52.68 8.59 | Gastrointestinal system damage: Gastrointestinal symptoms. |  |
|  |  |  |  |  |  |  |  |  | Skin and its appendage damage: Rash. |  |
|  |  |  |  |  |  |  |  |  | Psychiatric disorders: Upset, Sleep disorder. |  |
|  |  |  |  |  |  |  |  |  | Psychiatric disorders: |  |
|  |  |  |  |  |  |  |  |  | Other symptoms: Blurred vision, Adverse cardiac reactions. |  |
| Lv et al.,2020 | COVID-19 | 63 | 28/35 | 59.12±16.56 |  | 38 | 18/20 | 60.20±17.01 | Gastrointestinal system damage: Diarrhea, Nausea, Vomiting, Loss of appetite. |  |
| Shi 2020 | Viral influenza | 51 | 27/24 | 32.60±8.20 |  | 51 | 28/23 | 32.57±8.17 | Gastrointestinal system damage: Diarrhea, Nausea, Vomiting. |  |
|  |  |  |  |  |  |  |  |  | Nervous system damage: Neurological Symptoms |  |

**Supplementary Table 1 continued.**

| Studies | Disease | Lianhua Qingwen group | | |  | Conventional drug group | | | Adverse reactions | Dispose or outcome of adverse reactions |
| --- | --- | --- | --- | --- | --- | --- | --- | --- | --- | --- |
|  |  | S | M/F | Age |  | S | M/F | Age |  |  |
| Sun, et al.,2020 | Respiratory tract infection | 44 | 23/21 | 1.05±0.62 |  | 43 | 23/20 | 1.02±0.73 | Gastrointestinal system damage: Abdominal discomfort, Diarrhea, Nausea, Vomiting. |  |
|  |  |  |  |  |  |  |  |  | Nervous system damage: Dizziness. |  |
|  |  |  |  |  |  |  |  |  | Body as a whole-general disorders: Sleepy. |  |
| Tang et al.,2020 | Influenza | 82 | 43/39 | 35.23(5-75) |  | 82 | 45/37 | 34.32(5-75) | Gastrointestinal system damage: Diarrhea, Nausea, Vomiting. |  |
|  |  |  |  |  |  |  |  |  | Nervous system damage: Dizziness. |  |
|  |  |  |  |  |  |  |  |  | Skin and its appendage damage: Rash. |  |
| Wan et al.,2020 | Influenza | 47 | 24/23 | 45.7±0.7 |  | 47 | 25/22 | 46.3±0.6 | Heart rate and arrhythmia: Palpitations. |  |
|  |  |  |  |  |  |  |  |  | Gastrointestinal system damage: Diarrhea, Nausea. |  |
|  |  |  |  |  |  |  |  |  | Nervous system damage: Dizziness. |  |
| Wang et al.,2020 | Common pneumonia | 60 | 27/33 | 7.7±2.8 |  | 60 | 29/31 | 7.5±2.4 | Heart rate and arrhythmia: Arrhythmia.  Gastrointestinal system damage: Abdominal pain, Dyspepsia, Diarrhea.  Nervous system damage: Dizziness, Drowsiness, Headache.  Skin and its appendage damage: Rash. |  |
|  |  |  |  |  |  |  |  |  |  |  |
|  |  |  |  |  |  |  |  |  |  |  |
|  |  |  |  |  |  |  |  |  |  |  |
| Wang 2020 | Influenza | 40 | — | — |  | 40 | — | — | Gastrointestinal system damage: Diarrhea, Nausea, Vomiting. |  |
|  |  |  |  |  |  |  |  |  | Nervous system damage: Dizziness. |  |
| Wei, 2020 | Influenza | 21 | 11/10 | 46.03±7.71 |  | 21 | 12/9 | 46.11±7.69 | Gastrointestinal system damage: Diarrhea, Nausea, Vomiting. |  |
| Xu et al.,2020 | Common pneumonia | 48 | 14/34 | 51.3±10.4 |  | 48 | 13/35 | 50.3±12.0 | Gastrointestinal system damage: Abdominal pain, Diarrhea, Nausea, Vomiting. |  |

**Supplementary Table 1 continued.**

| Studies | Disease | Lianhua Qingwen group | | |  | Conventional drug group | | | Adverse reactions | Dispose or outcome of adverse reactions |
| --- | --- | --- | --- | --- | --- | --- | --- | --- | --- | --- |
|  |  | S | M/F | Age |  | S | M/F | Age |  |  |
| Yu et al.,2020 | COVID-19 | 147 | 82/65 | 48.27±9.56 |  | 148 | 89/59 | 47.25±8.67 | None. |  |
| Zhang 2020a | Influenza | 49 | 22/27 | 42.87±1.22 |  | 49 | 24/25 | 43.09±1.34 | Gastrointestinal system damage: Abdominal pain, Abdominal distension, Diarrhea, Nausea, Vomiting. |  |
|  |  |  |  |  |  |  |  |  | Nervous system damage: Dizziness. |  |
| Zhang 2020b | Influenza | 25 | 12/13 | 5.12±0.15 |  | 25 | 12/13 | 25.13±0.16 | Gastrointestinal system damage: Diarrhea, Nausea, Vomiting. |  |
|  |  |  |  |  |  |  |  |  | Nervous system damage: Dizziness. |  |
|  |  |  |  |  |  |  |  |  | Skin and its appendage damage: Rash. |  |
| Zhang 2020c | Herpes pharyngitis | 57 | 23/24 | 2.9±0.4 |  | 43 | 22/21 | 2.9±0.4 | Gastrointestinal system damage: Diarrhea, Nausea, Vomiting. |  |
| Du et al.,2019 | Viral keratitis | 72 | 25/47 | 45±14 |  | 76 | 30/46 | 42±14 | None. |  |
| Feng et al.,2019 | Respiratory tract infection | 60 | 21/39 | — |  | 60 | 24/36 | — | None. |  |
| Han 2019 | Influenza | 45 | 26/19 | 36.74±3.05 |  | 45 | 25/20 | 36.21±3.11 | Gastrointestinal system damage: Nausea, Vomiting. |  |
|  |  |  |  |  |  |  |  |  | Nervous system damage: Dizziness. |  |
|  |  |  |  |  |  |  |  |  | Other symptoms: Abnormal blood pressure. |  |
| Hao 2019 | Influenza A (H1N1) | 33 | 17/16 | 27.14±2.17 |  | 33 | 18/15 | 27.35±2.51 | Gastrointestinal system damage: Nausea, Vomiting. |  |
| Hua et al.,2019 | Influenza | 51 | 24/27 | 43±5 |  | 51 | 22/29 | 44±5 | Gastrointestinal system damage: Diarrhea. | Healed with taking medication after meals |
| Ji 2019 | Tonsillitis | 30 | 15/15 | 18.9±1.2 |  | 30 | 16/14 | 18.7±1.1 | None. |  |

**Supplementary Table 1 continued.**

| Studies | Disease | Lianhua Qingwen group | | |  | Conventional drug group | | | Adverse reactions | Dispose or outcome of adverse reactions |
| --- | --- | --- | --- | --- | --- | --- | --- | --- | --- | --- |
|  |  | S | M/F | Age |  | S | M/F | Age |  |  |
| Li 2019 | Respiratory tract infection | 60 | 38/32 | 28.7±10.3 |  | 66 | 36/30 | 27.6±10.2 | None. |  |
| Liang 2019 | Rheum | 100 | 69/31 | 30.29±3.74 |  | 100 | 68/32 | 30.30±3.72 | Gastrointestinal system damage: Abdominal discomfort, Nausea, Vomiting. |  |
| Liang et al.,2019 | Influenza | 49 | 32/17 | 8.2±2.7 |  | 49 | 30/19 | 7.8±3.2 | Gastrointestinal system damage: Nausea. |  |
|  |  |  |  |  |  |  |  |  | Nervous system damage. |  |
| Mo et al.,2019 | Hand-foot- mouth disease | 51 | 30/21 | 2.4±0.7 |  | 51 | 27/24 | 2.2±0.5 | Gastrointestinal system damage: Diarrhea, Nausea. |  |
| Sun 2019 | Common pneumonia | 41 | 28/13 | 51.06±5.92 |  | 41 | 27/14 | 50.17±5.68 | Gastrointestinal system damage: Diarrhea, Nausea, Vomiting. |  |
| Tang et al.,2019 | chicken pox | 28 | — | 32.09(26-43) |  | 29 | — | 33.05(29-49) | Only the number of adverse reactions was reported. |  |
| Wang 2019 | Influenza | 30 | 18/12 | 30.41±10.12 |  | 30 | 19/11 | 30.40±10.13 | Gastrointestinal system damage: Diarrhea. |  |
|  |  |  |  |  |  |  |  |  | Secondary infection: Nasosinusitis, Tonsillitis. |  |
| Wen et al.,2019 | Respiratory tract infection | 20 | 20/0 | 21.45±1.28 |  | 20 | 20/0 | 21.79±1.51 | Only the number of adverse reactions was reported. |  |
| Xia et al.,2019 | Common pneumonia | 55 | 55/0 | — |  | 55 | 55/0 | — | Gastrointestinal system damage: Nausea, Vomiting. |  |
|  |  |  |  |  |  |  |  |  | Nervous system damage: Dizziness. |  |
|  |  |  |  |  |  |  |  |  | Skin and its appendage damage: Rash. |  |
| Zhang et al.,2019 | Influenza | 40 | 19/21 | 37.1±9.6 |  | 40 | 16/24 | 35.4±10.2 | Gastrointestinal system damage: Diarrhea, Nausea, Vomiting. |  |
|  |  |  |  |  |  |  |  |  | Nervous system damage: Dizziness. |  |

**Supplementary Table 1 continued.**

| Studies | Disease | Lianhua Qingwen group | | |  | Conventional drug group | | | Adverse reactions | Dispose or outcome of adverse reactions |
| --- | --- | --- | --- | --- | --- | --- | --- | --- | --- | --- |
|  |  | S | M/F | Age |  | S | M/F | Age |  |  |
| Zhang 2019 | Respiratory tract infection | 96 | 50/46 | 49.25±3.52 |  | 96 | 55/41 | 49.55±3.20 | None. |  |
| Zhou 2019 | Influenza | 54 | 29/25 | 31.82±8.57 |  | 54 | 28/26 | 31.77±8.52 | Gastrointestinal system damage: Nausea, Vomiting. |  |
| Bai et al.,2019 | Chronic obstructive pulmonary disease | 60 | 36/24 | 57.2±7.7 |  | 40 | 25/15 | 56.8±6.9 | Gastrointestinal system damage: Nausea, Vomiting. |  |
|  |  |  |  |  |  |  |  |  | Skin and its appendage damage: Rash. |  |
| Cheng 2018 | Viral influenza | 30 | 15/15 | 42.5±6.8 |  | 30 | 16/14 | 43.7±7.3 | Gastrointestinal system damage: Gastrointestinal distress. | Improved with taking medication after meals |
| Hu 2018 | Influenza | 80 | 50/30 | 42.61± 5.23 |  | 80 | 45/35 | 43.62± 5.11 | None. |  |
| Hu et al.,2018 | Common pneumonia | 52 | 37/15 | 43.4±4.8 |  | 52 | 36/16 | 44.7±4.1 | Gastrointestinal system damage: Gastrointestinal distress, Loss of appetite, Nausea, vomiting. | Not dispose adverse reactions |
| Huang 2018 | Viral influenza | 55 | 23/32 | 56.3(12-58) |  | 55 | 24/31 | 153.9(3-57) | Gastrointestinal system damage: Diarrhea, Nausea, Vomiting. | Recovered after symptomatic treatment |
|  |  |  |  |  |  |  |  |  | Skin and its appendage damage: Itchy skin. |  |
| Kong 2018 | Influenza | 80 | 34/44 | 47.5±9.7 |  | 60 | 28/32 | 46.4±9.2 | Gastrointestinal system damage: Nausea |  |
|  |  |  |  |  |  |  |  |  | Secondary infection: Nasosinusitis, Amygdalitis. |  |

**Supplementary Table 1 continued.**

| Studies | Disease | Lianhua Qingwen group | | |  | Conventional drug group | | | Adverse reactions | Dispose or outcome of adverse reactions |
| --- | --- | --- | --- | --- | --- | --- | --- | --- | --- | --- |
|  |  | S | M/F | Age |  | S | M/F | Age |  |  |
| Li 2018 | Respiratory tract infection | 43 | 24/19 | 21.36±1.94 |  | 43 | 23/20 | 20.11±2.07 | Gastrointestinal system damage: Gastrointestinal distress, Nausea, Vomiting. |  |
|  |  |  |  |  |  |  |  |  | Skin and its appendage damage: Rash. |  |
| Li et al.,2018a | Influenza | 43 | 22/21 | 6.3±0.58 |  | 40 | 20/20 | 6.2±0.55 | Gastrointestinal system damage: Diarrhea, Nausea, Vomiting. | Improved spontaneously without any treatment |
| Li et al.,2018b | Respiratory tract infection | 50 | 26/24 | 5.35±0.40 |  | 50 | 25/25 | 5.15±0.30 | Gastrointestinal system damage: Abdominal pain, Diarrhea, Nausea. |  |
| Liu 2018 | Viral influenza | 500 | — | 42.6±6.9 |  | 500 | — | 43.8±7.2 | Gastrointestinal system damage: Diarrhea, Nausea, Vomiting. |  |
|  |  |  |  |  |  |  |  |  | Skin and its appendage damage: Rash. |  |
| Lv et al.,2018 | Common pneumonia | 60 | 29/31 | 48.42±7.59 |  | 60 | 27/33 | 48.61±7.73 | Gastrointestinal system damage: Abdominal pain, Nausea. |  |
|  |  |  |  |  |  |  |  |  | Skin and its appendage damage: Rash. |  |
| Qiao 2018 | Respiratory tract infection | 53 | 30/23 | 6.62±2.79 |  | 53 | 31/22 | 6.41±2.98 | Gastrointestinal system damage: Gastrointestinal distress. |  |
|  |  |  |  |  |  |  |  |  | Skin and its appendage damage: Rash. |  |
|  |  |  |  |  |  |  |  |  | Abnormal white blood cells and reticuloendothelial system: Leukopenia. |  |
| Wang 2018 | Influenza | 45 | 35/10 | 25.84±2.38 |  | 45 | 32/13 | 26.24±3.14 | None. |  |
| Sun 2018 | Influenza | 46 | — | — |  | 46 | — | — | Gastrointestinal system damage: Diarrhea, Nausea, Vomiting, Gastrointestinal distress. |  |
| Tang 2018 | Respiratory tract infection | 30 | — | — |  | 30 | — | — | Gastrointestinal system damage: Nausea, Abdominal discomfort. |  |

**Supplementary Table 1 continued.**

| Studies | Disease | Lianhua Qingwen group | | |  | Conventional drug group | | | Adverse reactions | Dispose or outcome of adverse reactions |
| --- | --- | --- | --- | --- | --- | --- | --- | --- | --- | --- |
|  |  | S | M/F | Age |  | S | M/F | Age |  |  |
| Wang 2018 | Respiratory tract infection | 40 | 24/16 | 33.8±1.8 |  | 40 | 25/15 | 34.5±2.0 | Only the number of adverse reactions was reported. |  |
| Zhang 2018a | Common pneumonia | 39 | 24/15 | 69.4±2.1 |  | 39 | 23/16 | 69.2±2.0 | Psychiatric disorders: Hallucination.  Body as a whole-general disorders: Edema.  Other symptoms: Abnormal blood pressure.  Hepatobiliary system damage: Increased glutamylaminopeptidase. |  |
|  |  |  |  |  |  |  |  |  |  |  |
|  |  |  |  |  |  |  |  |  |  |  |
|  |  |  |  |  |  |  |  |  |  |  |
| Zhang 2018b | Common pneumonia | 60 | 32/28 | 46.81±9.22 |  | 60 | 33/27 | 46.91±9.34 | Gastrointestinal system damage: Diarrhea. |  |
|  |  |  |  |  |  |  |  |  | Skin and its appendage damage: Rash. |  |
|  |  |  |  |  |  |  |  |  | Hepatobiliary system damage: Elevated aspartate transferase. |  |
| Zhang 2018c | Influenza | 30 | 16/14 | 33.2±4.8 |  | 30 | 17/13 | 32.9±4.6 | None. |  |
| Zhang 2018d | Influenza | 150 | 75/75 | 44.1±1.4 |  | 150 | 76/74 | 44.2±1.15 | Gastrointestinal system damage: Diarrhea, Gastrointestinal distress. |  |
| Zhou 2018 | Influenza A (H1N1) | 25 | 13/12 | 28.9±2.1 |  | 25 | 11/14 | 27.3±1.2 | Gastrointestinal system damage: Nausea, vomiting. | Improved after symptomatic treatment |
| Chen 2017 | Herpes zoster | 30 | 16/14 | 49±10 |  | 26 | 14/12 | 48±9 | Only the number of adverse reactions was reported. | Not dispose adverse reactions |
| Huang 2017 | Influenza | 39 | 21/18 | 31.85±1.64 |  | 39 | 22/17 | 32.57±1.62 | None. |  |
| Huang et al.,2017a | Hand-foot- mouth disease | 79 | 42/37 | 3.2±0.3 |  | 79 | 40/39 | 2.9±0.7 | Gastrointestinal system damage: Diarrhea, Nausea, Vomiting. |  |
|  |  |  |  |  |  |  |  |  | Nervous system damage: Headache. |  |
|  |  |  |  |  |  |  |  |  | Respiratory system damage: Dyspnea. |  |

**Supplementary Table 1 continued.**

| Studies | Disease | Lianhua Qingwen group | | |  | Conventional drug group | | | Adverse reactions | Dispose or outcome of adverse reactions |
| --- | --- | --- | --- | --- | --- | --- | --- | --- | --- | --- |
|  |  | S | M/F | Age |  | S | M/F | Age |  |  |
| Huang et al.,2017b | Hand-foot- mouth disease | 45 | 26/19 | 4.56±0.34 |  | 45 | 25/20 | 4.45±12.24 | Gastrointestinal system damage: Diarrhea, Dry mouth. |  |
| Liao 2017 | Influenza | 90 | 52/38 | 29.87±9.04 |  | 90 | 49/41 | 30.96±10.15 | None. |  |
| Liu et al.,2017 | Common pneumonia | 53 | 30/23 | 40.34±9.23 |  | 53 | 32/21 | 42.13±8.18 | None. |  |
| Ma 2017 | Rheum | 36 | 16/20 | 34.31±13.13 |  | 36 | 19/17 | 36.08±14.00 | Hepatobiliary system damage: Transaminase increased. |  |
| Qin 2017 | Influenza | 60 | 33/27 | 27.6±4.1 |  | 60 | 35/25 | 28.1±3.5 | None. |  |
| Wang 2017 | Hand-foot- mouth disease | 68 | 42/26 | 1.46 |  | 68 | 47/21 | 1.39 | Gastrointestinal system damage: Diarrhea, Nausea, Vomiting. |  |
|  |  |  |  |  |  |  |  |  | Other symptoms: Hemoglobin drops. |  |
| Yu et al.,2017 | Rheum | 50 | — | — |  | 50 | — | — | Gastrointestinal system damage: Diarrhea. | Recovered after symptomatic treatment |
| Zhou 2017 | Rheum | 70 | 45/25 | 37.6±7.5 |  | 70 | 32/38 | 36.8±7.2 | Gastrointestinal system damage: Gastrointestinal distress. |  |
|  |  |  |  |  |  |  |  |  | Skin and its appendage damage: Rash. |  |
|  |  |  |  |  |  |  |  |  | Hepatobiliary system damage: Transaminase increased. |  |
| Chen 2016 | Respiratory tract infection | 30 | 21/9 | 22.57±3.68 |  | 30 | 21/9 | 23.10±3.40 | None. |  |
| Fang et al.,2016 | Respiratory tract infection | 95 | — | 30.3±11.8 |  | 81 | — | 31.4±11.7 | Gastrointestinal system damage: Gastrointestinal distress. | Not dispose adverse reactions |
| Feng et al.,2016 | Pharyngitis | 51 | 24/27 | 14-40 |  | 51 | 26/25 | 14-40 | None. |  |

**Supplementary Table 1 continued.**

| Studies | Disease | Lianhua Qingwen group | | |  | Conventional drug group | | | Adverse reactions | Dispose or outcome of adverse reactions |
| --- | --- | --- | --- | --- | --- | --- | --- | --- | --- | --- |
|  |  | S | M/F | Age |  | S | M/F | Age |  |  |
| Han 2016 | Hand-foot- mouth disease | 60 | 33/27 | 2.8±1.6 |  | 60 | 34/26 | 2.7±1.7 | None. |  |
| Jin 2016 | Influenza | 80 | — | — |  | 80 | — | — | Gastrointestinal system damage: Gastrointestinal distress. |  |
|  |  |  |  |  |  |  |  |  | Skin and its appendage damage: Rash. |  |
|  |  |  |  |  |  |  |  |  | Hepatobiliary system damage: Transaminase increased. |  |
| Li 2016 | Influenza | 63 | 24/39 | 29.2±5.4 |  | 63 | 23/40 | 29.4±5.3 | Gastrointestinal system damage: Diarrhea, Nausea. |  |
|  |  |  |  |  |  |  |  |  | Nervous system damage. |  |
| Li et al.,2016 | Viral influenza | 41 | 23/18 | 39.5±1.2 |  | 41 | 24/17 | 39.1±1.6 | Gastrointestinal system damage: Gastrointestinal distress. |  |
| Qiu 2016 | Tonsillitis | 40 | — | 20.15±3.08 |  | 40 | — | 20.15±3.66 | Gastrointestinal system damage: Diarrhea, Nausea, Vomiting. |  |
|  |  |  |  |  |  |  |  |  | Skin and its appendage damage: Rash. |  |
| Shi et al.,2016 | Common pneumonia | 39 | 16/23 | 5.58±2.87 |  | 34 | 12/22 | 6.83±2.94 | None. |  |
| Wang 2016 | Influenza | 40 | 25/15 | 35.64±2.58 |  | 40 | 18/22 | 34.69±2.64 | Only the number of adverse reactions was reported. |  |
| Wei 2016 | Chronic obstructive pulmonary disease | 40 | 20/20 | 65.74±3.38 |  | 40 | 21/19 | 65.77±3.41 | None. |  |

**Supplementary Table 1 continued.**

| Studies | Disease | Lianhua Qingwen group | | |  | Conventional drug group | | | Adverse reactions | Dispose or outcome of adverse reactions |
| --- | --- | --- | --- | --- | --- | --- | --- | --- | --- | --- |
|  |  | S | M/F | Age |  | S | M/F | Age |  |  |
| Yang 2016a | Coughing phlegm heat depression lung syndrome | 60 | 21/39 | 40.4±6.1 |  | 60 | 35/25 | 41.2±4.5 | None. |  |
| Yang 2016b | Common pneumonia | 38 | — | — |  | 22 | — | — | Gastrointestinal system damage: Gastrointestinal distress. | Improved spontaneously without any treatment |
| Zhang et al.,2016 | Influenza A (H1N1) | 35 | — | — |  | 35 | — | — | Heart rate and arrhythmia: Palpitations.  Gastrointestinal system damage: Diarrhea.  Nervous system damage: Dizziness.  Other symptoms: Increased neutrophils.  Secondary infection: Pulmonary infection. |  |
|  |  |  |  |  |  |  |  |  |  |  |
|  |  |  |  |  |  |  |  |  |  |  |
|  |  |  |  |  |  |  |  |  |  |  |
|  |  |  |  |  |  |  |  |  |  |  |
| Zhang 2016 | Respiratory tract infection | 45 | 22/23 | 35.2±2.3 |  | 45 | 25/20 | 34.8±1.7 | Only the number of adverse reactions was reported. |  |
| Bi et al.,2015 | Chronic obstructive pulmonary disease | 36 | 25/11 | 61.2±10.8 |  | 36 | 23/13 | 61.5±11.2 | None. |  |
| Dong 2015 | Viral influenza | 37 | 22/15 | 23.62±7.56 |  | 36 | 20/16 | 22.56±7.68 | Gastrointestinal system damage: Abdominal discomfort, Diarrhea, Nausea. |  |
|  |  |  |  |  |  |  |  |  | Nervous system damage: Dizziness. |  |
|  |  |  |  |  |  |  |  |  | Body as a whole-general disorders: Sleepy. |  |

**Supplementary Table 1 continued.**

| Studies | Disease | Lianhua Qingwen group | | |  | Conventional drug group | | | Adverse reactions | Dispose or outcome of adverse reactions |
| --- | --- | --- | --- | --- | --- | --- | --- | --- | --- | --- |
|  |  | S | M/F | Age |  | S | M/F | Age |  |  |
| Du et al.,2015 | keratitis | 43 | 22/21 | 45.58±16.69 |  | 41 | 23/18 | 45.17±16.37 | Other symptoms: Eye irritation, Itching and foreign body sensation, Resurgence of disease. |  |
| Fang 2015 | Herpes zoster | 35 | 15/20 | 54.31±8.52 |  | 35 | 17/18 | 53.50±7.95 | None. |  |
| Feng 2015 | Malaria | 50 | 30/20 | 32.6±1.25 |  | 50 | 30/20 | 35.2±1.28 | Other symptoms: Resurgence of disease. |  |
| Guo 2015 | Influenza A (H1N1) | 51 | 27/24 | 32.87(15-62) |  | 51 | 28/23 | 32.32(16-61) | Gastrointestinal system damage: Diarrhea, Nausea, Vomiting. |  |
|  |  |  |  |  |  |  |  |  | Nervous system damage. |  |
|  |  |  |  |  |  |  |  |  | Secondary infection: Pulmonary infection. |  |
| He 2015 | Viral influenza | 90 | — | — |  | 90 | — | — | Gastrointestinal system damage: Gastrointestinal distress. | Recovered after symptomatic treatment |
|  |  |  |  |  |  |  |  |  | Nervous system damage: Drowsiness. |  |
| Li et al.,2015 | Respiratory tract infection | 60 | 35/25 | 41±12 |  | 60 | 31/29 | 43±10 | None. |  |
| Li 2015a | Herpetic stomatitis | 40 | 22/18 | — |  | 40 | 16/24 | — | None. |  |
| Li 2015b | Herpes zoster | 72 | 35/37 | 49.3±13.3 |  | 73 | 36/37 | 51.1±11.5 | Gastrointestinal system damage: Gastrointestinal distress.  Nervous system damage: Legacy neuralgia. |  |
|  |  |  |  |  |  |  |  |  |  |  |
| Lin et al.,2015 | Nasosinusitis | 34 | — | — |  | 61 | — | — | None. |  |
| Liu et al.,2015 | Chronic obstructive pulmonary disease | 58 | 36/22 | 66.33±3.56 |  | 58 | 33/25 | 69.17±2.82 | None. |  |

**Supplementary Table 1 continued.**

| Studies | Disease | Lianhua Qingwen group | | |  | Conventional drug group | | | Adverse reactions | Dispose or outcome of adverse reactions |
| --- | --- | --- | --- | --- | --- | --- | --- | --- | --- | --- |
|  |  | S | M/F | Age |  | S | M/F | Age |  |  |
| Liu 2015 | Respiratory tract infection | 45 | 29/16 | 34.5±8.9 |  | 42 | 26/16 | 35.0±8.2 | Gastrointestinal system damage: Gastrointestinal distress, Vomiting. |  |
|  |  |  |  |  |  |  |  |  | Skin and its appendage damage: Rash. |  |
| Lu et al.,2015 | Coughing phlegm heat depression lung syndrome | 50 | 14/36 | 46.84(19-75) |  | 50 | 27/23 | 46.88(23-79) | None. |  |
| Ma 2015 | Influenza | 86 | 43/43 | 47.8 |  | 70 | 38/32 | 49.3 | None. |  |
| Ma et al.,2015 | Herpangina | 45 | 20/25 | 2.5±1.2 |  | 45 | 23/22 | 2.5±1.6 | None. |  |
| Meng et al.,2015 | Common pneumonia | 20 | 12/8 | 50.7±3.4 |  | 18 | 10/8 | 51.4±4.2 | Gastrointestinal system damage: Nausea. |  |
|  |  |  |  |  |  |  |  |  | Skin and its appendage damage: Rash. |  |
| Wang 2015 | Respiratory tract infection | 43 | 28/15 | 23 |  | 43 | 29/17 | 21 | None. |  |
| Wang et al.,2015 | Common pneumonia | 34 | — | 70.5(61-75) |  | 34 | — | 69.5(59-74) | Gastrointestinal system damage: Loss of appetite. |  |
|  |  |  |  |  |  |  |  |  | Nervous system damage: Dizziness, headache. |  |
|  |  |  |  |  |  |  |  |  | Skin and its appendage damage: Itchy skin. |  |
| Wen et al.,2015 | Influenza | 72 | — | — |  | 70 | — | — | None. |  |
| Wu et al.,2015 | Chronic obstructive pulmonary disease | 50 | — | — |  | 50 | — | — | Gastrointestinal system damage: Constipation, Dry mouth, Nausea | Healed spontaneously after stopping the medication |

**Supplementary Table 1 continued.**

| Studies | Disease | Lianhua Qingwen group | | |  | Conventional drug group | | | Adverse reactions | Dispose or outcome of adverse reactions |
| --- | --- | --- | --- | --- | --- | --- | --- | --- | --- | --- |
|  |  | S | M/F | Age |  | S | M/F | Age |  |  |
| Xu et al.,2015 | Common pneumonia | 38 | 18/20 | 35.6±12.8 |  | 30 | 14/16 | 34.8±13.2 | Only the number of adverse reactions was reported. |  |
| Zhang et al.,2015 | Common pneumonia | 41 | — | — |  | 41 | — | — | Only the number of adverse reactions was reported. | Recovered after symptomatic treatment |
| Zhao et al.,2015 | Respiratory tract infection | 500 | 346/154 | 34.6±10.8 |  | 500 | 319/181 | 35.1±11.2 | None. | Improved with taking medication after meals |
| Zheng et al.,2015 | Influenza | 64 | 32/32 | 8-12 |  | 64 | 33/31 | 4-7 | Gastrointestinal system damage: Nausea, Vomiting. | Improved spontaneously without any treatment |
| Zhou 2015 | Hand-foot- mouth disease | 133 | 77/56 | 3.5±1.0 |  | 124 | 70/54 | 3.9±1.0 | Gastrointestinal system damage: Dyspepsia, Nausea, Vomiting. |  |
|  |  |  |  |  |  |  |  |  | Nervous system damage: Headache, Vertigo. |  |
|  |  |  |  |  |  |  |  |  | Respiratory system damage: Dyspnea. |  |
| Zhu et al.,2015 | Lung infection | 31 | 23/8 | 47. 0 ± 3. 0 |  | 31 | 21/10 | 48. 5±3. 5 | Gastrointestinal system damage: Gastrointestinal distress. |  |
|  |  |  |  |  |  |  |  |  | Skin and its appendage damage: Rash |  |
|  |  |  |  |  |  |  |  |  | Abnormal white blood cells and. reticuloendothelial system: Leukopenia. |  |
| Zhou et al.,2015 | Influenza | 150 | 139/11 | 24.2±5.8 |  | 150 | 135/15 | 23.1±3.3 | None. |  |

**Supplementary Table 1 continued.**

| Studies | Disease | Lianhua Qingwen group | | |  | Conventional drug group | | | Adverse reactions | Dispose or outcome of adverse reactions |
| --- | --- | --- | --- | --- | --- | --- | --- | --- | --- | --- |
|  |  | S | M/F | Age |  | S | M/F | Age |  |  |
| Chen 2014a | Influenza | 60 | — | — |  | 60 | — | — | Gastrointestinal system damage: Diarrhea, Gastrointestinal distress. | Healed spontaneously without any treatment |
|  |  |  |  |  |  |  |  |  | Skin and its appendage damage: Rash. |  |
| Chen 2014b | Influenza | 40 | 27/13 | 30.4±10.0 |  | 30 | 26/4 | 30.1±10.5 | None. |  |
| Cheng 2014 | Respiratory tract infection | 75 | 40/35 | 14-39 |  | 75 | 39/36 | 15-60 | Gastrointestinal system damage: Gastrointestinal distress, Nausea, Vomiting. |  |
|  |  |  |  |  |  |  |  |  | Skin and its appendage damage: Rash |  |
| Dai 2014 | Respiratory tract infection | 35 | 19/16 | 19-55 |  | 35 | 18/17 | 18-53 | None. |  |
| Dai et al.,2014 | Influenza | 412 | 294/118 | — |  | 381 | 277/104 | — | Gastrointestinal system damage: Abdominal pain, Dyspepsia, Nausea, Vomiting. |  |
|  |  |  |  |  |  |  |  |  | Nervous system damage: Dizziness, headache. |  |
|  |  |  |  |  |  |  |  |  | Respiratory system damage: Cough. |  |
| Ding et al.,2014 | Mumps | 35 | 20/15 | — |  | 37 | 21/16 | — | None. |  |
| Dong 2014 | Common pneumonia | 40 | 21/19 | 71.64±11.73 |  | 42 | 24/18 | 72.58±10.69 | None. |  |
| Dong et al.,2014 | Chronic obstructive pulmonary disease | 30 | 27/3 | 69.34±11.64 |  | 30 | 28/2 | 22.14±5.70 | None. |  |

**Supplementary Table 1 continued.**

| Studies | Disease | Lianhua Qingwen group | | |  | Conventional drug group | | | Adverse reactions | Dispose or outcome of adverse reactions |
| --- | --- | --- | --- | --- | --- | --- | --- | --- | --- | --- |
|  |  | S | M/F | Age |  | S | M/F | Age |  |  |
| Gao 2014 | Common pneumonia | 60 | 34/26 | 48.2±6.7 |  | 60 | 33/27 | 51.3±4.8 | Gastrointestinal system damage: Gastrointestinal distress. |  |
|  |  |  |  |  |  |  |  |  | Skin and its appendage damage: Rash. |  |
|  |  |  |  |  |  |  |  |  | Abnormal white blood cells and reticuloendothelial system: Leukopenia. |  |
| He 2014 | Viral influenza | 57 | 31/26 | 42.3±5.5 |  | 56 | 33/23 | 41.9±5.8 | Gastrointestinal system damage: Gastrointestinal distress. |  |
| Jiang et al.,2014a | Common pneumonia | 60 | 32/28 | — |  | 60 | 38/22 | — | Gastrointestinal system damage: Dyspepsia, Nausea, Vomiting. |  |
|  |  |  |  |  |  |  |  |  | Skin and its appendage damage: Rash. |  |
|  |  |  |  |  |  |  |  |  | Hepatobiliary system damage: Hepatic function abnormal. |  |
|  |  |  |  |  |  |  |  |  | Other symptoms. |  |
| Jiang et al.,2014b | Respiratory tract infection | 182 | 97/85 | 42.38±12.94 |  | 182 | 101/81 | 44.46±12.62 | Abnormal white blood cells and reticuloendothelial system: Leukopenia. |  |
| Li et al.,2014a | Respiratory tract infection | 60 | 36/24 | 35.6±8.13 |  | 60 | 32/28 | 36.2±5.39 | None. |  |
| Li et al.,2014b | Respiratory tract infection | 30 | — | — |  | 30 | — | — | Only the number of adverse reactions was reported. | Improved with taking medication after meals |
|  |  |  |  |  |  |  |  |  | Gastrointestinal system damage: Abdominal discomfort, Diarrhea, Nausea. |  |
| Li 2014 | Influenza | 56 | 32/24 | 45.71±4.28 |  | 51 | 30/21 | 46.22±4.78 | Gastrointestinal system damage: Abdominal distension, Diarrhea. |  |
| Mei 2014 | Hoarseness disease | 30 | 9/21 | 38.16±10.31 |  | 30 | 12/18 | 36.26±10.39 | Gastrointestinal system damage: Diarrhea. | Healed spontaneously without any treatment |

**Supplementary Table 1 continued.**

| Studies | Disease | Lianhua Qingwen group | | |  | Conventional drug group | | | Adverse reactions | Dispose or outcome of adverse reactions |
| --- | --- | --- | --- | --- | --- | --- | --- | --- | --- | --- |
|  |  | S | M/F | Age |  | S | M/F | Age |  |  |
| Meng 2014 | Rheum | 50 | — | — |  | 50 | — | — | None. |  |
| Qin 2014 | Herpes zoster | 72 | 35/37 | 49.3±13.3 |  | 73 | 36/37 | 51.1±11.5 | Gastrointestinal system damage: Gastrointestinal distress. |  |
|  |  |  |  |  |  |  |  |  | Nervous system damage: Legacy neuralgia. |  |
| Shi 2014 | Common pneumonia | 34 | 16/18 | 70.5(61-75) |  | 34 | 19/15 | 69.5(59-74) | Gastrointestinal system damage: Loss of appetite. |  |
|  |  |  |  |  |  |  |  |  | Nervous system damage: Dizziness, headache. |  |
|  |  |  |  |  |  |  |  |  | Skin and its appendage damage: Itchy skin. |  |
| Sun 2014 | Influenza A (H1N1) | 15 | 9/6 | 27.8±1.6 |  | 15 | 8/7 | 26.7±1.7 | Gastrointestinal system damage: Nausea. | Recovered after symptomatic treatment |
| Wei et al.,2014 | Respiratory tract infection | 101 | 50/51 | 40.5±12.5 |  | 102 | 52/50 | 39.5±13.5 | Gastrointestinal system damage. | Not dispose adverse reactions |
| Wu et al.,2014 | Respiratory tract infection | 27 | 14/13 | 35.7±11.3 |  | 27 | 15/12 | 36.3±10.2 | Gastrointestinal system damage. |  |
| Wu 2014 | Respiratory tract infection | 72 | 34/38 | 48.5±10.9 |  | 65 | 34/31 | 49.0±7.5 | Gastrointestinal system damage: Diarrhea, Nausea, Vomiting.  Skin and its appendage damage: Itchy skin. | Recovered after symptomatic treatment |
|  |  |  |  |  |  |  |  |  |  |  |

**Supplementary Table 1 continued.**

| Studies | Disease | Lianhua Qingwen group | | |  | Conventional drug group | | | Adverse reactions | Dispose or outcome of adverse reactions |
| --- | --- | --- | --- | --- | --- | --- | --- | --- | --- | --- |
|  |  | S | M/F | Age |  | S | M/F | Age |  |  |
| Yan et al.,2014 | Rheum | 437 | 213/224 | 46.5(7-86) |  | 428 | 205/223 | 45.5(6-83) | Gastrointestinal system damage: Gastrointestinal distress. |  |
|  |  |  |  |  |  |  |  |  | Nervous system damage: Drowsiness. |  |
|  |  |  |  |  |  |  |  |  | Skin and its appendage damage: Rash. |  |
| Yuan 2014 | Viral influenza | 70 | 37/33 | 43.8±7.2 |  | 70 | 38/32 | 42.6±6.9 | Gastrointestinal system damage: Gastrointestinal distress. | Improved with taking medication after meals |
| Zhai 2014 | Respiratory tract infection | 80 | 44/36 | 40(18-62) |  | 80 | 48/32 | 39(19-59) | None. |  |
| Zhang 2014 | Influenza | 50 | 28/22 | 21.5±3.5 |  | 50 | 26/24 | 21.5±3.5 | Gastrointestinal system damage: Diarrhea, Abdominal distension, Gastrointestinal distress. |  |
| Zhou 2014 | Common pneumonia | 30 | 18/12 | 77.2±5.9 |  | 30 | 20/10 | 76.5±5.2 | Gastrointestinal system damage: Nausea, Vomiting. |  |
| Chen et al.,2013a | Common pneumonia | 76 | 44/32 | — |  | 76 | 42/34 | — | Gastrointestinal system damage: Diarrhea, Nausea. | Healed spontaneously after stopping the medication |
|  |  |  |  |  |  |  |  |  | Skin and its appendage damage: Rash. |  |
| Chen et al.,2013a | Respiratory tract infection | 40 | 22/18 | 40(18-62) |  | 40 | 24/16 | 39(19-59) | None. |  |
| Chen 2013 | Influenza | 140 | — | — |  | 130 | — | — | Gastrointestinal system damage: Diarrhea, Abdominal distension, Gastrointestinal distress. | Healed spontaneously after stopping the medication |

**Supplementary Table 1 continued.**

| Studies | Disease | Lianhua Qingwen group | | |  | Conventional drug group | | | Adverse reactions | Dispose or outcome of adverse reactions |
| --- | --- | --- | --- | --- | --- | --- | --- | --- | --- | --- |
|  |  | S | M/F | Age |  | S | M/F | Age |  |  |
| Gao et al.,2013 | Lung cancer | 15 | — | — |  | 15 | — | — | Gastrointestinal system damage: Diarrhea, Loss of appetite. | Improved spontaneously without any treatment |
| Gong 2013 | Rheum | 30 | — | — |  | 30 | — | — | Gastrointestinal system damage: Diarrhea, Nausea, Gastrointestinal distress. |  |
|  |  |  |  |  |  |  |  |  | Skin and its appendage damage: Rash. |  |
| He et al. | Herpes zoster | 50 | — | — |  | 50 | — | — | Only the number of adverse reactions was reported. |  |
| Jiang 2013 | Hand-foot- mouth disease | 24 | 7/17 | 4.5 |  | 22 | 8/14 | 4.8 | None. |  |
| Ju et al.,2013 | Chronic obstructive pulmonary disease | 20 | 14/6 | 68.87±6.51 |  | 20 | 15/5 | 65.91±6.55 | None. |  |
| Pang 2013 | Viral pharyngitis | 68 | 36/32 | 9.16±0.98 |  | 68 | 39/29 | 9.20±1.08 | Gastrointestinal system damage: Diarrhea, Nausea. | Not dispose adverse reactions |
| Peng 2013 | Common pneumonia | 30 | 16/14 | 33.6±11.2 |  | 30 | 15/15 | 32.8±10.1 | Only the number of adverse reactions was reported. |  |
|  |  |  |  |  |  |  |  |  | Gastrointestinal system damage: Diarrhea, Gastrointestinal distress, Loose stool. |  |
| Qu et al.,2013 | Respiratory tract infection | 78 | 41/37 | 21.4±3.2 |  | 88 | 43/35 | 21.1±3.3 | Gastrointestinal system damage: Nausea. | Improved after symptomatic treatment |

**Supplementary Table 1 continued.**

| Studies | Disease | Lianhua Qingwen group | | |  | Conventional drug group | | | Adverse reactions | Dispose or outcome of adverse reactions |
| --- | --- | --- | --- | --- | --- | --- | --- | --- | --- | --- |
|  |  | S | M/F | Age |  | S | M/F | Age |  |  |
| Shang et al.,2013a | Influenza A (H1N1) | 50 | 26/24 | 22.8±2.6 |  | 50 | 25/25 | 23.4±2.5 | Gastrointestinal system damage: Diarrhea, Nausea, Vomiting. |  |
|  |  |  |  |  |  |  |  |  | Nervous system damage. |  |
| Sheng et al.,2013b | Hand-foot- mouth disease | 186 | 91/95 | 4.5 |  | 183 | 90/93 | 3.8 | None. |  |
| Wang et al.,2013 | Exogenous fever caused by AIDS | 60 | 37/23 | 18-59 |  | 60 | 34/26 | 18-61 | Gastrointestinal system damage: Diarrhea, Nausea, Gastrointestinal distress. |  |
|  |  |  |  |  |  |  |  |  | Skin and its appendage damage: Rash. |  |
| Xie 2013 | Herpes simplex virus keratitis | 30 | 12/18 | 34.33(13-64) |  | 30 | 14/16 | 30.60(14-65) | Other symptoms: Resurgence of disease. |  |
| Xin 2013 | Respiratory tract infection | 86 | 42/44 | 19-58 |  | 80 | 83/42 | 17-57 | None. |  |
| Xu 2013 | Respiratory tract infection | 40 | 19/21 | 37.63±1.38 |  | 40 | 21/19 | 37.62±1.37 | None. |  |
| Ye et al.,2013 | Influenza | 32 | — | — |  | 32 | — | — | Gastrointestinal system damage: Gastrointestinal distress. |  |
| Deng et al.,2012 | Tonsillitis | 50 | 26/24 | 18-52 |  | 50 | 29/21 | 19-50 | None. |  |
| Duan 2012 | Influenza A (H1N1) Influenza | 8 | 4/4 | 38.5±24.90 |  | 19 | 8/11 | 37.5±17.90 | None. |  |
| Lin et al.,2012 | Hand-foot- mouth disease | 63 | 40/23 | 2.70(1-5) |  | 67 | 39/28 | 2.68(1-5) | Gastrointestinal system damage: Diarrhea. | Improved after symptomatic treatment |

**Supplementary Table 1 continued.**

| Studies | Disease | Lianhua Qingwen group | | |  | Conventional drug group | | | Adverse reactions | Dispose or outcome of adverse reactions |
| --- | --- | --- | --- | --- | --- | --- | --- | --- | --- | --- |
|  |  | S | M/F | Age |  | S | M/F | Age |  |  |
| Liu 2012 | Respiratory tract infection | 109 | — | — |  | 109 | — | — | Gastrointestinal system damage: Diarrhea, Abdominal distension, Gastrointestinal distress. |  |
| Lu et al.,2012 | Herpes zoster | 26 | 11/15 | 56. 4 ± 4. 7 |  | 24 | 10/14 | 57.1±5.2 | Nervous system damage: Legacy neuralgia. |  |
|  |  |  |  |  |  |  |  |  | Other symptoms. |  |
| Tan et al.,2012 | Respiratory tract infection | 29 | 15/14 | 36.85±11.32 |  | 29 | 16/13 | 35.24±10.18 | None. |  |
| Xu et al.,2012 | Stroke | 24 | 16/8 | 65(38-79) |  | 24 | 16/8 | 67(40-81) | None. |  |
| Yang 2012 | Respiratory tract infection | 28 | 10/18 | 56.75±10.41 |  | 28 | 13/15 | 56.57±6.70 | Gastrointestinal system damage: Gastrointestinal distress. | Healed spontaneously after stopping the medication |
| Yang et al.,2012 | Influenza | 58 | — | — |  | 58 | — | — | Only the number of adverse reactions was reported. |  |
|  |  |  |  |  |  |  |  |  | Gastrointestinal system damage: Diarrhea, Nausea, Gastrointestinal distress. |  |
|  |  |  |  |  |  |  |  |  | Skin and its appendage damage: Rash. |  |
| Yao 2012 | Rheum | 60 | — | — |  | 64 | — | — | None. |  |
| Zhang et al.,2012 | Influenza A (H1N1) | 56 | 30/26 | 24.20±10.30 |  | 56 | 31/25 | 22.30±11.60 | Gastrointestinal system damage: Diarrhea, Nausea, Vomiting. |  |
|  |  |  |  |  |  |  |  |  | Nervous system damage. |  |
| Duan et al.,2011 | Influenza A (H1N1) | 122 | 64/58 | 21.5±5.9 |  | 122 | 63/59 | 21.4±3.9 | Only the number of adverse reactions was reported. | Not dispose adverse reactions |

**Supplementary Table 1 continued.**

| Studies | Disease | Lianhua Qingwen group | | |  | Conventional drug group | | | Adverse reactions | Dispose or outcome of adverse reactions |
| --- | --- | --- | --- | --- | --- | --- | --- | --- | --- | --- |
|  |  | S | M/F | Age |  | S | M/F | Age |  |  |
| Cai et al.,2011 | Hand-foot- mouth disease | 28 | 25/13 | 2.46±0.22 |  | 36 | 23/13 | 2.39±0.31 | Gastrointestinal system damage: Diarrhea, Nausea, Vomiting. |  |
|  |  |  |  |  |  |  |  |  | Heart rate and arrhythmia: Arrhythmia. |  |
|  |  |  |  |  |  |  |  |  | Other symptoms: Hemoglobin drops. |  |
| Cui et al.,2011 | Influenza | 100 | — | — |  | 100 | — | — | Gastrointestinal system damage: Diarrhea. |  |
|  |  |  |  |  |  |  |  |  | Secondary infection: Sinusitis, Tonsillitis. |  |
| Hu 2011 | Respiratory tract infection | 100 | 55/45 | — |  | 100 | 53/47 | — | Gastrointestinal system damage. | Not dispose adverse reactions |
| Luo 2011a | Viral influenza | 40 | 22/18 | 32.9±12.6 |  | 40 | 26/14 | 33.8±11.4 | Gastrointestinal system damage: Gastrointestinal distress. | Improved with taking medication after meals |
| Luo 2011b | Influenza | 35 | 17/18 | 23.54(18-41) |  | 35 | 15/20 | 24.39(19-38) | Gastrointestinal system damage: Nausea, Vomiting. |  |
| Wang et al.,2011 | Influenza A (H1N1) | 30 | — | — |  | 63 | — | — | None. |  |
| Xun et al.,2011 | Respiratory tract infection | 50 | 29/21 | — |  | 50 | 27/23 | — | Gastrointestinal system damage: Diarrhea, Abdominal distension. | Improved with taking medication after meals |
| Yang et al.,2011 | Respiratory tract infection | 30 | 19/11 | 45.5±5.9 |  | 30 | 19/11 | 48.6±8.1 | Gastrointestinal system damage: Dry mouth, Loss of appetite.  Nervous system damage: Dizziness. | Healed spontaneously after stopping the medication |
|  |  |  |  |  |  |  |  |  |  |  |
| Yang 2011 | Respiratory tract infection | 57 | 32/25 | 18-62 |  | 56 | 32/23 | 16-58 | None. |  |
| Yu et al.,2011 | Influenza A (H1N1) | 28 | — | — |  | 128 | 101/21 | 23.3±2.3 | None. |  |

**Supplementary Table 1 continued.**

| Studies | Disease | Lianhua Qingwen group | | |  | Conventional drug group | | | Adverse reactions | Dispose or outcome of adverse reactions |
| --- | --- | --- | --- | --- | --- | --- | --- | --- | --- | --- |
|  |  | S | M/F | Age |  | S | M/F | Age |  |  |
| Zhang et al.,2011 | Hand-foot- mouth disease | 46 | 14/32 | 4.4±0.4 |  | 41 | 13/28 | 4.9±0.5 | None. |  |
| Zhou 2011 | Influenza | 62 | 37/25 | 17-55 |  | 62 | 34/28 | 16-53 | None. |  |
| Lai et al.,2010 | Influenza A (H1N1) | 50 | 28/22 | 23(11-68) |  | 50 | 30/20 | 23.7(13-69) | None. |  |
| Liu et al.,2010 | Influenza A (H1N1) | 64 | 34/30 | 19.8±3.7 |  | 60 | 35/25 | 19.6±1.4 | None. |  |
| Ma et al.,2010 | Influenza A (H1N1) | 60 | 42/18 | 23.6±8.8 |  | 74 | 47/27 | 23.2±8.7 | Only the number of adverse reactions was reported. |  |
| Ouyang et al.,2010 | Influenza A (H1N1) | 116 | 59/57 | 19.23±10.44 |  | 29 | 16/13 | 19.69±9.91 | Secondary infection: Pulmonary infection. |  |
| Wei et al.,2010 | Influenza A (H1N1) | 30 | — | — |  | 16 | — | — | Gastrointestinal system damage: Diarrhea, Nausea. |  |
|  |  |  |  |  |  |  |  |  | Nervous system damage. |  |
| Zhang et al.,2010a | Pulmonary infection | 30 | 11/19 | — |  | 30 | 21/9 | — | Gastrointestinal system damage: Gastrointestinal distress. |  |
|  |  |  |  |  |  |  |  |  | Skin and its appendage damage: Rash. |  |
|  |  |  |  |  |  |  |  |  | Abnormal white blood cells and reticuloendothelial system: Leukopenia. |  |
| Zhang et al.,2010b | Hand-foot- mouth disease | 68 | 49/19 | 3.4±0.4 |  | 68 | 38±30 | 2.9±0.5 | None. |  |
| Zheng 2010 | Influenza | 65 | 30/35 | — |  | 65 | 28/37 | — | Gastrointestinal system damage: Gastrointestinal distress, Diarrhea. |  |
|  |  |  |  |  |  |  |  |  | Skin and its appendage damage: Rash. |  |
| Li et al.,2009 | Influenza A (H1N1) | 25 | 11/14 | 19 |  | 25 | 9/16 | 18 | Gastrointestinal system damage: Diarrhea, Nausea, Vomiting. |  |

**Supplementary Table 1 continued.**

| Studies | Disease | Lianhua Qingwen group | | |  | Conventional drug group | | | Adverse reactions | Dispose or outcome of adverse reactions |
| --- | --- | --- | --- | --- | --- | --- | --- | --- | --- | --- |
|  |  | S | M/F | Age |  | S | M/F | Age |  |  |
| Li et al.,2009 | Tonsillitis | 38 | 23/15 | 0.8-12 |  | 35 | 21/14 | 0.7-11 | None. |  |
| Hu et al.,2008 | Respiratory tract infection | 102 | 54/48 | 32.4±10.5 |  | 104 | 61/43 | 33.8±11.7 | Gastrointestinal system damage: Gastrointestinal symptoms. | Not dispose adverse reactions |
| Wang et al.,2008a | Influenza | 80 | 36/44 | — |  | 80 | 46/34 | — | Gastrointestinal system damage: Gastrointestinal distress, Diarrhea. |  |
|  |  |  |  |  |  |  |  |  | Skin and its appendage damage: Rash. |  |
| Wang et al.,2008b | Viral influenza | 100 | 58/42 | — |  | 100 | 53/47 | — | Gastrointestinal system damage: Gastrointestinal distress. | Improved with taking medication after meals |
| Wang et al.,2008c | Respiratory tract infection | 25 | 15/10 | — |  | 25 | 17/8 | — | Gastrointestinal system damage: Gastrointestinal distress. |  |
|  |  |  |  |  |  |  |  |  | Skin and its appendage damage: Rash. |  |
|  |  |  |  |  |  |  |  |  | Abnormal white blood cells and reticuloendothelial system: Leukopenia. |  |
| Wu et al.,2006 | Chronic cor pulmonale | 29 | 19/10 | 65.19±6.39 |  | 28 | 17/11 | 64.86± 6.28 | None. |  |
| Zuo et al.,2006 | Respiratory tract infection | 120 | 78/42 | 37.96±11.25 |  | 100 | 58/42 | 39.78±13.78 | Only the number of adverse reactions was reported. |  |
|  |  |  |  |  |  |  |  |  | Gastrointestinal system damage: Abdominal pain, Nausea, Vomiting. |  |
| Yang et al.,2005a | Influenza | 116 | — | — |  | 117 | — | — | Only the number of adverse reactions was reported |  |
| Yang et al., 2005b | Influenza | 197 | 134/63 | 30.4±10.0 |  | 67 | 42/25 | 30.1±10.5 | Gastrointestinal system damage: Diarrhea. |  |

S: Sample size, M: Male, F: Female.

**References**

Bai, R., Yang, C. (2018). Effects of thymopentin combined with lianhua qingwen capsule on aecopd patients and its influence on immune function. *Laboratory Medicine and Clinic*. 15(05), 592-595. doi: CNKI:SUN:JYYL.0.2018-05-007

Bi, Z. S., Yan, J. H., Wang, Z. K., Liang, B. (2015). Effects of lianhuagingwen capsules on patients with chronic obstructive pulmonary disease and changes in tnf-a, il-8 and il-7. *Chinese Journal of Difficult and Complicated Cases*. 14(9), 891-894. doi: CNKI:SUN:YNBZ.0.2015-09-004

Cai, L. L., Lin, T., Zhong, G.H., Zheng, P. M. (2020). Clinical observation of lianhua qingwen granules combined with broncho-vaxom treating recurrent respiratory tract infection in children aged 0-5 and its effect on inflammatory factors. *Chinese Archives of Traditional Chinese Medicine*. 38(08), 195-199. doi: 10.13193/j.issn.1673-7717.2020.08.047

Cai, S. Y., Yan, Z. G., Gang, X. H. (2011). Clinical observation on treating hand, foot and mouth disease by using lianhuaqingwen granules combined with ribavirin. *Journal of Medical Forum*. 32(16), 76-78. doi: CNKI:SUN:HYYX.0.2011-16-028

Chao, X. R., Xie, Y. Y., Li, Q. D., Shi. R. X. (2020). Clinical study on lianhua qingwen capsules combined with ganciclovir in treatment of herpes simplex keratitis. *Drugs & Clinic*. 35(10), 1990-1993. doi: CNKI:SUN:GWZW.0.2020-10-012

Chen, B., Yao, Y. W., Wang, H. Q. (2013a). Efficacy of lianhua qingwen granule combined with moxifloxacin tablets in the treatment of elderly community-acquired pneumonia observed. *Jilin Medical Journal*. 34(1), 79-80. doi: CNKI:SUN:JLYX.0.2013-01-050

Chen, C. (2014a). Clinical experience of lianhua qingwen capsule in the treatment of influenza. *Modern Diagnosis and Treatment*. 25(02), 278-279. doi: CNKI:SUN:XDZD.0.2014-02-030

Chen, H. (2017). *Clinical observation of lianhua qingwen capsule combined with acyclovir in the treatment of middle-aged and elderly patients with herpes zoster*. Paper presented at the China Association of Traditional Chinese Medicine: Chinese Association of Traditional Chinese Medicine Branch.

Chen, H. W. (2016). *Clinical observation of compound qinghua granules in the treatment of viral upper respiratory tract infection with wind-heat invading lung syndrome*. Master degree, Fujian University of Traditional Chinese Medicine.

Chen, L. Q., Chen, W. H. (2020a). Lianhua qingwen granules for mycoplasma pneumoniae in children. *China Health Standard Management*. 11(11), 104-106. doi: CNKI:SUN:WSBZ.0.2020-11-042

Chen, S. F. (2014b). Observation on curative effect of lianhuaqingwen capsule combined cefradine on influenza. *Chinese Journal of Difficult and Complicated Cases*. 2.

Chen, S. Y., Li, Z. D. (2013). Effect of lianhua qingwen capsule on acute upper respiratory tract infection. *World Health Digest.* (9), 100-101. doi: 10.3969/j.issn.1672-5085.2013.09.092

Chen, Y. L. (2013b). Clinical effect of lianhua qingwen capsule in the treatment of influenza. *Journal of Qiqihar Medical University*. 34(09), 1304. doi: CNKI:SUN:QQHB.0.2013-09-033

Chen, Y., Han, C. Q., Zhao, F. L., Fu, H. L, Xi, Y. F. (2020b). Effects of lianhua qingwen granules combined with azithromycin on children with mycoplasma pneumoniae pneumonia. *World Chinese Medicine*. 15(1), 76-80.

Cheng, C. Y. (2018). Efficacy and safety analysis of lianhua qingwen capsule in the treatment of viral cold. *Journal of North Pharmacy*. 15(03), 163. doi: CNKI:SUN:BFYX.0.2018-03-139

Cheng, X. (2014). Therapeutic effect of lianhua qingwen capsule on acute upper respiratory tract infection. *Chinese Journal of Clinical Rational Drug Use*. 7(04), 39-40. doi: 10.15887/j.cnki.13-1389/r.2014.04.011

Cui, M., Pan, X. Q. (2011). Lianhua qingwen capsule treated 100 cases of influenza. *Zhejiang Journal of Traditional Chinese Medicine*. 46(05), 333. doi: CNKI:SUN:ZJZZ.0.2011-05-018

Dai, Y. M. (2014). Observation on the therapeutic effect of respiratory tract infection in lianhua qingwen capsule treatment. *Medical Aesthetics and Cosmetology*. 12, 300-301. doi: 10.14010/j.cnki.wjyx.2014.08.002

Dai, Y. L., Bai, H. Y., Liu, J. C. (2014). Efficacy and safety of lianhuaqingwen capsules in treatment of influenza. 25(08), 800-802, 807.

Deng, W. H., Pang, L. L. (2012). Effect of lianhua qingwen capsule on acute tonsillitis. *Chinese Journal of Clinical Rational Drug Use*. 5(25), 78. doi: 10.15887/j.cnki.13-1389/r.2012.25.014

Ding, G. F., Liu, N. (2014). *Therapeutic effect of lianhuagingwen capsule combined with ribavirin on epidemic parotitis*. Paper presented at the Clinical research of collateral disease.

Dong, F. L. (2014). Observation of curative effect of lianhua qingwen granules combined ceftriaxone sodium for injection on the treatment of senile bacterial pneumonia. *Hebei Journal of Traditional Chinese Medicine*. 36(10), 1530-1531. doi: CNKI:SUN:HBZY.0.2014-10-051

Dong, G. B. (2015). The clinical effectiveness of biqing kiling dispersible tablets and lianhua qingwen capsule in the treatment of viral cold and fever. *World Latest Medicne Information*. 15(55), 70-71. doi: CNKI:SUN:WMIA.0.2015-55-060

Dong, L. X., Jing W., Gong, Y., Chen, X. D. (2014). Clinical effect of lianhua qingwen capsules on patients with acute exacerbation of chronic obstructive pulmonary disease and its mechanism. *Chinese Traditional Patent Medicine*. 36(05), 926-929. doi: CNKI:SUN:ZCYA.0.2014-05-009

Du, X B., Chen, Q. B., Xiao, Y., Zhang, X. Q., Xia, J. H., Liu, S. (2019). An open-label, multi-center, random-control clinical study of xiangju capsules in treatment of influenza. *China Medicine*. 14(7), 981-983. doi: CNKI:SUN:ZGYG.0.2019-07-006

Du, S. J., Geng, S. H., Yuan, H. E., Mu, Z., Lin, Li X., Ji, S. Z. (2015). Hebei journal of traditional chinese medicine. *Lianhua Qingwen Granule in the Treatment of 43 Cases of Herpes Simplex Virus Keratitis*. 37(07), 1064-1065. doi: CNKI:SUN:HBZY.0.2015-07-038

Duan, L. (2012). Therapeutic effective observation on 27 patients with influenza a. *Chongqing Medicine*. 41(13), 1281-1282. doi: CNKI:SUN:CQYX.0.2012-13-015

Duan, Z. P., Jia, Z. H., Zhang, J., Liu, S., Chen, Y., Liang, L. C., et al. (2011). Natural herbal medicine lianhuaqingwen capsule anti-influenza a (h1n1) trial: a randomized, double blind, positive controlled clinical trial. *Chin Med J (Engl)*. 124(18), 2925-2933.

Fang, F., Yang, L., Qin, S. C., Jiao. R. (2020). Clinical efficacy of traditional chinese medicine lianhua qingwen granules in 42 suspected cases of children with corona virus disease 2019. *Chinese Journal of New Drugs*. 29(24), 2809-2812. doi: CNKI:SUN:ZXYZ.0.2020-24-008

Fang, H. Z. (2015). Clinical observation on the treatment of 35 cases of herpes zoster with lianhua qingwen capsule and vinegar. *Practical Clinical Journal of Integrated Traditional Chinese and Western Medicine*. 37(07), 1064-1065. doi: 10.13638/j.issn.1671-4040.2015.02.023

Fang, K., Wei, T., Zhou, W. X., Zhou, Z. R., Liu, J., Zhuang, J., et al. (2016). Effect analysis of lianhua qingwen capsule on acute upper respiratory tract infection. *Inner Mongolia Journal of Traditional Chinese Medicine*. 35(15), 14. doi: 10.16040/j.cnki.cn15-1101.2016.15.013

Feng, X. B., Song, G. L. (2016). Clinical observation of lianhua qingwen granules combined with antibiotics in treating acute pharyngitis. *Practical Journal of Cardiac Cerebral Pneumal and Vascular Disease*. 24(0), 197.

Feng, X. Y. (2015). Clinical observation on 50 cases of malaria treated by lianhua qingwen capsule combined with artesunate injection. *Hebei Journal of Traditional Chinese Medicine*. 37(01), 106-107. doi: 10.13638/j.issn.1671-4040.2015.02.023

Feng, X., Cheng, Y. L. (2019). Efficacy evaluation of combined chinese and western medicine in acute upper respiratory tract infection. *Chinese Journal of School Doctor*. 33(06), 472-473. doi: CNKI:SUN:XIYI.0.2019-06-031

Fu, T. (2020). Clinical observation on prevention and control of influenza. *Friends of the Health.* (12), 33.

Gao, S. G. (2014). Observation on the efficacy and safety of lianhuaqingwen granule in adjuvant treatment of pneumonia. *Journal of Modern Medicine & Health*. 30(12), 1876-1877. doi: CNKI:SUN:XYWS.0.2014-12-063

Gao, Y. Z, Zhao, N., Hu, B. (2013). Clinical effect of lianhua qingwen capsule on lung cancer with fever. *Clin. Med.* 33(09), 122-123. doi: CNKI:SUN:EBED.0.2013-09-074

Gong, S. D. (2013). Analysis on the effect of lianhua qingwen capsule on epidemic wind-heat cold. *Healthmust-Readmagazine*. 12(4), 83.

Guo, W. M. (2015). Clinical observation of lianhua gingwen capsule joint with jingqiaomai tablet in treatment of influenza a (hin1). *Journal of Chengdu Medical College*. 10(3), 357-359. doi: CNKI:SUN:CDYU.0.2015-03-027

Guo, Y. Z., Wei, G. Y. (2020). Clinical comparative study of lianhua qingwen granule and qingkailing granule in the treatment of influenza. *China Health Care & Nutrition*. 30(22), 117-118.

Han, C. Y. (2016). Lianhua qingwen granule combined with ribavirin in treatment of 60 cases of hand, foot and mouth disease in children. *Chinese Journal of Ethnomedicine and Ethnopharmacy*. 26(11), 108-110. doi: CNKI:SUN:MZMJ.0.2017-11-038

Han, Z. P. (2019). Clinical effect and adverse reaction of oseltamivir phosphate in the treatment of influenza. *Guide of China Medicine*. 17(14), 169-170. doi: 10.15912/j.cnki.gocm.2019.14.124

Hao, G. R. (2019). Clinical effect analysis of oseltamivir combined with lianhua qingwen capsule for treatment of influenza a (h1n1). *Guide of China Medicine*. 17(34), 103. doi: 10.15912/j.cnki.gocm.2019.34.076

He, C. H. (2014). Efficacy of lianhua qingwen capsule in the treatment of viral cold in community. *For all Health*. 8(17), 261-262. doi: CNKI:SUN:XYWS.0.2014-12-063

He, W. P., He, D. Q., He, Y. H. (2013). Experience of lianhua qingwen capsule combined with acyclovir in treating zonal scar rash. *Henan Journal of Surgery*. 19(06), 112-113. doi: CNKI:SUN:HLWK.0.2013-06-084

He, W. Q. (2015). Efficacy and safety analysis of lianhua qingwen capsule in the treatment of viral cold. *Modern Diagnosis and Treatment*. 26(17), 3876-3878. doi: CNKI:SUN:XDZD.0.2015-17-026

Hu, G. F. (2011). Evaluation of the effect of lianhuaqingwen capsulein treatment of acute upper respiratory tract infection. *Practical Journal of Cardiac Cerebral Pneumal and Vascular Disease*. 19(05), 832-833. doi: CNKI:SUN:XDZD.0.2015-17-026

Hu, K., Guan, W. J., Bi, Y., Zhang, W., Li, L., Zhang, B., et al. (2021). Efficacy and safety of lianhuaqingwen capsules, a repurposed chinese herb, in patients with coronavirus disease 2019: a multicenter, prospective, randomized controlled trial. *Phytomedicine*. 85, 153242. doi: 10.1016/j.phymed.2020.153242

Hu, K., Jiang, Y., Shi, M. J., Hu, C. H., Liu, Z. L., Wan, Z. H. (2008). Lianhua qingwen capsule was used to treat 102 cases of acute upper respiratory tract infection. *Herald of Medicine.* (11), 1337-1340. doi: CNKI:SUN:YYDB.0.2008-11-020

Hu, Shu Yun. (2018). Clinical study on chaishi antipyretic particles in the treatment of influenza. *Chinese Community Doctors*. 34(04), 100-101. doi: CNKI:SUN:XCYS.0.2018-04-064

Hu, X. Q., Wan, Y., Lu, Q., Lei, X. Y., Yuan, L., Zeng, Y. P., et al. (2018). Clinical study of lianhuaqingwen capsules combined with cefuroxime in treatment of community acquired pneumonia. *Drugs & Clinic*. 33(12), 3216-3220. doi: CNKI:SUN:GWZW.0.2018-12-030

Hua, L., Liu, S. (2019). Clinical efficacy of lianhua qingwen granules combined with oseltamivir phosphate capsule in treatment of influenza. *China Medicine*. 14(8), 1155-1158.

Huang, A. J. (2017). Study on relieving effect of luofu mountain baicao oil on influenza symptoms. *Yiyao Qianyan*. 7(20), 349-350. doi: 10.3969/j.issn.2095-1752.2017.20.302

Huang, H., Luo, W. J. (2017a). Analysis of curative effect of lanqin oral liquid combined with lianhua qingwen granules on hand, foot and mouth disease. *Diet Health*. 4(13), 56-57. doi: 10.3969/j.issn.2095-8439.2017.13.063

Huang, K. (2018). Observation on the effect of lianhua qingwen capsule in the treatment of viral cold in community. *Health Guide.* (26), 292. doi: 10.3969/j.issn.1006-6845.2018.26.272

Huang, Z. H., Wang, X. H. (2017b). Effect analysis of lianhua qingwen granule combined with ribavirin aerosol in the treatment of hand, foot and mouth disease in children. *ournal of Diseases Monitor & Control*. 11(3), 231-232. doi: CNKI:SUN:JBJK.0.2017-03-036

Ji, H. (2019). Application of lianhua qingwen capsule in the treatment of students' acute tonsillitis. *World Latest Medicine Information*. 19(97), 13-14. doi: CNKI:SUN:JBJK.0.2017-03-036

Jian, Y. S. (2020). Clinical effect of lianhua qingwen granule in the treatment of influenza. *Healthmust-Readmagazine*(14), 31.

Jiang, L. Q. (2020). Observation on the curative effect of compound hibiscus tubi ointment combined with lianhua qingwen capsule in the treatment of epistaxis. *China Health Care & Nutrition*. 30(24), 250.

Jiang, S. (2013). Analysis of therapeutic effect of kangfuxin liquid and lianhuaqingwen capsule combined in treatment of hand foot and mouth disease. *China Health Industry*. 10(02), 75. doi: 10.16659/j.cnki.1672-5654.2013.02.023

Jiang, X., Wang, Z. P., Yu, T., Liang, Y. X., Li, Y., He, J. M., et al. (2014a). Clinical observation of lianhua qingwen capsule in treating community acquired pneumonia. *Chinese Journal of Clinical Rational Drug Use*. 7(10), 59-60. doi: 10.15887/j.cnki.13-1389/r.2014.10.072

Jiang, Y. D., Wu, C. F., Luo, Z. J, Guan, M. C., Jin, X. H. (2014b). The therapeutic efficacy of lianhua qingwen capsule combined with ribavirin aerosol in patients with acute upper respiratory tract infection. *China Medical Herald*. 11(23), 64-66, 70. doi: CNKI:SUN:YYCY.0.2014-23-022

Jin, X. F. (2016). Clinical view of lianhua qingwen capsule in the treatment of influenza. *Journal of New Chinese Medicine*. 48(06), 46-48. doi: 10.13457/j.cnki.jncm.2016.06.020

Ju, H. P., Jing, X. J., Wang, D. Y, Zhang, W. (2013). Clinical study of antiviral therapy in treatment of acute exacerbation of chronic obstructive pulmonary disease. *Journal of Modern Medicine & Health*. 29(03), 339-341. doi: CNKI:SUN:XYWS.0.2013-03-012

Kong, S. S. (2018). Effect evaluation of lianhua qingwen capsule in the treatment of influenza. *Medical Diet and Health.* (7), 156, 158.

Lai, F. X, Tan, F. Y., Wei. M. H. (2010). Clinical observation and nursing of treatment of influenza a (h1n1). *China & Foreign Medical Treatment*. 29(30), 100. doi: 10.16662/j.cnki.1674-0742.2010.30.022

Lei, X. (2020). Efficacy and safety of lianhua qingwen granule combined with oseltamivir in the treatment of influenza virus infection in children. *Drugs and Clinic*. 17(13), 62-64

Li, B. F., Zhang, C. Q., Fu, M., Bai. W., Liu, B. H., Li. S. S. (2009). Clinical study of lianhua qingwen capsule in the treatment of influenza a h1n1. *Journal of Medical Forum*. 30(23), 91-92. doi: CNKI:SUN:HYYX.0.2009-23-045

Li, B., Meng, Y. (2014c). Clinical observation of treating 60 cases of acute upper respiratory tract infection with lianhua qingwen granule. *Chinese Journal of Ethnomedicine and Ethnopharmacy*. 11(23), 64-66, 70. doi: CNKI:SUN:MZMJ.0.2014-17-029

Li, B., Sun, Y. S., Li, H. Y. (2020). Effect of lianhua qingwen granule on the expression of serum amyloid a and inflammatory factor in children with viral upper respiratory tract infection and fever. *Journal of Chinese Medicinal Materials*. 43(06), 1486-1489. doi: 10.13863/j.issn1001-4454.2020.06.039

Li, C. Z., Hou, J. Q., Lin, Y. Z. (2015). Observation of curative effect of lianhua qingwen capsule on acute upper respiratory tract infection. *Medicine and Society*. 28(0), 349

Li, G. (2016). Clinical observation of lianhua qingwen capsule combined with oseltamivir phosphate in the treatment of influenza. *Chinese Journal of Clinical Rational Drug Use*. 9(27), 137-138. doi: 10.15887/j.cnki.13-1389/r.2016.27.068

Li, H., Li, J. (2018b). Lianhua qingwen granule in the treatment of repeated respiratory infections in children and its effects on immune function. *Clinical Research and Practice*. 3(18), 133-134. doi: 10.19347/j.cnki.2096-1413.201818062

Li, K., Liu, Y. F. (2016). To explore the clinical effect of lianhua qingwen capsule in the treatment of viral cold. *China Health Care & Nutrition*. 26(9), 239.

Li, Q. (2018). Effect of lianhua qingwen capsule on acute upper respiratory tract infection. *Henan Medical Research*. 27(15), 2818-2819

Li, S. X., Chen, Y. S. (2018a). Observation on the curative effect of oseltamivir phosphate combined with lianhua qingwen granules in the treatment of influenza a. *Strait Pharmaceutical Journal*. 30(05), 134-135. doi: CNKI:SUN:HAIX.0.2018-05-057

Li, T. H. (2019). Clinical evaluation of lianhua qingwen capsule in treating acute upper respiratory tract infection. *Guide of China Medicine*. 17(12), 199-200. doi: 10.15912/j.cnki.gocm.2019.12.152

Li, W., Xi, H. J. (2014d). Clinical observation of lianhua qingwen capsule in treating acute upper respiratory tract infection. *Science & Technology Information*. 4(15), 377, 400. doi: CNKI:SUN:KJXX.0.2014-15-293

Li, W. J. (2020a). Analysis of curative effect of 60 cases of influenza treated with lianhua qingwen capsule. *Diet Health*. 7(18), 86.

Li, X. L. (2015a). Observation of curative effect of lianhuaqingwen particles combined with ribavirin and kangfuxinye in the treatment of herpetic stomatitis. *Chinese Journal of Clinical Rational Drug Use*. 8(05), 42-43. doi: 10.15887/j.cnki.13-1389/r.2015.05.020

Li, Y. N. (2020b). Observation of clinical efficacy of lianhua qingwen granule on patients with upper respiratory tract infection. *Chinese and Foreign Medical Research*. 18(36), 135-137. doi: 10.14033/j.cnki.cfmr.2020.36.054

Li, Y. P. (2015b). *Clinical observation of lianhua qingwen capsule combined with acyclovir in treating 72 cases of herpes zoster*. Paper presented at the Eleventh International Congress of Epidemiology.

Li, Y. Q. (2014). Observation on the curative effect of lianhua qingwen capsule in the treatment of influenza. *Contemporary Medical Symposium*. 12(10), 31-32. doi: CNKI:SUN:QYWA.0.2014-10-024

Li, X. J, Li, C. R. (2009). Clinical observation of lianhua qingwen granule in treating acute tonsillitis. *Chinese Journal of Clinical Rational Drug Use*. 2(09), 49. doi: CNKI:SUN:PLHY.0.2009-09-04

Liang, C.F. (2019). Clinical treatment of cold of heat dampness type treated by ephipuxialing decoction and lianhua qingwen granule. *Inner Mongolia Journal of Traditional Chinese Medicine*. 38(08), 14-15. doi: 10.16040/j.cnki.cn15-1101.2019.08.007

Liang, Z. S., Wang, Y.C. (2019). Clinical efficacy and safety of lianhua qingwen capsule combined with oseltamivir phosphate in the treatment of influenza. Chinese Journal of Clinical Rational Drug Use. 12(33), 92-93. doi: 10.15887/j.cnki.13-1389/r.2019.33.046

Liao, X. L. (2017). Clinical experience of lianhua qingwen capsule in the treatment of influenza. *Contemporary Medicine*. 23(10), 120-122.

Lin, H, X, You, C. F., Chu, J. Y. (2012). Clinical observation of combined application of lianhuaqingwen capsule in treatment of hand foot and mouth disease. *World Health Digest*. 9(14), 407-408. doi: 10.3969/j.issn.1672-5085.2012.14.386

Lin, L., Dai, F., Ren, G., Wei, J., Chen, Z., and Tang, X. (2020). Efficacy of lianhuaqingwen granules in the management of chronic rhinosinusitis without nasal polyps. *Am J Otolaryngol*. 41(1), 102311. doi: 10.1016/j.amjoto.2019.102311

Lin, L., Dai, F., Cheng, L. (2015). The treatment of lianhuaqing wen for uncomplicated acute bacterial rhinosinusitis *Chinese Journal of Otorhinolaryngology In Integrative Medicine*. 23(6), 414-419, 431. doi: 10.16542/j.cnki.issn.1007-4856.2015.06.004

Liu, C., Wang, H, A. (2017). Clinical study on lianhua qingwen capsules combined with cefoperazone sodium and sulbactam sodium in treatment of community acquired pneumonia. *Drugs & Clinic*. 32(7), 1251-1254. doi: CNKI:SUN:GWZW.0.2017-07-020

Liu, G. X, Zhang, Y. X., Yang, J.Q. (2010). A randomized controlled clinical study of lianhua qingwen capsule in the treatment of influenza a hin1. *Chinese Journal of Difficult and Complicated Cases*. 9(01), 14-16. doi: CNKI:SUN:YNBZ.0.2010-01-009

Liu, L., Shi, F., Tu, P., Chen, C., Zhang, M., Li, X., et al. (2021). Arbidol combined with the chinese medicine lianhuaqingwen capsule versus arbidol alone in the treatment of covid-19. *Medicine (Baltimore)*. 100(4), e24475. doi: 10.1097/MD.0000000000024475

Liu, L. (2018). Efficacy and safety analysis of lianhua qingwen granules in the treatment of viral cold. *Healthmust-Readmagazine*(28), 204.

Liu, S. S. (2020a). Effect of lianhua qingwen capsule combined with oseltamivir on the time of virus turning negative and inflammatory factors in adult patients with influenza a. *China Journal of Pharmaceutical Economics*. 15(11), 107-109. doi: CNKI:SUN:ZYWA.0.2020-11-027

Liu, X. J. (2020b). Observation of curative effect of lianhua qingwen granule combined with azithromycin sequential therapy on mycoplasma pneumoniae pneumonia in children. *Modern Diagnosis and Treatment*. 31(06), 856-857. doi: CNKI:SUN:XDZD.0.2020-06-007

Liu, X., Yang, S., Zhang, J. X., He, Q. (2015). *Observation on the curative effect of lianhua qingwen granules in adjuvant treatment of aecopd*. Paper presented at the China Journal of Traditional Chinese Medicine 2015/ Special Album.

Liu, Y. P. (2020c). Efficacy and safety of lotus qingwen capsule in the treatment of influenza patients. *World Latest Medicine Information*. 20(65), 1-2. doi: 10.3969/j.issn.1671-3141.2020.65.001

Liu, Y. Y. (2012). Observation on curative effect of lianhua qingwen capsule in treating acute upper respiratory tract infection. *Health Medicine Research and Practice*. 9(04), 44-45. doi: CNKI:SUN:GXBJ.0.2012-04-017

Liu. Z. Y. (2015). Effect analysis of lianhua qinwen capsule in the treatment of viral acute upper respiratory tract infection. *Contemporary Medical Symposium*. 13(21), 21-22. doi: CNKI:SUN:QYWA.0.2015-21-019

Liu. Z. N, Lu. H. W, Ceng, J. M, Cui, M. M, Wu, C. Y., Feng H. J., et al. (2020). Lianhua qingwen granule combined with oseltamivir phosphate in the treatment of infantile influenza a. *Inner Mongolia Journal of Traditional Chinese Medicine*. 39(01), 6-8. doi: 10.16040/j.cnki.cn15-1101.2020.01.003

Lu, L. J., Li, D. D., Liu, C., Wang, X., Chen, H. (2015). Clinical observation of qinzhiyuzhike decoction in treatment of cough with phlegmy heat intrin-

sic type. *Hubei Journal of Traditional Chinese Medicine*. 37(01), 3-4. doi: CNKI:SUN:HBZZ.0.2015-01-002

Lu, Y. Q., Wang, X. G. (2012). Clinical observation of lianhuaqingwen capsule combined with acyclovir in the treatment of herpes zoster. *Chinese Community Doctors*. 14(12), 143-144. doi: CNKI:SUN:ZGSQ.0.2012-12-132

Lu, Y., Shao, D. F., Sun, Y., Liu, Y., Fu, A. S., Li, L. Q. (2020). The efficacy of lianhuaqingwen capsule combined with levofloxacin in the treatment of community-acquired pneumonia and its influence on lcq score and inflammatory factors. *Chinese Journal of Difficult and Complicated Cases*. 19(11), 1152-1156. doi: CNKI:SUN:YNBZ.0.2020-11-017

Luo, C. (2011a). Clinical study of lianhua qingwen capsule in the treatment of viral cold. *China Modern Medicine*. 18(18), 137-138. doi: CNKI:SUN:ZGUD.0.2011-18-091

Luo, H. X. (2011b). Clinical observation of lianhua qingwen capsule in treating 35 cases of early influenza. *Chinese Journal of Traditional Medical Science and Technology*. 18(06), 517-518. doi: CNKI:SUN:TJYY.0.2011-06-048

Lv, H., Ouyang, Z. N., Wang, J., Yang, G. (2018). Clinical study on lianhua qingwen capsules combined with levofloxacisn in treatment of pneumonia. *Drugs & Clinic*. 33(9), 2294-2297. doi: CNKI:SUN:GWZW.0.2018-09-031

Lv, R. B., Wang, W. J., Li, X. (2020). Clinical observation on 63 suspected cases of new coronavirus pneumonia treated by lianhua qingwen granule combined with western medicine conventional therapy. *Journal of Traditional Chinese Medicine*. 61(8), 655-659. doi: 10.13288/j.11-2166/r.2020.08.003

Ma, L. (2015). Clinical observation on 156 cases of influenza treated by lianhua qingwen granule. *Healing Drugs*. 0(14), 235

Ma, S. Z., Kang, J. K. (2015). Clinical effect of lianhua qingwen keli combined with western medicine in treating 45 infantile herpangina. *Journal of Pediatrics of Traditional Chinese Medicine*. 11(5), 26-28. doi: CNKI:SUN:ZYEZ.0.2015-05-013. doi: CNKI:SUN:ZYEZ.0.2015-05-013

Ma, Y. P., Guo, Y. L., Kang, L., Zhao, L., Peng, J., Luo, G. Y. (2010). Analysis of curative effect of chinese medicine on influenza a (h1n1). *Shaanxi Journal of Traditional Chinese Medicine*. 31(10), 1351-1353. doi: 10.3969/j.issn.1000-7369.2010.10.055

Ma. Y. J. (2017). *Study on the efficacy and safety of jinqiaoreduqing granules in the treatment of common cold with wind—heat syndrome*. Master degree, Southern Medical University.

Meng, X.H., Li, L. Q., Wang, L. M. (2015). Effect analysis of lianhua qingwen granules on uremia complicated with pneumonia. *Contemporary Medical Symposium*. 13(17), 30-31. doi: CNKI:SUN:QYWA.0.2015-17-024

Meng, Y. (2014). *Clinical observation of lianhua qingwen capsule in the treatment of cold syndrome in the elderly*. Paper presented at the Basic and clinical research of collateral disease.

Mo, Q. Y., Liang, G. M., Huang, X. W. (2019). Clinical study on lianhua qingwen granules combined with vidarabine monophosphate in treatment of children with hand-foot-and-mouth disease. *Drugs & Clinic*. 34(8), 2402-2406. doi: CNKI:SUN:GWZW.0.2019-08-033

Ouyang, H. X., Tang, Q. Y., Chen, Y. Z., Wei, Y., Li, G. S. (2010). Clinical observation of lianhua qingwen department of emergency, capsules in treatment of the influenza a/h1n1. *China Medical Herald*. 7(30), 6-8. doi: 10.3969/j.issn.1673-7210.2010.30.003

Pang, H. Q. (2013). Clinical observation on 68 cases of acute viral pharyngitis treated by lianhua qingwen capsule. *Zhejiang Journal of Traditional Chinese Medicine*. 48(09), 668. doi: CNKI:SUN:ZJZZ.0.2013-09-036

Peng, X. H. (2013). *The rearch on clinical efficacy of facilitating the flow of lung-qi and clear lung-heart of community-acquired pneumonia (lung-heart syndrome)*. Master degree, Traditional Chinese Medicine University Of Guangzhou.

Qiao, H. L. (2018). Value of lianhua qingwen granule in the treatment of children with recurring respiratory infection. *China Rural Health*. 4(20), 47-48. doi: CNKI:SUN:NCWS.0.2018-20-047

Qin, X. Y. (2014). *Clinical observation on 72 cases of herpes zoster treated by lianhuaqingwen capsule combined with acyclovir*. Paper presented at the Basic and clinical research of collateral disease.

Qin, Y. (2017). Effect view of lianhua qingwen capsule on the treatment of influenza. *Diet Health*. 4(13), 62-63. 10.3969/j.issn.2095-8439.2017.13.070

Qiu, W. (2016). Observation on curative effects of lianhua qingwen capsule on acute suppurative tonsillitis. *Medical Journal of National Defending Forces in Southwest China*. 26(5), 536-539. doi: CNKI:SUN:XNGF.0.2016-05-027

Qu, H., Zhao, B. (2013). Application of lianhua qingwen capsules in fever clinic. *Chinese Journal of School Doctor*. 27(08), 613-614. doi: CNKI:SUN:XIYI.0.2013-08-027

Shang, L., Jia, S. S., Xu, L. Z. (2013a). Lianhua qingwen capsule combined with golden buckwheat tablets in the treatment of influenza a h1n1 influenza clinical research. *China Hwalth Care & nutrition*. 211-212(2).

Sheng, X., Shen, L.Y., Xu, Y. L. (2013b). Observation on the treatment of 186 cases of hand foot and mouth disease with lianhua qingwen granules. *Zhejiang Journal of Traditional Chinese Medicine*. 48(10), 778. doi: CNKI:SUN:ZJZZ.0.2013-10-060

Shi, J. L. (2014). *Clinical observation of liathuaqingwen capsule in the treatment of elderly patients with community-acquired pneumonia with fever*. Paper presented at the Basic and clinical research of collateral disease.

Shi, Q. Y. (2020). Observation on therapeutic effect of oseltamivir phosphate capsule combined with lianhua qingwen capsule on viral cold. *Journal of Practical Traditional Chinese Medicine*. 36(11), 1438-1439. doi: CNKI:SUN:ZYAO.0.2020-11-047

Shi, X. X., Chai, H. Y., Huang, Y. H, Zhang, Q. (2016). The effect of lianhuaqingwen granule on the immune function of mycoplasma pneumonitis in children. *Contemporaty Medicine*. 22(31), 5-6, 7. doi: 10.3969/j.issn.1009-4393.2016.31.003

Sun, D. D. (2019). Evaluation of the efficacy and safety of lianhuaqingwen granule combined with cefoperazone sodium-sulbactam sodium in patients with community-acquired pneumonia. *Anti-Infection Pharmacy*. 16(8), 1423-1425. doi: 10.13493/j.issn.1672-7878.2019.08-044

Sun, R. (2018). Clinical observation of lianhua qingwen capsule in the treatment of influenza. *Journal of North Pharmacy*. 15(7), 68-69. doi: CNKI:SUN:BFYX.0.2018-07-054

Sun, S. G. (2014). Analysis of 15 cases of severe influenza a (h1n1) treated with oseltamivir combined with lianhua qingwen capsule. *Journal of Guangxi Medical University*. 31(04), 692-693. doi: 10.16190/j.cnki.45-1211/r.2014.04.131

Sun, Y. Y, Li, M. X, Qin, N. (2020). A comparative study on the clinical efficacy of qingkailing and lianhuaqingwen capsules for relieving the symptoms of upper respiratory tract infection. *Contemporary Medicine*. 26(24), 95-97. doi: CNKI:SUN:DDYI.0.2020-24-039

Tan, X., Yang, H. (2012). Clinical efficacy and safety of lianhuaqingwen capsule in the treatment of acute upper respiratory tract infection. *China & Foreign Medical Treatment*. 44-45(9). doi: 10.16662/j.cnki.1674-0742.2012.16.008

Tang, C. Y., Zheng, Y. H., Zheng, W. (2019). Lianhua qingwen capsule in treating of adult varicella for 28 cases. *Chinese Medicine Modern Distance Education of China*. 17(16), 67-68, 76. doi: CNKI:SUN:ZZYY.0.2019-16-029

Tang, H. M, Chen, H. (2020). Study on the curative effect of lianhua qingwen capsule combined with oseltamivir phosphate capsule in the treatment of influenza. *Journal of Medical Aesthetice and Cosmetology*. 29(14), 19-20.

Tang, R. (2018). Clinical effect of lianhua qingwen capsule on acute upper respiratory tract infection. *Psychological Monthly*. 4(12), 90. doi: 10.19738/j.cnki.psy.2018.12.080

Wan, G. J., Sun, L. Y., Zhang, S. T. (2020). Clinical effect and adverse reaction of oseltamivir phosphate in the treatment of influenza. *Medical Diet and Health*. 18(2), 104-107. doi: CNKI:SUN:YXSL.0.2020-02-078

Wang, B. L., Gao, B. (2013). Lianhua qingwen decoction was used to treat 60 cases of aids exogenous fever. *Traditional Chinese Medicinal Research*. 26(12), 19-21. doi: CNKI:SUN:ZYYJ.0.2013-12-011

Wang, B. F. (2016). Clinical study on lianhua qingwen capsule in treating influenza ⅱ phase 1. *China Health Care & Nutrition*. 26(1), 157.

Wang, C. T. (2015). Analysis of curative effect of lianhua qingwen on upper respiratory tract infection. *Journal of Jiujiang University (Natural Science Edition)*. 30(01), 90-91. doi: 10.19717/j.cnki.jjun.2015.01.028

Wang, G. (2017). Clinical study on the treatment of hand, foot and mouth disease with lianhua qingwen granules. *Health for Everyone*. 4(10), 122. doi: CNKI:SUN:RRJK.0.2017-10-116

Wang, G.S., Zhao, L. X. (2015). Clinical observation of lianhuaqingwen capsule in elderly community acquired pneumonia with fever. *Oriental Diet Therapy and Health Care*. 5, 38.

Wang, H. L. (2018). Study on the efficacy and safety of lianhua qingwen granules in the treatment of respiratory tract infection. *Journal of North Pharmacy*. 15(05), 159. doi: CNKI:SUN:BFYX.0.2018-05-136

Wang, J. Y. (2019). Clinical observation of lianhua qingwen capsule in the treatment of influenza. *Medical Diet and Health*. 4(16), 29, 31. doi: CNKI:SUN:YXSL.0.2019-16-019

Wang, L. (2018). Brief analysis on the experience of lianhua qingwen capsule in treating influenza. *Healthmust-Readmagazine*(15), 259-260.

Wang, S. L., Hou, J. H, Zhang, Y., Guo, Y. R., Li, F. (2020). Clinical efficacy of modified weijingtang and maxing shigan tang combined with half-dose hormones on refractory mycoplcasma pneumonia in children caused by toxic heat closing lung. *Chinese Journal of Experimental Traditional Medical Formulae*. 26(10), 69-74. doi: 10.13422/j.cnki.syfjx.20201021

Wang, X. G, Cui, X. J., Liu. X. S., Liu, H. X., Li, Y. H. (2008a). Lianhua qingwen capsules for influenza: observation of clinical efficacy. *China Pharmacy.* (27), 2146-2148. doi: CNKI:SUN:ZGYA.0.2008-27-030

Wang, Y. B., Xie. Y. L, Yang, Y. M., Liu. L. M., Chen, Y. F. (2008c). Research of the therapeutie effect and sefety of lianhuaainewen cansule in treatment of respiratory tract infection. *Chinese Journal of Difficult and Complicated Cases.* (01), 24-26, doi: CNKI:SUN:YNBZ.0.2008-01-011

Wang, Y. B., Zhang, T. M., Yang, Y. M., Xie, Y. L., Liu, L. M., Li, Y. L. (2008b). Efficacy and safety of lianhua qingwen capsule in the treatment of viral cold. *Journal of Clinical Pulmonary Medicine.* (09), 1118-1119. doi: CNKI:SUN:LCFK.0.2008-09-010

Wang, Y. (2020). Study on the clinical effect of lianhua qingwen capsule on infantile influenza. *China Health Vision.* (16), 83.

Wang, Z., Li, X. (2011). Clinical analysis of 109 juvenile influenza a (h1n1) cases. *Chinese Community Doctors*. 13(19), 147. doi: CNKI:SUN:ZGSQ.0.2011-19-142

Wang, Z. Y., Huang, J. Q. (2021). Clinical observation of lianhua qingwen granule combined with ribavirin in the treatment of hand, foot and mouth disease in children. *Chinese Medical Journal of Metallurgical Industry*. 38(01), 25-26. doi: 10.13586/j.cnki.yjyx1984.2021.01.017.

Wei, H. (2016). Clinical study of lianhua qingwen capsule combined with terbutaline and budesonide in the treatment of acute exacerbation of chronic obstructive pulmonary disease. *Drugs & Clinic*. 31(07), 973-977. doi: CNKI:SUN:GWZW.0.2016-07-011

Wei, M., Song, Y. X., Zhong, H. (2014). Effect of lianhuaqingwen capsule on acute upper respiratory infection and their impact on the ifn-γ. *Chinese Journal of Difficult and Complicated Cases*. 13(04), 345-348. doi: CNKI:SUN:YNBZ.0.2014-04-006

Wei, Q., Luo, H. (2010). Clinical observation of lianhua qingwen capsule and oseltamivir in the treatment of mild influenza a (h1n1). *Guangming Journal of Chinese Medicine*. 25(12), 2318-2319. doi: 10.3969/j.issn.1672-1721.2011.17.030

Wei, Q. (2020). Clinical efficacy of lianhua qingwen granules in influenza. *Smart Healthcare*. 6(31), 178-179. doi: 10.19335/j.cnki.2096-1219.2020.31.072

Wen, J. B., Li, S. Q. (2019). Clinical effectiveness analysis of lianhua qingwen capsule in treating exogenous wind-heat syndrome of acute upper respiratory tract infection. *China Health Vision.* (9), 78-79. doi: 10.3969/j.issn.1005-0019.2019.09.126

Wen, Y. L., Yu, L. (2015). Effect of lianhua qingwen capsule on seasonal influenza with different traditional chinese medicine syndromes. *Diet Health.* (9), 3-4

Wu, C. L., Lou, H. J. (2015). Effect of lianhua qingwen capsule on chronic obstructive pulmonary disease in acute aggravation stage. *Chinese Journal of Rural Medicine and Pharmacy*. 22(22), 41, 46. doi: 10.19542/j.cnki.1006-5180.2015.22.023. doi: CNKI:SUN:GMZY.0.2006-11-048

Wu, J. H., Wang, Y. Y., Wei, M., Tian, Y., Wang, L. (2014). Effect analysis of lianhua qingwen capsule on acute upper respiratory tract infection. *Journal of Frontier of Medical.* (25), 162-163. doi: 10.3969/j.issn.2095-1752.2014.25.137. doi: 10.3969/j.issn.2095-1752.2014.25.137

Wu, Q. Y. (2014). Observation on the effect of lianhua qingwen capsule on epidemic upper respiratory tract infection. *Contemporary Medicine*. 20(14), 345-348. doi: CNKI:SUN:DDYI.0.2014-14-094. doi: CNKI:SUN:DDYI.0.2014-14-094

Wu, Q. Y., Chen, B. C., Chen, L. P., Ke, M. Y., Liu, X. J. (2006). Clinical observation of lianhua qingwen capsule on acute exacerbation of chronic pulmonary heart disease. *Guangming Journal of Chinese Medicine.* (11), 70-71. doi: CNKI:SUN:GMZY.0.2006-11-048

Xia, N., Han, F. (2019). Analysis of the application of lianhuaqingwen and oseltamivir phosphate capsules in the treatment of pneumonia. *China Health Care & Nutrition*. 29(16), 280.

Xie, Y. L. (2013). Lianhuaqingwen capsule was used to treat 30 cases of herpes simplex virus keratitis. *J. Tradit. Chin. Med.* 54(16), 1415-1416. doi: 10.13288/j.11-2166/r.2013.16.026

Xin, H. B. (2013). Clinical observation of lianhua qingwen capsule in treating epidemic upper respiratory tract infection. *Practical Journal of Cardiac Cerebral Pneumal and Vascular Disease*. 21(04), 70-71. doi: CNKI:SUN:SYXL.0.2013-04-033

Xu, G. X. (2013). A randomized parallel control study of huatan zhike recipe combined with lianhua qingwen capsule in the treatment of respiratory tract infection. *Journal of Practical Traditional Chinese Internal Medicine*. 27(11), 9-10. doi: CNKI:SUN:SYZY.0.2013-11-006

Xu, G. F., Gao, P., Liu, P., Zhao, Y., Lai, Y. W., Li, G. H., et al. (2020). Clinical efficacy of lianhua qingwen capsules combined with azithromycin in treatment of mycoplasma pneumoniae and its influence on immune function. *Drug Evaluation Research*. 43(11), 2241-2244. doi: CNKI:SUN:YWPJ.0.2020-11-015

Xu, X. R., Jin, Q., Xu, W., Zhou, Y. (2015). Clinical observation of lianhuaqingwen granule in adjuvant treatment of community acquired pneumonia with heat toxicity. *Zhejiang Clinical Medical Journal*. 17(10), 1729-1730.

Xu, X. X., Jiao, W., Wu, J. T. (2012). Clinical comparative study of lianhua qingwen capsule and acupuncture in the treatment of high fever and shen-fainting syndrome of zang fu organ closure in stroke. *Chinese Journal of Integrative Medicine on Cardio-Cerebrovascular Disease*. 10(10), 1198-1199. doi: CNKI:SUN:ZYYY.0.2012-10-028

Xun, H. J., Du, M. X.. (2011). Observation on the curative effect of lianhua qingwen capsule in treating viral cold. *Guide of China Medicine*. 9(19), 306-307. doi: 10.15912/j.cnki.gocm.2011.19.028

Yan, G. X., Mu, G. Y., Gao, Y. Z. (2014). Observation on the curative effect of lianhua qingwen capsule in treating cold. *Journal of Frontier of Medical.* (32), 202-203. doi: 10.3969/j.issn.2095-1752.2014.32.202

Yang, C. X. (2016a). Comparative analysis of qinzhiyu zhike prescription and lianhua qingwen granules in the treatment of cough phlegm heat depression lung syndrome. *Clinical Journal of Chinese Medicine*. 8(15), 15-16. doi: CNKI:SUN:ZYLY.0.2016-15-011

Yang, J. S. Wang, X. Y., Wang, J. C. (2011). Clinical analysis of 60 cases of acute upper respiratory tract infection treated by lianhua qingwen capsule. *Medical Information*. 24(9), 157

Yang, L. B., Ji, Z. H., Gao, X. D., Gu, C. H. (2005a). Phase ii clinical study of lianhua qingwen capsule in the treatment of influenza. *Traditional Chinese Drug Research and Clinical Pharmacology.* (04), 290-293. doi: 10.19378/j.issn.1003-9783.2005.04.023

Yang, L. B., Ji, Z. H., Wang, B. Q. (2005b). Clinical observation of the therapeutic effect of lianhuaqingwen capsule on 280 cases of influenza

. *Chinese Journal of Difficult and Complicated Cases.* (05), 276-278. doi: CNKI:SUN:YNBZ.0.2005-05-010

Yang, Q. (2012). *The syndrome type distribution of 115 cold cases in chengdu da ci sicommunity in 2012 spring and the clinical observation of " gan du qin" inthe treatment of acute viral upper respiratory infection (syndrome of heatinvading lung)*. Master degree, Chengdu University of Traditional Chinese Medicine.

Yang, Y. P. (2016b). Observation of therapeutic effect of lianhua qingwen granule on community-acquired pneumonia. *Chinese Journal of Clinical Rational Drug Use*. 9(6A), 71-72

Yang, Y. P., Ma, H. R. (2012). Analysis of the curative effect of lianhua qingwen capsule in treating influenza. *Guide of China Medicine*. 10(25), 606-607. doi: 10.15887/j.cnki.13-1389/r.2016.16.039

Yang, Z. X. (2011). Effect of lianhua qingwen capsule on acute upper respiratory tract infection. *Chinese Journal of Ethnomedicine and Ethnopharmacy*. 20(14), 91. doi: CNKI:SUN:MZMJ.0.2011-14-065

Yao, L. (2012). Guizhi decoction for treatment of cold and wind during pregnancy. *World Health Digest.* (35), 404-405. doi: 10.3969/j.issn.1672-5085.2012.35.439

Ye, C. J., Zhang, F., Zhu, Y. Y. (2013). Clinical study of lianhua qingwen capsule in treatment of influenza combined with bronchial pneumonia. *Chinese Journal of Experimental Traditional Medical Formulae*. 19(24), 329-331. doi: CNKI:SUN:ZSFX.0.2013-24-088

Yu, C., Wang, L., Li, X. C., Zhang, D., Zhang, J. (2017). Clinic al curative effect study of cold -fever -treating mixture on wind -heat type common cold. *Journal of Emergency in Traditional Chinese Medicine*. 26(01), 167-170. doi: CNKI:SUN:ZYJZ.0.2017-01-060

Yu, H. Y., Lin, C. Z., Jiang, H. D. (2011). The effect of traditional chinese and western medicine treatment in designated hospitals and community. *Journal of Qingdao University (Medical Sciences)*. 47(06), 517-519. doi: CNKI:SUN:BATE.0.2011-06-018

Yu, P., Li, Y. Z., Wan, S. B., Wang, Y. (2020). Efficacy of lianhua qingwen combined with arbidol in the treatment of mild covid-19 pneumonia. *Chinese Pharmaceutical Journal*, 1-9.

Yuan, Z. J. (2014). Efficacy and safety evaluation of lianhua qingwen capsule in the treatment of viral cold. *Chinese Journal of Clinical Rational Drug Use*. 7(12), 137-138. doi: 10.15887/j.cnki.13-1389/r.2014.12.012

Zeng, H. M., Lv, W. Q. (2020). Comparison of efficacy between lianhua qingwen granule and oseltamivir granule in the treatment of infantile influenza a. *Chinese Journal of Rural Medicine and Pharmacy*. 27(23), 14-15. doi: 10.19542/j.cnki.1006-5180.004620

Zhai, L. Y. (2014). Clinical effect of lianhuaqingwen capsule on acute upper respiratory tract infection. *Heilongjiang Medicine Journal*. 27(02), 377-378

Zhang, H. B. (2020a). Clinical efficacy and safety of oseltamivir phosphate in the treatment of influenza. *Healthmust-Readmagazine*(13), 127-128. doi: 10.14035/j.cnki.hljyy.2014.02.020

Zhang, J. P. (2018a). Observation of the effect of lianhuaqingwen granule combined with moxifloxacin tablets on elderly community-acquired pneumonia. *Chinese journal of health care nutrition*. 28(27), 235. doi: 10.3969/j.issn.1004-7484.2018.27.217

Zhang, J. L., Tan, Y. P. (2010a). Therapeutic effect of lianhua qingwen capsule on 30 cases of pulmonary infection. *World Chinese Medicine*. 5(03), 158-159. doi: CNKI:SUN:SJZA.0.2010-03-006

Zhang, J., Yu, Y. F., Hou, L. Y., Chen, S. H., Bai, X. Y., Dong, Y. X. et al. (2011b). Clinical observation of kangfuxin liquid combined with lianhuaqingwen capsule in treatment of hand foot and mouth disease. *Chinese Journal of Information on Traditional Chinese Medicine*. 18(01), 69-70. doi: CNKI:SUN:YNBZ.0.2010-07-031

Zhang, J., Yu, Y. F., Yang, C. X., Hou, L. Y. (2010). Effect of lianhuaqingwen preparation combined with kangfuxin on hand, foot and mouth disease. *Chinese Journal of Difficult and Complicated Cases*. 9(07), 524-525. doi: CNKI:SUN:XXYY.0.2011-01-036

Zhang, J., Qin, J. G. (2019). Observation on the curative effect of lianhua qingwen capsule and oseltamivir phosphate in the treatment of influenza. *Heilongjiang Medicine Journal*. 32(05), 1047-1049. doi: 10.14035/j.cnki.hljyy.2019.05.018

Zhang, J. X. (2020b). Clinical efficacy and safety of oseltamivir phosphate in the treatment of influenza. *Electronic Journal of Clinical Medical Literature*. 7(47), 143, 147. doi: 10.16281/j.cnki.jocml.2020.47.099

Zhang, L., Han, L. (2018b). Clinical observation of lianhuaqingwen capsules combined with piperacillin and sulbactam in treatment of pneumonia. *Drugs & Clinic*. 33(8), 1960-1963. doi: CNKI:SUN:GWZW.0.2018-08-022

Zhang, L. (2018c). Effect of lianhua qingwen capsule in treatment of influenza. *Doctor*. 15(05), 159. doi: 10.19604/j.cnki.dys.2018.z1.083

Zhang, L. S., Li, D. D. Gu, C. F. (2015). Observation on the curative effect of lianhua qingwen granule in the adjuvant treatment of non-severe community-acquired pneumonia. *Chinese Journal of Traditional Medical Science and Technology*. 22(06), 733. doi: CNKI:SUN:TJYY.0.2015-06-083

Zhang, L. Y. (2020c). Clinical effect analysis of lianhua qingwen granules combined with antiviral treatment of children herpetic pharyngitis. *Chinese Journal of School Doctor*. 34(04), 300-301, 311. doi: CNKI:SUN:XIYI.0.2020-04-025

Zhang, M. (2014). Study on the effect of lianhua qingwen capsule in treating influenza. *Biotech World*. 4(06), 61. doi: CNKI:SUN:SWJJ.0.2014-06-053

Zhang, Q. H., Li, C., Wu, J. H., Xiao, M. M., Sun, Y. (2012). Clinical observation of lianhuaqingwen capsule combined with jinqiaomai tablet in influenza a/h1n1. *Journal of Emergency in Traditional Chinese Medicine*. 21(03), 345-346. doi: 10.3969/j.issn.1004-745X.2012.03.001

Zhang, X. J., Li, J. (2016). Comparative analysis of qinzhiyu zhike prescription and lianhua qingwen granules in the treatment of cough phlegm heat depression lung syndrome. 8(15), 15-16. doi: CNKI:SUN:ZGCF.0.2016-05-065

Zhang, X. L. (2016). Efficacy and safety of lianhua qingwen capsule in the treatment of acute upper respiratory tract infection. *Cardiovascular Disease Electronic Journal of Integrated Traditional Chinese and Western Medicine*. 4(34), 68. doi: 10.16282/j.cnki.cn11-9336/r.2016.34.161

Zhang, Y. (2018). Antiviral application analysis of lianhua qingwen granules and oseltamivir phosphate granules. *China Health Care & Nutrition*. 28(19), 18. doi: 10.3969/j.issn.1004-7484.2018.19.018

Zhang, Y. (2019). Clinical observation of xiaochaihu granules combined with lianhua qingwen capsule in the treatment of acute upper respiratory tract infection. *China's Naturopathy*. 27(24), 34-36. doi: 10.19621/j.cnki.11-3555/r.2019.2419

Zhao, M. J., Zhao, X. Q., Zhao,W. (2015). Clinical effects of lianhua oingwen capsule in the treatment of acute respiratory infections. *Chinese Journal of Nosocomiology*. 25(04), 839-841. doi: 10.11816/cn.ni.2015-143006

Zheng, X. H., Huang, K. Q., Chen, Q. W., Zhang, R. B. (2015). Clinical observation of lianhua qingwen capsule in the treatment of pediatric epidemic influenza. *China Pharmacy*. 24(28), 2635-2637. doi: CNKI:SUN:ZGYA.0.2013-28-020. doi: CNKI:SUN:ZGYA.0.2013-28-020

Zheng, Y. (2010). Analysis of curative effect of lianhua qingwen capsule on 65 cases of influenza. *Chinese Community Doctors*. 12(07), 94. doi: CNKI:SUN:ZGSQ.0.2010-07-119

Zhou, B. (2011). Observation on the curative effect of lianhua qingwen capsule in treating influenza. *Chinese Journal of Misdiagnostics*. 18(01), 69-70. doi: CNKI:SUN:ZWZX.0.2011-28-029

Zhou, J. L. (2018). Clinical effect analysis of oseltamivir combined with lianhua qingwen capsule for treatment of influenza a (h1n1). *Electronic Journal of Clinical Medical Literature*. 5(22), 10-11. doi: 10.3877/j.issn.2095-8242.2018.22.007

Zhou, K. Q. (2017). To explore the curative effect of lianhua qingwen capsule on cold. *Psychological Doctor*. 23(12), 68-69.

Zhou, L. (2015). Clinical observation on the treatment of hand, foot and mouth disease by lianhua qingwen granule. *Journal of New Chinese Medicine*. 47(06), 203-204. doi: 10.13457/j.cnki.jncm.2015.06.097

Zhou, Y. H. (2019). Observation on the symptom outcome of lianhua qingwen capsule in the treatment of influenza. *Psychological Monthly*. 14(11), 206. doi: 10.19738/j.cnki.psy.2019.11.176

Zhu, J., Luo, H. Q., Sheng, M. F. (2015). Comparison of lianhua qingwen capsule combined with western medicine and simple western medicine in the treatment of pulmonary infection. *Liaoning Journal of Traditional Chinese Medicine*. 42(04), 797-799. doi: 10.13192/j.issn.1000-1719.2015.04.053

Zou, H. X. (2014). Clinical effect of lianhuaqingwen granules combined with moxifloxacin tablets in the treatment of acquired pneumonia in the elderly community. *Medical Aesthetics and Cosmetology.* (7), 203-204.

Zou, Y., Huang, T. H. (2015). Clinical observation of lianhua qingwen capsule in the treatment of influenza. *Journal of Modern Medicine & Health*. 31(04), 590-592. doi: CNKI:SUN:XYWS.0.2015-04-049

Zuo, J. L., Xu, Y. H. (2006). Clinical study of lianhua qingwen capsule in treating acute upper respiratory tract infection. *Journal of Public Health and Preventive Medicine.* (06), 78-79. doi: CNKI:SUN:FBYF.0.2006-06-04
