# Supplementary material for: The Adverse Reactions of Lianhua Qingwen Capsule/Granule Compared With Conventional Drug in Clinical Application: A Meta-Analysis
Source: Front Pharmacol. 2022 Jan 27;13:764774. doi: 10.3389/fphar.2022.764774 (PMC8830515; doi:10.3389/fphar.2022.764774)
Supplement: Supplementary file 2 [file Table2.DOCX]

Supplementary Table 2. Summary table of all of the papers cited.

| **Study** | **Formulation** | **Source** | **Species, concentration** | **Quality control reported? (Y/N) *** | **Chemical analysis reported? (Y/N)** |
| --- | --- | --- | --- | --- | --- |
| Liu et al. (2021) | Lianhua Qingwen capsule | Yiling Pharmaceutical CO., LTD | Lianhua Qingwen, 0.35g | Y, Z20040063 | N |
| Wang et al. (2021) | Lianhua Qingwen granule | Yiling Pharmaceutical CO., LTD | Lianhua Qingwen, 6g | Y, Z20100040 | N |
| Hu et al. (2021) | Lianhua Qingwen capsule | Yiling Pharmaceutical CO., LTD | Lianhua Qingwen, 0.35g | Y, Z20040063 | N |
| Lin et al. (2020) | Lianhua Qingwen granule | Yiling Pharmaceutical CO., LTD | Lianhua Qingwen, 6g | Y, Z20100040 | N |
| Cai et al. (2020) | Lianhua Qingwen granule | Yiling Pharmaceutical CO., LTD | Lianhua Qingwen, 6g | Y, Z20100040 | N |
| Zeng et al. (2020) | Lianhua Qingwen granule | Yiling Pharmaceutical CO., LTD | Lianhua Qingwen, 6g | Y, Z20100040 | N |
| Chao et al. (2020) | Lianhua Qingwen capsule | Yiling Pharmaceutical CO., LTD | Lianhua Qingwen, 0.35g | Y, Z20040063 | N |
| Chen et al. (2020a) | Lianhua Qingwen granule | Yiling Pharmaceutical CO., LTD | Lianhua Qingwen, 6g | Y, Z20100040 | N |
| Chen et al. (2020b) | Lianhua Qingwen granule | Yiling Pharmaceutical CO., LTD | Lianhua Qingwen, 6g | Y, Z20100040 | N |
| Fang et al. (2020) | Lianhua Qingwen capsule | Yiling Pharmaceutical CO., LTD | Lianhua Qingwen, 0.35g | Y, Z20040063 | N |
| Fu (2020) | Lianhua Qingwen capsule | Yiling Pharmaceutical CO., LTD | Lianhua Qingwen, 0.35g | Y, Z20040063 | N |
| Guo et al. (2020) | Lianhua Qingwen granule | Yiling Pharmaceutical CO., LTD | Lianhua Qingwen, 6g | Y, Z20100040 | N |
| Jian (2020) | Lianhua Qingwen granule | Yiling Pharmaceutical CO., LTD | Lianhua Qingwen, 6g | Y, Z20100040 | N |
| Jiang (2020) | Lianhua Qingwen capsule | Yiling Pharmaceutical CO., LTD | Lianhua Qingwen, 0.35g | Y, Z20040063 | N |
| Lei (2020) | Lianhua Qingwen granule | Yiling Pharmaceutical CO., LTD | Lianhua Qingwen, 6g | Y, Z20100040 | N |
| Li et al. (2020) | Lianhua Qingwen granule | Yiling Pharmaceutical CO., LTD | Lianhua Qingwen, 6g | Y, Z20100040 | N |
| Li (2020a) | Lianhua Qingwen capsule | Yiling Pharmaceutical CO., LTD | Lianhua Qingwen, 0.35g | Y, Z20040063 | N |
| Li (2020b) | Lianhua Qingwen granule | Yiling Pharmaceutical CO., LTD | Lianhua Qingwen, 6g | Y, Z20100040 | N |
| Liu (2020a) | Lianhua Qingwen capsule | Yiling Pharmaceutical CO., LTD | Lianhua Qingwen, 0.35g | Y, Z20040063 | N |
| Liu (2020b) | Lianhua Qingwen granule | Yiling Pharmaceutical CO., LTD | Lianhua Qingwen, 6g | Y, Z20100040 | N |
| Liu (2020c) | Lianhua Qingwen capsule | Yiling Pharmaceutical CO., LTD | Lianhua Qingwen, 0.35g | Y, Z20040063 | N |
| Liu et al. (2020) | Lianhua Qingwen granule | Yiling Pharmaceutical CO., LTD | Lianhua Qingwen, 6g | Y, Z20100040 | N |
| Lu et al. (2020) | Lianhua Qingwen capsule | Yiling Pharmaceutical CO., LTD | Lianhua Qingwen, 0.35g | Y, Z20040063 | N |

Supplementary Table 2 continued.

| **Study** | **Formulation** | **Source** | **Species, concentration** | **Quality control reported? (Y/N) *** | **Chemical analysis reported? (Y/N)** |
| --- | --- | --- | --- | --- | --- |
| Lv et al. (2020) | Lianhua Qingwen granule | Yiling Pharmaceutical CO., LTD | Lianhua Qingwen, 6g | Y, Z20100040 | N |
| Shi (2020) | Lianhua Qingwen capsule | Yiling Pharmaceutical CO., LTD | Lianhua Qingwen, 0.35g | Y, Z20040063 | N |
| Sun, et al. (2020) | Lianhua Qingwen capsule | Yiling Pharmaceutical CO., LTD | Lianhua Qingwen, 0.35g | Y, Z20040063 | N |
| Tang et al. (2020) | Lianhua Qingwen capsule | Yiling Pharmaceutical CO., LTD | Lianhua Qingwen, 0.35g | Y, Z20040063 | N |
| Wan et al. (2020) | Lianhua Qingwen capsule | Yiling Pharmaceutical CO., LTD | Lianhua Qingwen, 0.35g | Y, Z20040063 | N |
| Wang et al. (2020) | Lianhua Qingwen granule | Yiling Pharmaceutical CO., LTD | Lianhua Qingwen, 6g | Y, Z20100040 | N |
| Wang (2020) | Lianhua Qingwen capsule | Yiling Pharmaceutical CO., LTD | Lianhua Qingwen, 0.35g | Y, Z20040063 | N |
| Wei, (2020) | Lianhua Qingwen granule | Yiling Pharmaceutical CO., LTD | Lianhua Qingwen, 6g | Y, Z20100040 | N |
| Xu et al. (2020) | Lianhua Qingwen capsule | Yiling Pharmaceutical CO., LTD | Lianhua Qingwen, 0.35g | Y, Z20040063 | N |
| Yu et al. (2020) | Lianhua Qingwen granule | Yiling Pharmaceutical CO., LTD | Lianhua Qingwen, 6g | Y, Z20100040 | N |
| Zhang (2020a) | Lianhua Qingwen granule | Yiling Pharmaceutical CO., LTD | Lianhua Qingwen, 6g | Y, Z20100040 | N |
| Zhang (2020b) | Lianhua Qingwen capsule | Yiling Pharmaceutical CO., LTD | Lianhua Qingwen, 0.35g | Y, Z20040063 | N |
| Zhang (2020c) | Lianhua Qingwen granule | Yiling Pharmaceutical CO., LTD | Lianhua Qingwen, 6g | Y, Z20100040 | N |
| Du et al. (2019) | Lianhua Qingwen capsule | Yiling Pharmaceutical CO., LTD | Lianhua Qingwen, 0.35g | Y, Z20040063 | N |
| Feng et al. (2019) | Lianhua Qingwen capsule | Yiling Pharmaceutical CO., LTD | Lianhua Qingwen, 0.35g | Y, Z20040063 | N |
| Han (2019) | Lianhua Qingwen capsule | Yiling Pharmaceutical CO., LTD | Lianhua Qingwen, 0.35g | Y, Z20040063 | N |
| Hao (2019) | Lianhua Qingwen capsule | Yiling Pharmaceutical CO., LTD | Lianhua Qingwen, 0.35g | Y, Z20040063 | N |
| Hua et al. (2019) | Lianhua Qingwen granule | Yiling Pharmaceutical CO., LTD | Lianhua Qingwen, 6g | Y, Z20100040 | N |
| Ji (2019) | Lianhua Qingwen capsule | Yiling Pharmaceutical CO., LTD | Lianhua Qingwen, 0.35g | Y, Z20040063 | N |
| Li (2019) | Lianhua Qingwen capsule | Yiling Pharmaceutical CO., LTD | Lianhua Qingwen, 0.35g | Y, Z20040063 | N |
| Liang (2019) | Lianhua Qingwen granule | Yiling Pharmaceutical CO., LTD | Lianhua Qingwen, 6g | Y, Z20100040 | N |
| Liang et al. (2019) | Lianhua Qingwen capsule | Yiling Pharmaceutical CO., LTD | Lianhua Qingwen, 0.35g | Y, Z20040063 | N |
| Mo et al. (2019) | Lianhua Qingwen granule | Yiling Pharmaceutical CO., LTD | Lianhua Qingwen, 6g | Y, Z20100040 | N |

Supplementary Table 2 continued.

| **Study** | **Formulation** | **Source** | **Species, concentration** | **Quality control reported? (Y/N) *** | **Chemical analysis reported? (Y/N)** |
| --- | --- | --- | --- | --- | --- |
| Sun (2019) | Lianhua Qingwen granule | Yiling Pharmaceutical CO., LTD | Lianhua Qingwen, 6g | Y, Z20100040 | N |
| Tang et al. (2019) | Lianhua Qingwen capsule | Yiling Pharmaceutical CO., LTD | Lianhua Qingwen, 0.35g | Y, Z20040063 | N |
| Wang (2019) | Lianhua Qingwen capsule | Yiling Pharmaceutical CO., LTD | Lianhua Qingwen, 0.35g | Y, Z20040063 | N |
| Wen et al. (2019) | Lianhua Qingwen capsule | Yiling Pharmaceutical CO., LTD | Lianhua Qingwen, 0.35g | Y, Z20040063 | N |
| Xia et al. (2019) | Lianhua Qingwen capsule | Yiling Pharmaceutical CO., LTD | Lianhua Qingwen, 0.35g | Y, Z20040063 | N |
| Zhang et al. (2019) | Lianhua Qingwen capsule | Yiling Pharmaceutical CO., LTD | Lianhua Qingwen, 0.35g | Y, Z20040063 | N |
| Zhang (2019) | Lianhua Qingwen capsule | Yiling Pharmaceutical CO., LTD | Lianhua Qingwen, 0.35g | Y, Z20040063 | N |
| Zhou (2019) | Lianhua Qingwen capsule | Yiling Pharmaceutical CO., LTD | Lianhua Qingwen, 0.35g | Y, Z20040063 | N |
| Bai et al. (2019) | Lianhua Qingwen capsule | Yiling Pharmaceutical CO., LTD | Lianhua Qingwen, 0.35g | Y, Z20040063 | N |
| Cheng (2018) | Lianhua Qingwen capsule | Yiling Pharmaceutical CO., LTD | Lianhua Qingwen, 0.35g | Y, Z20040063 | N |
| Hu (2018) | Lianhua Qingwen granule | Yiling Pharmaceutical CO., LTD | Lianhua Qingwen, 6g | Y, Z20100040 | N |
| Hu et al. (2018) | Lianhua Qingwen capsule | Yiling Pharmaceutical CO., LTD | Lianhua Qingwen, 0.35g | Y, Z20040063 | N |
| Huang (2018) | Lianhua Qingwen capsule | Yiling Pharmaceutical CO., LTD | Lianhua Qingwen, 0.35g | Y, Z20040063 | N |
| Kong (2018) | Lianhua Qingwen capsule | Yiling Pharmaceutical CO., LTD | Lianhua Qingwen, 0.35g | Y, Z20040063 | N |
| Li (2018) | Lianhua Qingwen capsule | Yiling Pharmaceutical CO., LTD | Lianhua Qingwen, 0.35g | Y, Z20040063 | N |
| Li et al. (2018a) | Lianhua Qingwen granule | Yiling Pharmaceutical CO., LTD | Lianhua Qingwen, 6g | Y, Z20100040 | N |
| Li et al. (2018b) | Lianhua Qingwen granule | Yiling Pharmaceutical CO., LTD | Lianhua Qingwen, 6g | Y, Z20100040 | N |
| Liu (2018) | Lianhua Qingwen granule | Yiling Pharmaceutical CO., LTD | Lianhua Qingwen, 6g | Y, Z20100040 | N |
| Lv et al. (2018) | Lianhua Qingwen capsule | Yiling Pharmaceutical CO., LTD | Lianhua Qingwen, 0.35g | Y, Z20040063 | N |
| Qiao (2018) | Lianhua Qingwen granule | Yiling Pharmaceutical CO., LTD | Lianhua Qingwen, 6g | Y, Z20100040 | N |
| Wang (2018) | Lianhua Qingwen capsule | Yiling Pharmaceutical CO., LTD | Lianhua Qingwen, 0.35g | Y, Z20040063 | N |
| Sun (2018) | Lianhua Qingwen capsule | Yiling Pharmaceutical CO., LTD | Lianhua Qingwen, 0.35g | Y, Z20040063 | N |
| Tang (2018) | Lianhua Qingwen capsule | Yiling Pharmaceutical CO., LTD | Lianhua Qingwen, 0.35g | Y, Z20040063 | N |

Supplementary Table 2 continued.

| **Study** | **Formulation** | **Source** | **Species, concentration** | **Quality control reported? (Y/N) *** | **Chemical analysis reported? (Y/N)** |
| --- | --- | --- | --- | --- | --- |
| Wang (2018) | Lianhua Qingwen granule | Yiling Pharmaceutical CO., LTD | Lianhua Qingwen, 6g | Y, Z20100040 | N |
| Zhang (2018a) | Lianhua Qingwen granule | Yiling Pharmaceutical CO., LTD | Lianhua Qingwen, 6g | Y, Z20100040 | N |
| Zhang (2018b) | Lianhua Qingwen granule | Yiling Pharmaceutical CO., LTD | Lianhua Qingwen, 6g | Y, Z20100040 | N |
| Zhang (2018c) | Lianhua Qingwen capsule | Yiling Pharmaceutical CO., LTD | Lianhua Qingwen, 0.35g | Y, Z20040063 | N |
| Zhang (2018d) | Lianhua Qingwen granule | Yiling Pharmaceutical CO., LTD | Lianhua Qingwen, 6g | Y, Z20100040 | N |
| Zhou (2018) | Lianhua Qingwen capsule | Yiling Pharmaceutical CO., LTD | Lianhua Qingwen, 0.35g | Y, Z20040063 | N |
| Chen (2017) | Lianhua Qingwen capsule | Yiling Pharmaceutical CO., LTD | Lianhua Qingwen, 0.35g | Y, Z20040063 | N |
| Huang (2017) | Lianhua Qingwen capsule | Yiling Pharmaceutical CO., LTD | Lianhua Qingwen, 0.35g | Y, Z20040063 | N |
| Huang et al. (2017a) | Lianhua Qingwen granule | Yiling Pharmaceutical CO., LTD | Lianhua Qingwen, 6g | Y, Z20100040 | N |
| Huang et al. (2017b) | Lianhua Qingwen granule | Yiling Pharmaceutical CO., LTD | Lianhua Qingwen, 6g | Y, Z20100040 | N |
| Liao (2017) | Lianhua Qingwen capsule | Yiling Pharmaceutical CO., LTD | Lianhua Qingwen, 0.35g | Y, Z20040063 | N |
| Liu et al. (2017) | Lianhua Qingwen capsule | Yiling Pharmaceutical CO., LTD | Lianhua Qingwen, 0.35g | Y, Z20040063 | N |
| Ma (2017) | Lianhua Qingwen capsule | Yiling Pharmaceutical CO., LTD | Lianhua Qingwen, 0.35g | Y, Z20040063 | N |
| Qin (2017) | Lianhua Qingwen capsule | Yiling Pharmaceutical CO., LTD | Lianhua Qingwen, 0.35g | Y, Z20040063 | N |
| Wang (2017) | Lianhua Qingwen granule | Yiling Pharmaceutical CO., LTD | Lianhua Qingwen, 6g | Y, Z20100040 | N |
| Yu et al. (2017) | Lianhua Qingwen granule | Yiling Pharmaceutical CO., LTD | Lianhua Qingwen, 6g | Y, Z20100040 | N |
| Zhou (2017) | Lianhua Qingwen capsule | Yiling Pharmaceutical CO., LTD | Lianhua Qingwen, 0.35g | Y, Z20040063 | N |
| Chen (2016) | Lianhua Qingwen capsule | Yiling Pharmaceutical CO., LTD | Lianhua Qingwen, 0.35g | Y, Z20040063 | N |
| Fang et al. (2016) | Lianhua Qingwen capsule | Yiling Pharmaceutical CO., LTD | Lianhua Qingwen, 0.35g | Y, Z20040063 | N |
| Feng et al. (2016) | Lianhua Qingwen granule | Yiling Pharmaceutical CO., LTD | Lianhua Qingwen, 6g | Y, Z20100040 | N |
| Han (2016) | Lianhua Qingwen granule | Yiling Pharmaceutical CO., LTD | Lianhua Qingwen, 6g | Y, Z20100040 | N |
| Jin (2016) | Lianhua Qingwen capsule | Yiling Pharmaceutical CO., LTD | Lianhua Qingwen, 0.35g | Y, Z20040063 | N |
| Li (2016) | Lianhua Qingwen capsule | Yiling Pharmaceutical CO., LTD | Lianhua Qingwen, 0.35g | Y, Z20040063 | N |

Supplementary Table 2 continued.

| **Study** | **Formulation** | **Source** | **Species, concentration** | **Quality control reported? (Y/N) *** | **Chemical analysis reported? (Y/N)** |
| --- | --- | --- | --- | --- | --- |
| Li et al. (2016) | Lianhua Qingwen capsule | Yiling Pharmaceutical CO., LTD | Lianhua Qingwen, 0.35g | Y, Z20040063 | N |
| Qiu (2016) | Lianhua Qingwen capsule | Yiling Pharmaceutical CO., LTD | Lianhua Qingwen, 0.35g | Y, Z20040063 | N |
| Shi et al. (2016) | Lianhua Qingwen granule | Yiling Pharmaceutical CO., LTD | Lianhua Qingwen, 6g | Y, Z20100040 | N |
| Wang (2016) | Lianhua Qingwen capsule | Yiling Pharmaceutical CO., LTD | Lianhua Qingwen, 0.35g | Y, Z20040063 | N |
| Wei (2016) | Lianhua Qingwen capsule | Yiling Pharmaceutical CO., LTD | Lianhua Qingwen, 0.35g | Y, Z20040063 | N |
| Yang (2016a) | Lianhua Qingwen granule | Yiling Pharmaceutical CO., LTD | Lianhua Qingwen, 6g | Y, Z20100040 | N |
| Yang (2016b) | Lianhua Qingwen granule | Yiling Pharmaceutical CO., LTD | Lianhua Qingwen, 6g | Y, Z20100040 | N |
| Zhang et al. (2016) | Lianhua Qingwen capsule | Yiling Pharmaceutical CO., LTD | Lianhua Qingwen, 0.35g | Y, Z20040063 | N |
| Zhang (2016) | Lianhua Qingwen capsule | Yiling Pharmaceutical CO., LTD | Lianhua Qingwen, 0.35g | Y, Z20040063 | N |
| Bi et al. (2015) | Lianhua Qingwen capsule | Yiling Pharmaceutical CO., LTD | Lianhua Qingwen, 0.35g | Y, Z20040063 | N |
| Dong (2015) | Lianhua Qingwen capsule | Yiling Pharmaceutical CO., LTD | Lianhua Qingwen, 0.35g | Y, Z20040063 | N |
| Du et al. (2015) | Lianhua Qingwen granule | Yiling Pharmaceutical CO., LTD | Lianhua Qingwen, 6g | Y, Z20100040 | N |
| Fang (2015) | Lianhua Qingwen capsule | Yiling Pharmaceutical CO., LTD | Lianhua Qingwen, 0.35g | Y, Z20040063 | N |
| Feng (2015) | Lianhua Qingwen capsule | Yiling Pharmaceutical CO., LTD | Lianhua Qingwen, 0.35g | Y, Z20040063 | N |
| Guo (2015) | Lianhua Qingwen capsule | Yiling Pharmaceutical CO., LTD | Lianhua Qingwen, 0.35g | Y, Z20040063 | N |
| He (2015) | Lianhua Qingwen capsule | Yiling Pharmaceutical CO., LTD | Lianhua Qingwen, 0.35g | Y, Z20040063 | N |
| Li et al. (2015) | Lianhua Qingwen capsule | Yiling Pharmaceutical CO., LTD | Lianhua Qingwen, 0.35g | Y, Z20040063 | N |
| Li (2015a) | Lianhua Qingwen granule | Yiling Pharmaceutical CO., LTD | Lianhua Qingwen, 6g | Y, Z20100040 | N |
| Li (2015b) | Lianhua Qingwen capsule | Yiling Pharmaceutical CO., LTD | Lianhua Qingwen, 0.35g | Y, Z20040063 | N |
| Lin et al. (2015) | Lianhua Qingwen granule | Yiling Pharmaceutical CO., LTD | Lianhua Qingwen, 6g | Y, Z20100040 | N |
| Liu et al. (2015) | Lianhua Qingwen granule | Yiling Pharmaceutical CO., LTD | Lianhua Qingwen, 6g | Y, Z20100040 | N |
| Liu (2015) | Lianhua Qingwen capsule | Yiling Pharmaceutical CO., LTD | Lianhua Qingwen, 0.35g | Y, Z20040063 | N |
| Lu et al. (2015) | Lianhua Qingwen granule | Yiling Pharmaceutical CO., LTD | Lianhua Qingwen, 6g | Y, Z20100040 | N |

Supplementary Table 2 continued.

| **Study** | **Formulation** | **Source** | **Species, concentration** | **Quality control reported? (Y/N) *** | **Chemical analysis reported? (Y/N)** |
| --- | --- | --- | --- | --- | --- |
| Ma (2015) | Lianhua Qingwen granule | Yiling Pharmaceutical CO., LTD | Lianhua Qingwen, 6g | Y, Z20100040 | N |
| Ma et al. (2015) | Lianhua Qingwen granule | Yiling Pharmaceutical CO., LTD | Lianhua Qingwen, 6g | Y, Z20100040 | N |
| Meng et al. (2015) | Lianhua Qingwen granule | Yiling Pharmaceutical CO., LTD | Lianhua Qingwen, 6g | Y, Z20100040 | N |
| Wang (2015) | Lianhua Qingwen capsule | Yiling Pharmaceutical CO., LTD | Lianhua Qingwen, 0.35g | Y, Z20040063 | N |
| Wang et al. (2015) | Lianhua Qingwen capsule | Yiling Pharmaceutical CO., LTD | Lianhua Qingwen, 0.35g | Y, Z20040063 | N |
| Wen et al. (2015) | Lianhua Qingwen capsule | Yiling Pharmaceutical CO., LTD | Lianhua Qingwen, 0.35g | Y, Z20040063 | N |
| Wu et al. (2015) | Lianhua Qingwen capsule | Yiling Pharmaceutical CO., LTD | Lianhua Qingwen, 0.35g | Y, Z20040063 | N |
| Xu et al. (2015) | Lianhua Qingwen granule | Yiling Pharmaceutical CO., LTD | Lianhua Qingwen, 6g | Y, Z20100040 | N |
| Zhang et al. (2015) | Lianhua Qingwen granule | Yiling Pharmaceutical CO., LTD | Lianhua Qingwen, 6g | Y, Z20100040 | N |
| Zhao et al. (2015) | Lianhua Qingwen capsule | Yiling Pharmaceutical CO., LTD | Lianhua Qingwen, 0.35g | Y, Z20040063 | N |
| Zheng et al. (2015) | Lianhua Qingwen capsule | Yiling Pharmaceutical CO., LTD | Lianhua Qingwen, 0.35g | Y, Z20040063 | N |
| Zhou (2015) | Lianhua Qingwen granule | Yiling Pharmaceutical CO., LTD | Lianhua Qingwen, 6g | Y, Z20100040 | N |
| Zhu et al. (2015) | Lianhua Qingwen capsule | Yiling Pharmaceutical CO., LTD | Lianhua Qingwen, 0.35g | Y, Z20040063 | N |
| Zhou et al. (2015) | Lianhua Qingwen capsule | Yiling Pharmaceutical CO., LTD | Lianhua Qingwen, 0.35g | Y, Z20040063 | N |
| Chen (2014a) | Lianhua Qingwen capsule | Yiling Pharmaceutical CO., LTD | Lianhua Qingwen, 0.35g | Y, Z20040063 | N |
| Chen (2014b) | Lianhua Qingwen capsule | Yiling Pharmaceutical CO., LTD | Lianhua Qingwen, 0.35g | Y, Z20040063 | N |
| Cheng (2014) | Lianhua Qingwen capsule | Yiling Pharmaceutical CO., LTD | Lianhua Qingwen, 0.35g | Y, Z20040063 | N |
| Dai (2014) | Lianhua Qingwen capsule | Yiling Pharmaceutical CO., LTD | Lianhua Qingwen, 0.35g | Y, Z20040063 | N |
| Dai et al. (2014) | Lianhua Qingwen capsule | Yiling Pharmaceutical CO., LTD | Lianhua Qingwen, 0.35g | Y, Z20040063 | N |
| Ding et al. (2014) | Lianhua Qingwen capsule | Yiling Pharmaceutical CO., LTD | Lianhua Qingwen, 0.35g | Y, Z20040063 | N |
| Dong (2014) | Lianhua Qingwen granule | Yiling Pharmaceutical CO., LTD | Lianhua Qingwen, 6g | Y, Z20100040 | N |
| Dong et al. (2014) | Lianhua Qingwen capsule | Yiling Pharmaceutical CO., LTD | Lianhua Qingwen, 0.35g | Y, Z20040063 | N |
| Gao (2014) | Lianhua Qingwen granule | Yiling Pharmaceutical CO., LTD | Lianhua Qingwen, 6g | Y, Z20100040 | N |

Supplementary Table 2 continued.

| **Study** | **Formulation** | **Source** | **Species, concentration** | **Quality control reported? (Y/N) *** | **Chemical analysis reported? (Y/N)** |
| --- | --- | --- | --- | --- | --- |
| He (2014) | Lianhua Qingwen capsule | Yiling Pharmaceutical CO., LTD | Lianhua Qingwen, 0.35g | Y, Z20040063 | N |
| Jiang et al. (2014a) | Lianhua Qingwen capsule | Yiling Pharmaceutical CO., LTD | Lianhua Qingwen, 0.35g | Y, Z20040063 | N |
| Jiang et al. (2014b) | Lianhua Qingwen capsule | Yiling Pharmaceutical CO., LTD | Lianhua Qingwen, 0.35g | Y, Z20040063 | N |
| Li et al. (2014a) | Lianhua Qingwen granule | Yiling Pharmaceutical CO., LTD | Lianhua Qingwen, 6g | Y, Z20100040 | N |
| Li et al. (2014b) | Lianhua Qingwen capsule | Yiling Pharmaceutical CO., LTD | Lianhua Qingwen, 0.35g | Y, Z20040063 | N |
| Li (2014) | Lianhua Qingwen capsule | Yiling Pharmaceutical CO., LTD | Lianhua Qingwen, 0.35g | Y, Z20040063 | N |
| Mei (2014) | Lianhua Qingwen granule | Yiling Pharmaceutical CO., LTD | Lianhua Qingwen, 6g | Y, Z20100040 | N |
| Meng (2014) | Lianhua Qingwen capsule | Yiling Pharmaceutical CO., LTD | Lianhua Qingwen, 0.35g | Y, Z20040063 | N |
| Qin (2014) | Lianhua Qingwen capsule | Yiling Pharmaceutical CO., LTD | Lianhua Qingwen, 0.35g | Y, Z20040063 | N |
| Shi (2014) | Lianhua Qingwen capsule | Yiling Pharmaceutical CO., LTD | Lianhua Qingwen, 0.35g | Y, Z20040063 | N |
| Sun (2014) | Lianhua Qingwen capsule | Yiling Pharmaceutical CO., LTD | Lianhua Qingwen, 0.35g | Y, Z20040063 | N |
| Wei et al. (2014) | Lianhua Qingwen capsule | Yiling Pharmaceutical CO., LTD | Lianhua Qingwen, 0.35g | Y, Z20040063 | N |
| Wu et al. (2014) | Lianhua Qingwen capsule | Yiling Pharmaceutical CO., LTD | Lianhua Qingwen, 0.35g | Y, Z20040063 | N |
| Wu (2014) | Lianhua Qingwen capsule | Yiling Pharmaceutical CO., LTD | Lianhua Qingwen, 0.35g | Y, Z20040063 | N |
| Yan et al. (2014) | Lianhua Qingwen capsule | Yiling Pharmaceutical CO., LTD | Lianhua Qingwen, 0.35g | Y, Z20040063 | N |
| Yuan (2014) | Lianhua Qingwen capsule | Yiling Pharmaceutical CO., LTD | Lianhua Qingwen, 0.35g | Y, Z20040063 | N |
| Zhai (2014) | Lianhua Qingwen capsule | Yiling Pharmaceutical CO., LTD | Lianhua Qingwen, 0.35g | Y, Z20040063 | N |
| Zhang (2014) | Lianhua Qingwen capsule | Yiling Pharmaceutical CO., LTD | Lianhua Qingwen, 0.35g | Y, Z20040063 | N |
| Zhou (2014) | Lianhua Qingwen granule | Yiling Pharmaceutical CO., LTD | Lianhua Qingwen, 6g | Y, Z20100040 | N |
| Chen et al. (2013a) | Lianhua Qingwen granule | Yiling Pharmaceutical CO., LTD | Lianhua Qingwen, 6g | Y, Z20100040 | N |
| Chen et al. (2013a) | Lianhua Qingwen capsule | Yiling Pharmaceutical CO., LTD | Lianhua Qingwen, 0.35g | Y, Z20040063 | N |
| Chen (2013) | Lianhua Qingwen capsule | Yiling Pharmaceutical CO., LTD | Lianhua Qingwen, 0.35g | Y, Z20040063 | N |
| Gao et al. (2013) | Lianhua Qingwen capsule | Yiling Pharmaceutical CO., LTD | Lianhua Qingwen, 0.35g | Y, Z20040063 | N |

Supplementary Table 2 continued.

| **Study** | **Formulation** | **Source** | **Species, concentration** | **Quality control reported? (Y/N) *** | **Chemical analysis reported? (Y/N)** |
| --- | --- | --- | --- | --- | --- |
| Gong (2013) | Lianhua Qingwen capsule | Yiling Pharmaceutical CO., LTD | Lianhua Qingwen, 0.35g | Y, Z20040063 | N |
| He et al. | Lianhua Qingwen capsule | Yiling Pharmaceutical CO., LTD | Lianhua Qingwen, 0.35g | Y, Z20040063 | N |
| Jiang (2013) | Lianhua Qingwen capsule | Yiling Pharmaceutical CO., LTD | Lianhua Qingwen, 0.35g | Y, Z20040063 | N |
| Ju et al. (2013) | Lianhua Qingwen capsule | Yiling Pharmaceutical CO., LTD | Lianhua Qingwen, 0.35g | Y, Z20040063 | N |
| Pang (2013) | Lianhua Qingwen capsule | Yiling Pharmaceutical CO., LTD | Lianhua Qingwen, 0.35g | Y, Z20040063 | N |
| Peng (2013) | Lianhua Qingwen capsule | Yiling Pharmaceutical CO., LTD | Lianhua Qingwen, 0.35g | Y, Z20040063 | N |
| Qu et al. (2013) | Lianhua Qingwen capsule | Yiling Pharmaceutical CO., LTD | Lianhua Qingwen, 0.35g | Y, Z20040063 | N |
| Shang et al. (2013a) | Lianhua Qingwen capsule | Yiling Pharmaceutical CO., LTD | Lianhua Qingwen, 0.35g | Y, Z20040063 | N |
| Sheng et al. (2013b) | Lianhua Qingwen granule | Yiling Pharmaceutical CO., LTD | Lianhua Qingwen, 6g | Y, Z20100040 | N |
| Wang et al. (2013) | Lianhua Qingwen granule | Yiling Pharmaceutical CO., LTD | Lianhua Qingwen, 6g | Y, Z20100040 | N |
| Xie (2013) | Lianhua Qingwen capsule | Yiling Pharmaceutical CO., LTD | Lianhua Qingwen, 0.35g | Y, Z20040063 | N |
| Xin (2013) | Lianhua Qingwen capsule | Yiling Pharmaceutical CO., LTD | Lianhua Qingwen, 0.35g | Y, Z20040063 | N |
| Xu (2013) | Lianhua Qingwen capsule | Yiling Pharmaceutical CO., LTD | Lianhua Qingwen, 0.35g | Y, Z20040063 | N |
| Ye et al. (2013) | Lianhua Qingwen capsule | Yiling Pharmaceutical CO., LTD | Lianhua Qingwen, 0.35g | Y, Z20040063 | N |
| Deng et al. (2012) | Lianhua Qingwen capsule | Yiling Pharmaceutical CO., LTD | Lianhua Qingwen, 0.35g | Y, Z20040063 | N |
| Duan (2012) | Lianhua Qingwen capsule | Yiling Pharmaceutical CO., LTD | Lianhua Qingwen, 0.35g | Y, Z20040063 | N |
| Lin et al. (2012) | Lianhua Qingwen capsule | Yiling Pharmaceutical CO., LTD | Lianhua Qingwen, 0.35g | Y, Z20040063 | N |
| Liu (2012) | Lianhua Qingwen capsule | Yiling Pharmaceutical CO., LTD | Lianhua Qingwen, 0.35g | Y, Z20040063 | N |
| Lu et al. (2012) | Lianhua Qingwen capsule | Yiling Pharmaceutical CO., LTD | Lianhua Qingwen, 0.35g | Y, Z20040063 | N |
| Tan et al. (2012) | Lianhua Qingwen capsule | Yiling Pharmaceutical CO., LTD | Lianhua Qingwen, 0.35g | Y, Z20040063 | N |
| Xu et al. (2012) | Lianhua Qingwen capsule | Yiling Pharmaceutical CO., LTD | Lianhua Qingwen, 0.35g | Y, Z20040063 | N |
| Yang (2012) | Lianhua Qingwen capsule | Yiling Pharmaceutical CO., LTD | Lianhua Qingwen, 0.35g | Y, Z20040063 | N |
| Yang et al. (2012) | Lianhua Qingwen capsule | Yiling Pharmaceutical CO., LTD | Lianhua Qingwen, 0.35g | Y, Z20040063 | N |

Supplementary Table 2 continued.

| **Study** | **Formulation** | **Source** | **Species, concentration** | **Quality control reported? (Y/N) *** | **Chemical analysis reported? (Y/N)** |
| --- | --- | --- | --- | --- | --- |
| Yao (2012) | Lianhua Qingwen capsule | Yiling Pharmaceutical CO., LTD | Lianhua Qingwen, 0.35g | Y, Z20040063 | N |
| Zhang et al. (2012) | Lianhua Qingwen capsule | Yiling Pharmaceutical CO., LTD | Lianhua Qingwen, 0.35g | Y, Z20040063 | N |
| Duan et al. (2011) | Lianhua Qingwen capsule | Yiling Pharmaceutical CO., LTD | Lianhua Qingwen, 0.35g | Y, Z20040063 | N |
| Cai et al. (2011) | Lianhua Qingwen granule | Yiling Pharmaceutical CO., LTD | Lianhua Qingwen, 6g | Y, Z20100040 | N |
| Cui et al. (2011) | Lianhua Qingwen capsule | Yiling Pharmaceutical CO., LTD | Lianhua Qingwen, 0.35g | Y, Z20040063 | N |
| Hu (2011) | Lianhua Qingwen capsule | Yiling Pharmaceutical CO., LTD | Lianhua Qingwen, 0.35g | Y, Z20040063 | N |
| Luo (2011a) | Lianhua Qingwen capsule | Yiling Pharmaceutical CO., LTD | Lianhua Qingwen, 0.35g | Y, Z20040063 | N |
| Luo (2011b) | Lianhua Qingwen capsule | Yiling Pharmaceutical CO., LTD | Lianhua Qingwen, 0.35g | Y, Z20040063 | N |
| Wang et al. (2011) | Lianhua Qingwen capsule | Yiling Pharmaceutical CO., LTD | Lianhua Qingwen, 0.35g | Y, Z20040063 | N |
| Xun et al. (2011) | Lianhua Qingwen capsule | Yiling Pharmaceutical CO., LTD | Lianhua Qingwen, 0.35g | Y, Z20040063 | N |
| Yang et al. (2011) | Lianhua Qingwen capsule | Yiling Pharmaceutical CO., LTD | Lianhua Qingwen, 0.35g | Y, Z20040063 | N |
| Yang (2011) | Lianhua Qingwen capsule | Yiling Pharmaceutical CO., LTD | Lianhua Qingwen, 0.35g | Y, Z20040063 | N |
| Yu et al. (2011) | Lianhua Qingwen capsule | Yiling Pharmaceutical CO., LTD | Lianhua Qingwen, 0.35g | Y, Z20040063 | N |
| Zhang et al. (2011) | Lianhua Qingwen capsule | Yiling Pharmaceutical CO., LTD | Lianhua Qingwen, 0.35g | Y, Z20040063 | N |
| Zhou (2011) | Lianhua Qingwen capsule | Yiling Pharmaceutical CO., LTD | Lianhua Qingwen, 0.35g | Y, Z20040063 | N |
| Lai et al. (2010) | Lianhua Qingwen capsule | Yiling Pharmaceutical CO., LTD | Lianhua Qingwen, 0.35g | Y, Z20040063 | N |
| Liu et al. (2010) | Lianhua Qingwen capsule | Yiling Pharmaceutical CO., LTD | Lianhua Qingwen, 0.35g | Y, Z20040063 | N |
| Ma et al. (2010) | Lianhua Qingwen capsule | Yiling Pharmaceutical CO., LTD | Lianhua Qingwen, 0.35g | Y, Z20040063 | N |
| Ouyang et al. (2010) | Lianhua Qingwen capsule | Yiling Pharmaceutical CO., LTD | Lianhua Qingwen, 0.35g | Y, Z20040063 | N |
| Wei et al. (2010) | Lianhua Qingwen capsule | Yiling Pharmaceutical CO., LTD | Lianhua Qingwen, 0.35g | Y, Z20040063 | N |
| Zhang et al. (2010a) | Lianhua Qingwen capsule | Yiling Pharmaceutical CO., LTD | Lianhua Qingwen, 0.35g | Y, Z20040063 | N |
| Zhang et al. (2010b) | Lianhua Qingwen capsule | Yiling Pharmaceutical CO., LTD | Lianhua Qingwen, 0.35g | Y, Z20040063 | N |
| Zheng (2010) | Lianhua Qingwen capsule | Yiling Pharmaceutical CO., LTD | Lianhua Qingwen, 0.35g | Y, Z20040063 | N |

Supplementary Table 2 continued.

| **Study** | **Formulation** | **Source** | **Species, concentration** | **Quality control reported? (Y/N) *** | **Chemical analysis reported? (Y/N)** |
| --- | --- | --- | --- | --- | --- |
| Li et al. (2009a) | Lianhua Qingwen capsule | Yiling Pharmaceutical CO., LTD | Lianhua Qingwen, 0.35g | Y, Z20040063 | N |
| Li et al. (2009b) | Lianhua Qingwen granule | Yiling Pharmaceutical CO., LTD | Lianhua Qingwen, 6g | Y, Z20100040 | N |
| Hu et al. (2008) | Lianhua Qingwen capsule | Yiling Pharmaceutical CO., LTD | Lianhua Qingwen, 0.35g | Y, Z20040063 | N |
| Wang et al. (2008a) | Lianhua Qingwen capsule | Yiling Pharmaceutical CO., LTD | Lianhua Qingwen, 0.35g | Y, Z20040063 | N |
| Wang et al. (2008b) | Lianhua Qingwen capsule | Yiling Pharmaceutical CO., LTD | Lianhua Qingwen, 0.35g | Y, Z20040063 | N |
| Wang et al. (2008c) | Lianhua Qingwen capsule | Yiling Pharmaceutical CO., LTD | Lianhua Qingwen, 0.35g | Y, Z20040063 | N |
| Wu et al. (2006) | Lianhua Qingwen capsule | Yiling Pharmaceutical CO., LTD | Lianhua Qingwen, 0.35g | Y, Z20040063 | N |
| Zuo et al. (2006) | Lianhua Qingwen capsule | Yiling Pharmaceutical CO., LTD | Lianhua Qingwen, 0.35g | Y, Z20040063 | N |
| Yang et al. (2005a) | Lianhua Qingwen capsule | Yiling Pharmaceutical CO., LTD | Lianhua Qingwen, 0.35g | Y, Z20040063 | N |
| Yang et al., (2005b) | Lianhua Qingwen capsule | Yiling Pharmaceutical CO., LTD | Lianhua Qingwen, 0.35g | Y, Z20040063 | N |

*：Approval number of The National Medical Products Administration of China was provided.
