# Supplementary material for: The Adverse Reactions of Lianhua Qingwen Capsule/Granule Compared With Conventional Drug in Clinical Application: A Meta-Analysis
Source: Front Pharmacol. 2022 Jan 27;13:764774. doi: 10.3389/fphar.2022.764774 (PMC8830515; doi:10.3389/fphar.2022.764774)
Supplement: Supplementary file 3 [file Image1.pdf]

Supplementary Figures

1. **Supplementary Figure 1.** Forest plots of statistically significant differences in the incidence of adverse reactions between Lianhua Qingwen group and the conventional drug group.

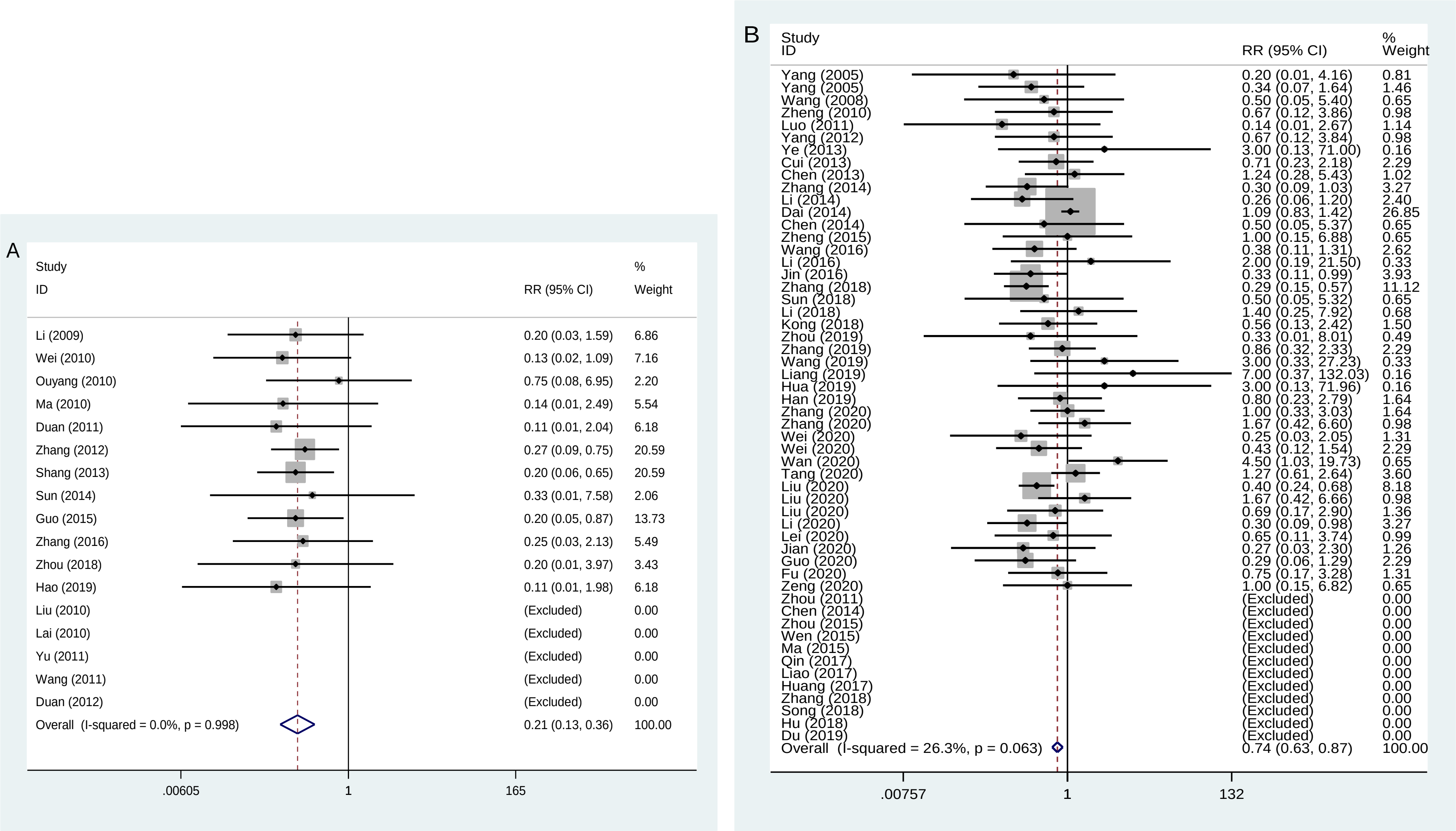

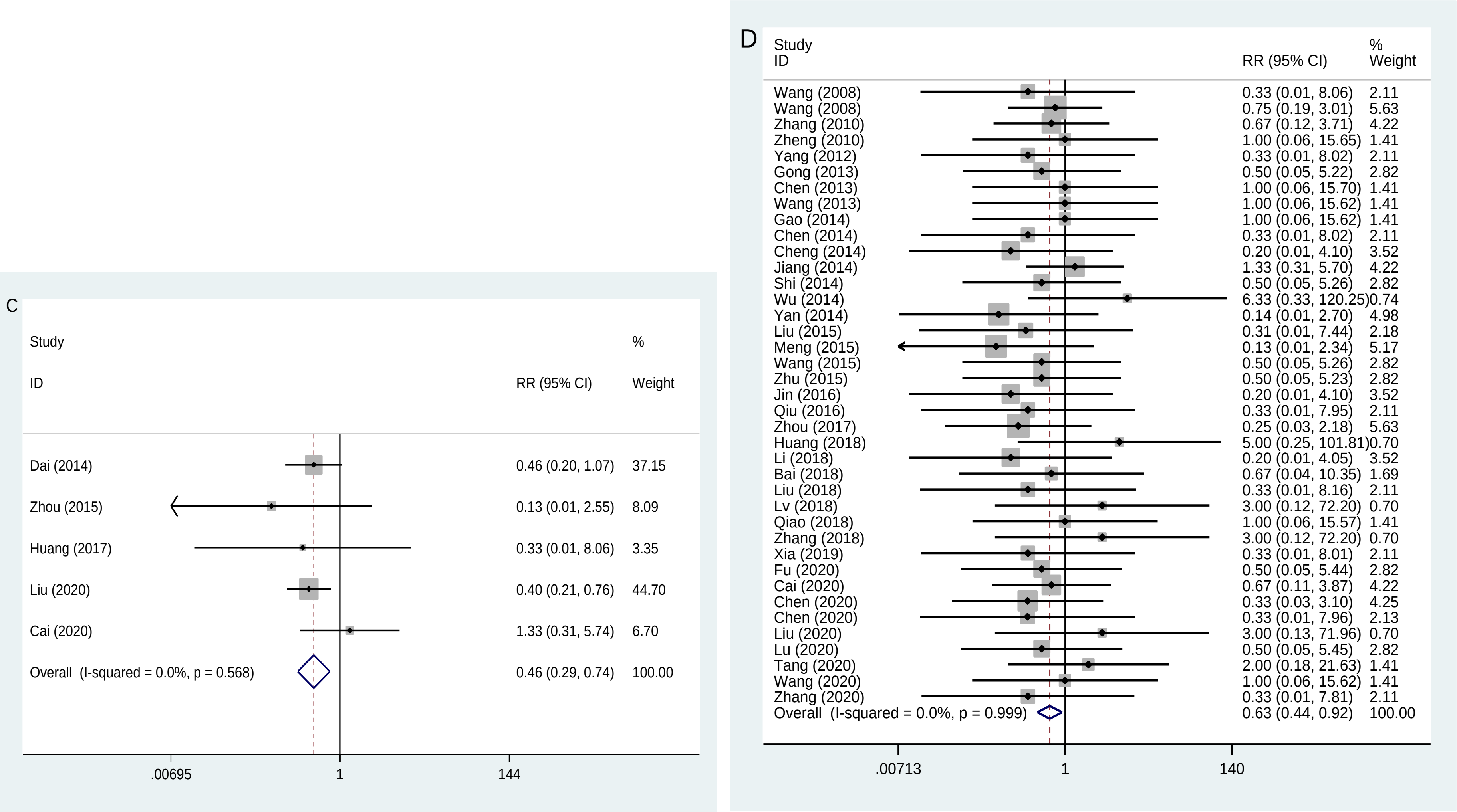

C
Study
ID
RR (95% CI)
%
Weight
Dai (2014) 0.46 (0.20, 1.07) 37.15
Zhou (2015) 0.13 (0.01, 2.55) 8.09
Huang (2017) 0.33 (0.01, 8.06) 3.35
Liu (2020) 0.40 (0.21, 0.76) 44.70
Cai (2020) 1.33 (0.31, 5.74) 6.70
Overall (I-squared = 0.0%, p = 0.568) 0.46 (0.29, 0.74) 100.00
.00695
1
144
D
Study
ID
RR (95% CI)
%
Weight
Wang (2008) 0.33 (0.01, 8.06) 2.11
Wang (2008) 0.75 (0.19, 3.01) 5.63
Zhang (2010) 0.67 (0.12, 3.71) 4.22
Zheng (2010) 1.00 (0.06, 15.65) 1.41
Yang (2012) 0.33 (0.01, 8.02) 2.11
Gong (2013) 0.50 (0.05, 5.22) 2.82
Chen (2013) 1.00 (0.06, 15.70) 1.41
Wang (2013) 1.00 (0.06, 15.62) 1.41
Gao (2014) 1.00 (0.06, 15.62) 1.41
Chen (2014) 0.33 (0.01, 8.02) 2.11
Cheng (2014) 0.20 (0.01, 4.10) 3.52
Jiang (2014) 1.33 (0.31, 5.70) 4.22
Shi (2014) 0.50 (0.05, 5.26) 2.82
Wu (2014) 6.33 (0.33, 120.25)0.74
Yan (2014) 0.14 (0.01, 2.70) 4.98
Liu (2015) 0.31 (0.01, 7.44) 2.18
Meng (2015) 0.13 (0.01, 2.34) 5.17
Wang (2015) 0.50 (0.05, 5.26) 2.82
Zhu (2015) 0.50 (0.05, 5.23) 2.82
Jin (2016) 0.20 (0.01, 4.10) 3.52
Qiu (2016) 0.33 (0.01, 7.95) 2.11
Zhou (2017) 0.25 (0.03, 2.18) 5.63
Huang (2018) 5.00 (0.25, 101.81)0.70
Li (2018) 0.20 (0.01, 4.05) 3.52
Bai (2018) 0.67 (0.04, 10.35) 1.69
Liu (2018) 0.33 (0.01, 8.16) 2.11
Lv (2018) 3.00 (0.12, 72.20) 0.70
Qiao (2018) 1.00 (0.06, 15.57) 1.41
Zhang (2018) 3.00 (0.12, 72.20) 0.70
Xia (2019) 0.33 (0.01, 8.01) 2.11
Fu (2020) 0.50 (0.05, 5.44) 2.82
Cai (2020) 0.67 (0.11, 3.87) 4.22
Chen (2020) 0.33 (0.03, 3.10) 4.25
Chen (2020) 0.33 (0.01, 7.96) 2.13
Liu (2020) 3.00 (0.13, 71.96) 0.70
Lu (2020) 0.50 (0.05, 5.45) 2.82
Tang (2020) 2.00 (0.18, 21.63) 1.41
Wang (2020) 1.00 (0.06, 15.62) 1.41
Zhang (2020) 0.33 (0.01, 7.81) 2.11
Overall (I-squared = 0.0%, p = 0.999) 0.63 (0.44, 0.92) 100.00
.00713
1
140

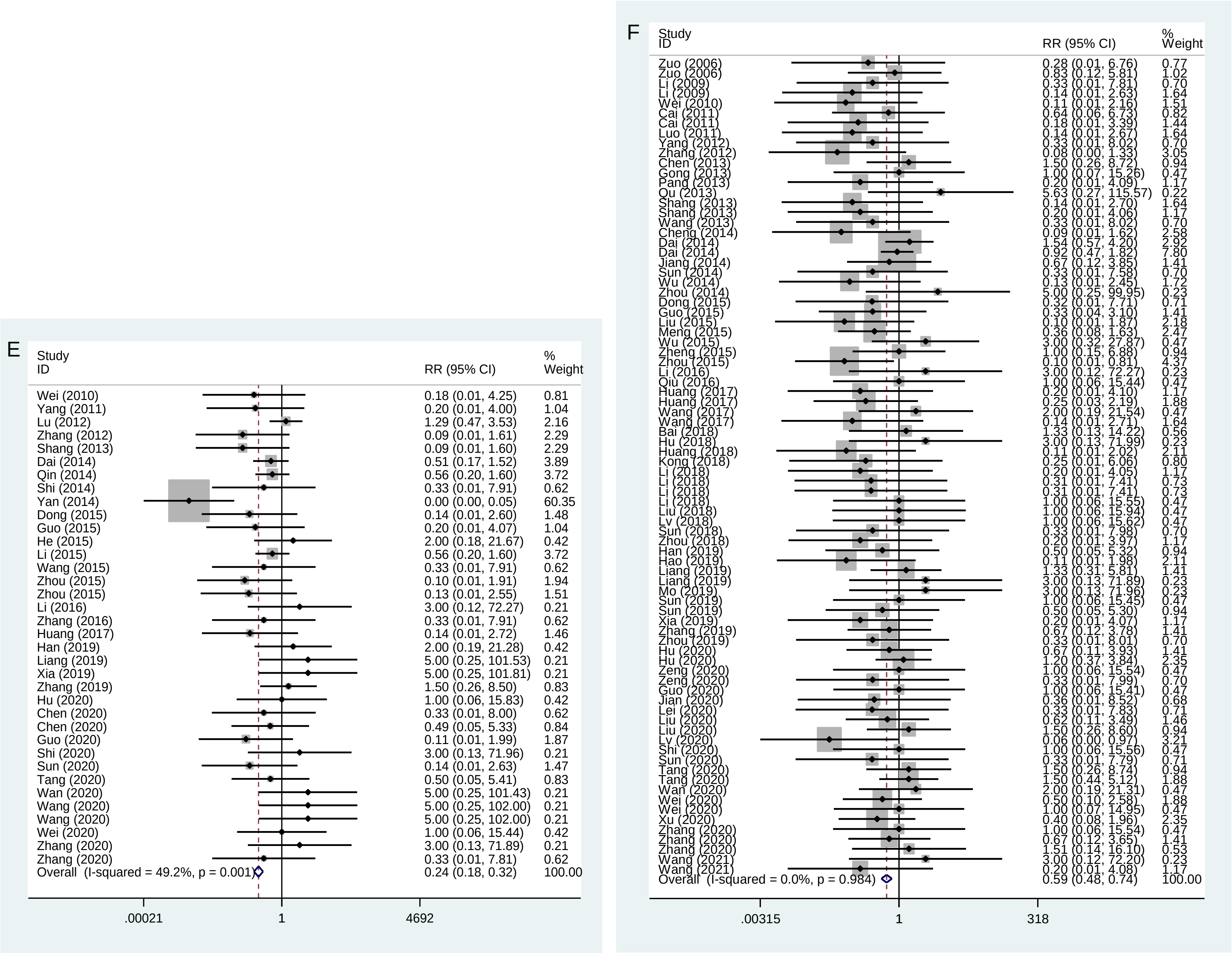

E
Study
ID
RR (95% CI)
%
Weight
Wei (2010) 0.18 (0.01, 4.25) 0.81
Yang (2011) 0.20 (0.01, 4.00) 1.04
Lu (2012) 1.29 (0.47, 3.53) 2.16
Zhang (2012) 0.09 (0.01, 1.61) 2.29
Shang (2013) 0.09 (0.01, 1.60) 2.29
Dai (2014) 0.51 (0.17, 1.52) 3.89
Qin (2014) 0.56 (0.20, 1.60) 3.72
Shi (2014) 0.33 (0.01, 7.91) 0.62
Yan (2014) 0.00 (0.00, 0.05) 60.35
Dong (2015) 0.14 (0.01, 2.60) 1.48
Guo (2015) 0.20 (0.01, 4.07) 1.04
He (2015) 2.00 (0.18, 21.67) 0.42
Li (2015) 0.56 (0.20, 1.60) 3.72
Wang (2015) 0.33 (0.01, 7.91) 0.62
Zhou (2015) 0.10 (0.01, 1.91) 1.94
Zhou (2015) 0.13 (0.01, 2.55) 1.51
Li (2016) 3.00 (0.12, 72.27) 0.21
Zhang (2016) 0.33 (0.01, 7.91) 0.62
Huang (2017) 0.14 (0.01, 2.72) 1.46
Han (2019) 2.00 (0.19, 21.28) 0.42
Liang (2019) 5.00 (0.25, 101.53) 0.21
Xia (2019) 5.00 (0.25, 101.81) 0.21
Zhang (2019) 1.50 (0.26, 8.50) 0.83
Hu (2020) 1.00 (0.06, 15.83) 0.42
Chen (2020) 0.33 (0.01, 8.00) 0.62
Chen (2020) 0.49 (0.05, 5.33) 0.84
Guo (2020) 0.11 (0.01, 1.99) 1.87
Shi (2020) 3.00 (0.13, 71.96) 0.21
Sun (2020) 0.14 (0.01, 2.63) 1.47
Tang (2020) 0.50 (0.05, 5.41) 0.83
Wan (2020) 5.00 (0.25, 101.43) 0.21
Wang (2020) 5.00 (0.25, 102.00) 0.21
Wang (2020) 5.00 (0.25, 102.00) 0.21
Wei (2020) 1.00 (0.06, 15.44) 0.42
Zhang (2020) 3.00 (0.13, 71.89) 0.21
Zhang (2020) 0.33 (0.01, 7.81) 0.62
Overall (I-squared = 49.2%, p = 0.001) 0.24 (0.18, 0.32) 100.00
.00021
1
4692
F
Study
ID
RR (95% CI)
%
Weight
Zuo (2006) 0.28 (0.01, 6.76) 0.77
Zuo (2006) 0.83 (0.12, 5.81) 1.02
Li (2009) 0.33 (0.01, 7.81) 0.70
Li (2009) 0.14 (0.01, 2.63) 1.64
Wei (2010) 0.11 (0.01, 2.16) 1.51
Cai (2011) 0.64 (0.06, 6.73) 0.82
Cai (2011) 0.18 (0.01, 3.39) 1.44
Luo (2011) 0.14 (0.01, 2.67) 1.64
Yang (2012) 0.33 (0.01, 8.02) 0.70
Zhang (2012) 0.08 (0.00, 1.33) 3.05
Chen (2013) 1.50 (0.26, 8.72) 0.94
Gong (2013) 1.00 (0.07, 15.26) 0.47
Pang (2013) 0.20 (0.01, 4.09) 1.17
Qu (2013) 5.63 (0.27, 115.57) 0.22
Shang (2013) 0.14 (0.01, 2.70) 1.64
Shang (2013) 0.20 (0.01, 4.06) 1.17
Wang (2013) 0.33 (0.01, 8.02) 0.70
Cheng (2014) 0.09 (0.01, 1.62) 2.58
Dai (2014) 1.54 (0.57, 4.20) 2.92
Dai (2014) 0.92 (0.47, 1.82) 7.80
Jiang (2014) 0.67 (0.12, 3.85) 1.41
Sun (2014) 0.33 (0.01, 7.58) 0.70
Wu (2014) 0.13 (0.01, 2.45) 1.72
Zhou (2014) 5.00 (0.25, 99.95) 0.23
Dong (2015) 0.32 (0.01, 7.71) 0.71
Guo (2015) 0.33 (0.04, 3.10) 1.41
Liu (2015) 0.10 (0.01, 1.87) 2.18
Meng (2015) 0.36 (0.08, 1.63) 2.47
Wu (2015) 3.00 (0.32, 27.87) 0.47
Zheng (2015) 1.00 (0.15, 6.88) 0.94
Zhou (2015) 0.10 (0.01, 0.81) 4.37
Li (2016) 3.00 (0.12, 72.27) 0.23
Qiu (2016) 1.00 (0.06, 15.44) 0.47
Huang (2017) 0.20 (0.01, 4.10) 1.17
Huang (2017) 0.25 (0.03, 2.19) 1.88
Wang (2017) 2.00 (0.19, 21.54) 0.47
Wang (2017) 0.14 (0.01, 2.71) 1.64
Bai (2018) 1.33 (0.13, 14.22) 0.56
Hu (2018) 3.00 (0.13, 71.99) 0.23
Huang (2018) 0.11 (0.01, 2.02) 2.11
Kong (2018) 0.25 (0.01, 6.06) 0.80
Li (2018) 0.20 (0.01, 4.05) 1.17
Li (2018) 0.31 (0.01, 7.41) 0.73
Li (2018) 0.31 (0.01, 7.41) 0.73
Li (2018) 1.00 (0.06, 15.55) 0.47
Liu (2018) 1.00 (0.06, 15.94) 0.47
Lv (2018) 1.00 (0.06, 15.62) 0.47
Sun (2018) 0.33 (0.01, 7.98) 0.70
Zhou (2018) 0.20 (0.01, 3.97) 1.17
Han (2019) 0.50 (0.05, 5.32) 0.94
Hao (2019) 0.11 (0.01, 1.98) 2.11
Liang (2019) 1.33 (0.31, 5.81) 1.41
Liang (2019) 3.00 (0.13, 71.89) 0.23
Mo (2019) 3.00 (0.13, 71.96) 0.23
Sun (2019) 1.00 (0.06, 15.45) 0.47
Sun (2019) 0.50 (0.05, 5.30) 0.94
Xia (2019) 0.20 (0.01, 4.07) 1.17
Zhang (2019) 0.67 (0.12, 3.78) 1.41
Zhou (2019) 0.33 (0.01, 8.01) 0.70
Hu (2020) 0.67 (0.11, 3.93) 1.41
Hu (2020) 1.20 (0.37, 3.84) 2.35
Zeng (2020) 1.00 (0.06, 15.54) 0.47
Zeng (2020) 0.33 (0.01, 7.99) 0.70
Guo (2020) 1.00 (0.06, 15.41) 0.47
Jian (2020) 0.36 (0.01, 8.52) 0.68
Lei (2020) 0.33 (0.01, 7.83) 0.71
Liu (2020) 0.62 (0.11, 3.49) 1.46
Liu (2020) 1.50 (0.26, 8.60) 0.94
Lv (2020) 0.06 (0.00, 0.97) 3.21
Shi (2020) 1.00 (0.06, 15.56) 0.47
Sun (2020) 0.33 (0.01, 7.79) 0.71
Tang (2020) 1.50 (0.26, 8.74) 0.94
Tang (2020) 1.50 (0.44, 5.12) 1.88
Wan (2020) 2.00 (0.19, 21.31) 0.47
Wei (2020) 0.50 (0.10, 2.58) 1.88
Wei (2020) 1.00 (0.07, 14.95) 0.47
Xu (2020) 0.40 (0.08, 1.96) 2.35
Zhang (2020) 1.00 (0.06, 15.54) 0.47
Zhang (2020) 0.67 (0.12, 3.65) 1.41
Zhang (2020) 1.51 (0.14, 16.10) 0.53
Wang (2021) 3.00 (0.12, 72.20) 0.23
Wang (2021) 0.20 (0.01, 4.08) 1.17
Overall (I-squared = 0.0%, p = 0.984) 0.59 (0.48, 0.74) 100.00
.00315
1
318

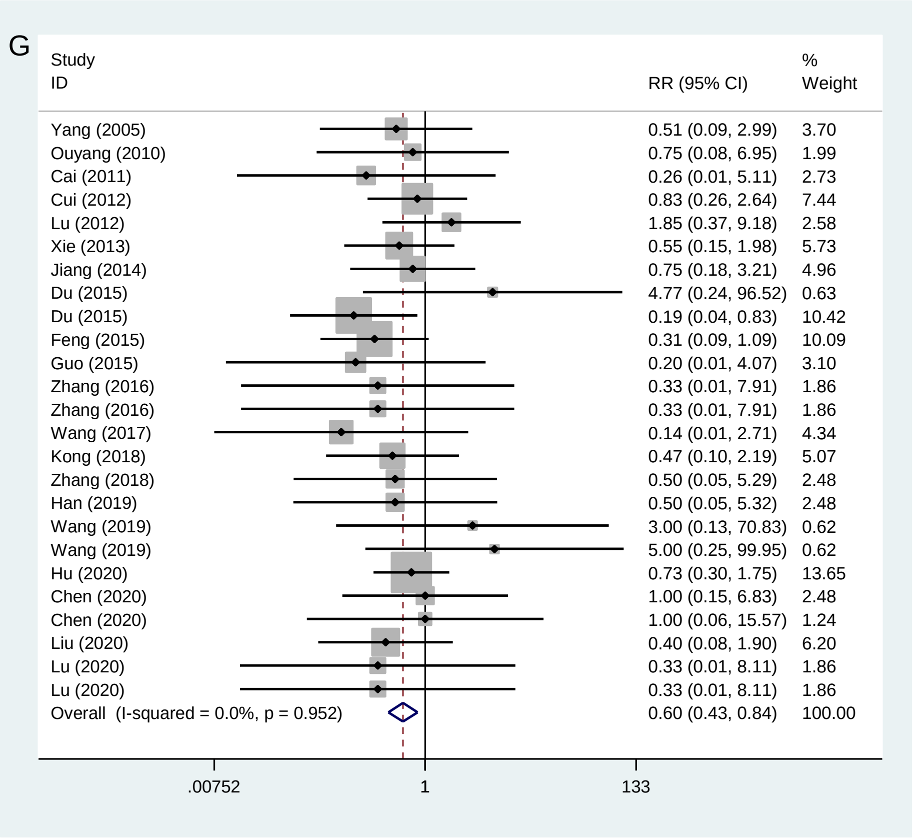

A, Influenza A (H1N1) of diseases evaluation; B, Influenza of diseases evaluation; C, Respiratory system damage of security index evaluation; D, Skin and its appendage damage of security index evaluation; E, Nervous system damage of security index evaluation; F, Gastrointestinal system damage (Nausea or vomiting) of security index evaluation; G, Other adverse reactions of security index evaluation.
